# Glycan Masking of *Plasmodium vivax* Duffy Binding Protein for Probing Protein Binding Function and Vaccine Development

**DOI:** 10.1371/journal.ppat.1003420

**Published:** 2013-06-13

**Authors:** Sowmya Sampath, Chris Carrico, Joel Janes, Sairam Gurumoorthy, Claire Gibson, Martin Melcher, Chetan E. Chitnis, Ruobing Wang, William R. Schief, Joseph D. Smith

**Affiliations:** 1 Seattle Biomedical Research Institute, Seattle, Washington, United States of America; 2 Department of Biochemistry, University of Washington, Seattle, Washington, United States of America; 3 Basic Sciences Division, Fred Hutchinson Cancer Research Center, Seattle, Washington, United States of America; 4 International Centre for Genetic Engineering and Biotechnology (ICGEB), New Delhi, India; 5 Department of Immunology and Microbial Science and IAVI Neutralizing Antibody Center, The Scripps Research Institute, La Jolla, California, United States of America; 6 Center for HIV/AIDS Vaccine Immunology and Immunogen Discovery, The Scripps Research Institute, La Jolla, California, United States of America; 7 Department of Pathobiology, University of Washington, Seattle, Washington, United States of America; Case Western Reserve University, United States of America

## Abstract

Glycan masking is an emerging vaccine design strategy to focus antibody responses to specific epitopes, but it has mostly been evaluated on the already heavily glycosylated HIV gp120 envelope glycoprotein. Here this approach was used to investigate the binding interaction of *Plasmodium vivax* Duffy Binding Protein (PvDBP) and the Duffy Antigen Receptor for Chemokines (DARC) and to evaluate if glycan-masked PvDBPII immunogens would focus the antibody response on key interaction surfaces. Four variants of PVDBPII were generated and probed for function and immunogenicity. Whereas two PvDBPII glycosylation variants with increased glycan surface coverage distant from predicted interaction sites had equivalent binding activity to wild-type protein, one of them elicited slightly better DARC-binding-inhibitory activity than wild-type immunogen. Conversely, the addition of an N-glycosylation site adjacent to a predicted PvDBP interaction site both abolished its interaction with DARC and resulted in weaker inhibitory antibody responses. PvDBP is composed of three subdomains and is thought to function as a dimer; a meta-analysis of published PvDBP mutants and the new DBPII glycosylation variants indicates that critical DARC binding residues are concentrated at the dimer interface and along a relatively flat surface spanning portions of two subdomains. Our findings suggest that DARC-binding-inhibitory antibody epitope(s) lie close to the predicted DARC interaction site, and that addition of N-glycan sites distant from this site may augment inhibitory antibodies. Thus, glycan resurfacing is an attractive and feasible tool to investigate protein structure-function, and glycan-masked PvDBPII immunogens might contribute to *P. vivax* vaccine development.

## Author Summary

An important goal of many vaccine efforts is to inhibit pathogen invasion of host cells, but few approaches exist to target vaccine antibodies on invasion blocking epitopes. Glycan masking is a vaccine design strategy to hide protein surfaces with carbohydrates and focus antibodies on exposed surfaces. This approach has mostly been evaluated on the heavily glycosylated HIV envelope glycoprotein, but it has never been tested on eukaryotic pathogens, such as *Plasmodium*, which have limited N-glycosylation machinery and therefore may provide a better platform to explore this strategy. Here, we used glycan masking to investigate the binding interaction between *Plasmodium vivax* Duffy binding protein (PvDBP) and the Duffy Antigen Receptor for Chemokines (DARC). This study showed that addition of an N-glycan site in a predicted host interaction surface abolished binding and potentially covered up an inhibitory antibody epitope. In contrast, addition of multiple N-glycan sites distant from predicted interaction surfaces did not inhibit binding but did slightly enhance elicitation of inhibitory antibodies. This analysis shows that glycan resurfacing offers an integrated approach to characterize protein function and immunogenicity and that glycan resurfacing of PvDBPII immunogens may have utility in *P. vivax*-malaria vaccine development.

## Introduction

*Plasmodium vivax* invasion of human reticulocytes is strongly dependent on an interaction between the *P. vivax* Duffy Binding Protein (PvDBP) and the Duffy Antigen Receptor for Chemokines (DARC) on the reticulocyte surface [1]. DARC-negative individuals are highly resistant to *P. vivax* infection [2] and the DARC-null phenotype has independently arisen in different human populations [3], [4]. Although an alternative pathway of *P. vivax* invasion has recently been described [5], [6], DARC-null carriers have reduced susceptibility to *P. vivax* infection [4], [7] and the FyA DARC allele shows reduced binding to PvDBP and is more susceptible to antibody blocking [8]. Thus, the PvDBP-DARC interaction has a critical role in *P. vivax* infection making it an attractive vaccine target.

The molecular mechanisms of PvDBP-DARC binding and immune evasion are only partially understood. PvDBP is a member of the Erythrocyte Binding-Like (EBL) protein superfamily [9]–[11]. The extracellular region of PvDBP has been divided into six regions [10], of which DARC binding has been localized to region II (PvDBPII) [12]. The structure of PvDBPII [13] and a related Duffy binding protein of the simian malaria parasite *P. knowlesi* (Pkα-DBL) [14] has been solved and is composed of three subdomains – subdomain 1, 2 and 3. PvDBPII binds to the N-terminal 65 residues of DARC with a sulfated tyrosine on DARC at position 41 having a critical role in binding [15], [16].

**Figure 1:**
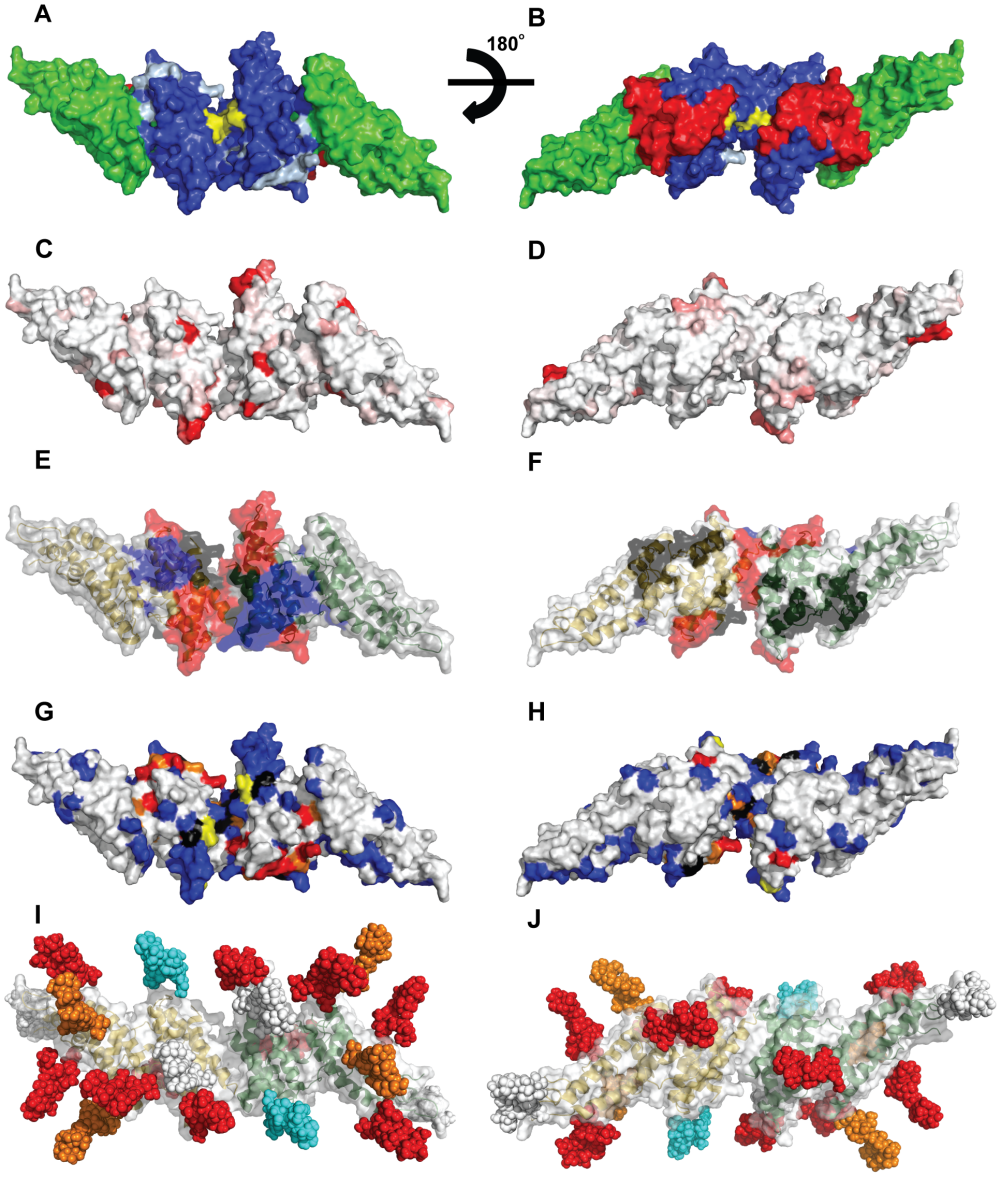
Design of surface re-engineered PvDBPII recombinant proteins containing additional N-linked glycan residues. (A–B) Subdomains and DARC binding models. Subdomain 1(red), subdomain 2 (blue), and subdomain 3 (green). Critical binding residues for model 1 are colored light blue and for model 2 are colored yellow. (C–D) Fractional Shannon entropy values [70] from 0.000 (white) to 0.243 (red) for sequence polymorphism over the PvDBPII surface as compared with maximally entropic distribution over all amino acids. (E–F) Epitopes recognized by blocking antibodies [20]; black (low inhibitory), blue (medium inhibitory), red (high inhibitory). (G–H) Meta-analysis of mutations that reduce or do not affect the PvDBP-DARC interaction: blue residues (no effect); yellow residues (minor); orange residues (moderate); red residues (major); black residues, differences between studies (see Table S1). (I–J) Location of engineered N-glycosylation sites modeled as high mannose forms; white (wild type), cyan (STBP glycan), Orange (P1 and Max), red (Max). All images modeled in PyMol on the PvDBPII dimer structure [13], viewing opposite ends of the dimeric two-fold axis for subpanels I and II; missing density in the crystal structure has been left missing, though it contains polymorphic, inhibitory, antibody recognition and glycan-bearing sites. PvDBPII monomers are colored yellow and green in panels E, F, I, and J.

Although the PvDBP structure has been solved, the precise extent of the DARC binding “footprint” remains unclear [17], [18], and there is limited understanding of the epitopes for DARC-inhibitory antibodies on PvDBP. Two different PvDBP-DARC binding models have been proposed (Figure 1). The “just in time” model hypothesizes that PvDBP engages DARC in a monomer-monomer interaction and that binding occurs so rapidly that the binding site is not under strong antibody attack [14]. In this model, the putative sulfotryosine binding pocket is located at a relatively flat surface in subdomain 2, on the opposite surface from a cluster of polymorphic residues [1]. It has also been proposed that adjacent residues from subdomain 1 form the sulfotyrosine-binding pocket, in an analogous manner to how sulphated tyrosines facilitate the gp120-CCR5 interaction during HIV invasion [19]. For convenience, we will refer to the flat surface on subdomain 2 as the sulfotryosine binding pocket (STBP) when referring to this location (light blue residues in Figure 1), although this remains to be established. The second model, termed “receptor-mediated ligand dimerization”, hypothesizes that DARC binding drives dimerization between PvDBP monomers and that the sulfotyrosine-binding pocket and DARC binding pocket are formed at the dimer interface, at distinct residues from the “just in time” model [13].

These two binding models are not necessarily exclusive but lead to different predictions about how antibodies may inhibit *P. vivax* invasion of reticulocytes. High titer and broadly neutralizing antibodies to PvDBP are only found after repeated infection [20]–[23], indicating that PvDBP is not highly immunogenic during infection. Naturally acquired inhibitory antibodies map to the dimer interface [13], [20], suggesting that one mechanism of antibody action may be interference with receptor dimerization. Other inhibitory epitopes have been mapped to the highly conserved subdomain 3 [24] and a polymorphic residue located near the putative STBP in subdomain 2 [25], indicating that some inhibitory epitopes may be selected for immune evasion. *P. vivax* dependence on DARC for invasion and its restrictive preference for reticulocytes supports PvDBP vaccine development. However, the rapid kinetics of parasite invasion (less than one minute) [26], [27] combined with PvDBP polymorphism [28], [29] pose challenges for vaccine development that may be addressable by the rational redesign of PvDBPII for improved immunogenic properties.

Structural vaccinology is an emerging strategy that uses three dimensional structure to guide immunogen design to improve functional antibody responses. Viral enveloped pathogens, like HIV, use a number of mechanisms to evade immune surveillance, one of them being glycan shielding of epitopes [30]. Glycan masking, or surface glycoengineering, has been applied to HIV vaccine development as a means to shield regions of the gp120 surface from antibody recognition, but this has not resulted in elicitation of more potent or more broadly-cross-reactive neutralizing antibody responses [31]–[35]. However, gp120 is one of the most heavily glycosylated proteins in nature, so it may not offer the optimum protein scaffold to evaluate this strategy. Conversely, many deep branching eukaryotic pathogens, such as *Plasmodium* and *Giardia*, have limited N-glycosylation machinery [36], and consequently are not as highly evolved for glycan shielding. Thus, malaria proteins may be more amenable to glycan masking. Here, glycan masking was employed as a strategy both to investigate the PvDBP-DARC binding model and to evaluate whether it increased DARC-binding-inhibitory antibodies.

## Results

### Design of surface re-engineered PvDBPII recombinant proteins containing additional N-linked glycan sites

The monomeric crystal structure of the *P. knowlesi* α DBL domain [14] was used to guide glycan masking of PvDBPII recombinant proteins. Defined secondary structure regions in PvDBPII are largely helical [13] – subdomain 1 contains a β-hairpin, subdomain 2 is a four-helix bundle, and subdomain 3 contains a double helical bundle (Figure 1). N-glycosylation sites occur most frequently in regions of change in secondary structure, and least frequently on helices than any other secondary structural element [37]. The PvDBPII dimer interface is formed between contacts across helical domains in the two monomers [13], and consequently N-glycan acceptor sites were not added to this location. Instead, glycosylation sites were mostly added on surface exposed loops and the ends of helices that were not expected to contribute to invasion blocking epitopes but were expected to be accessible to antibodies.

**Figure 2:**
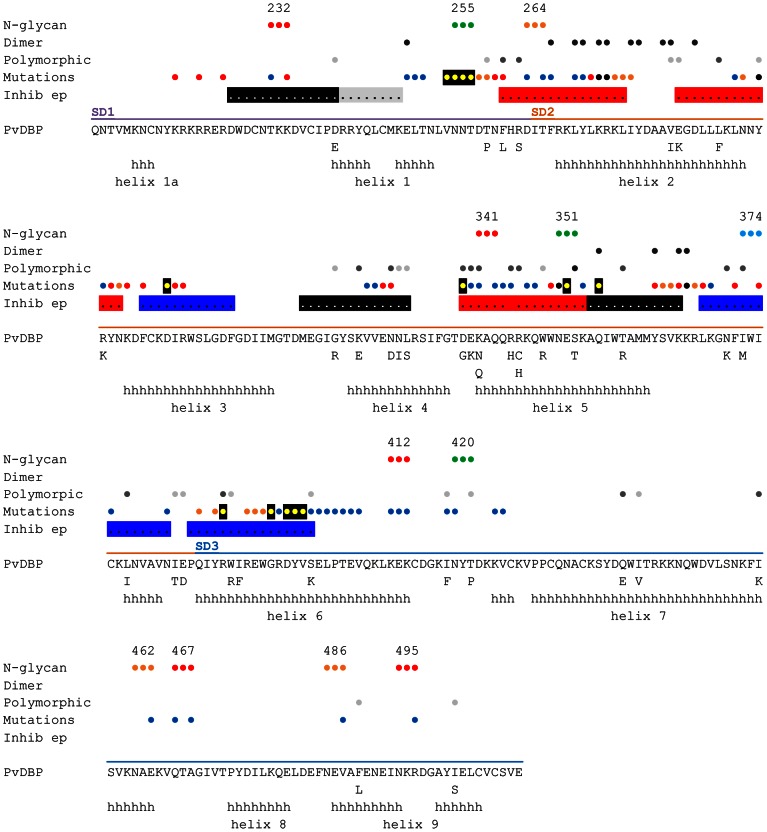
Summary of PvDBP polymorphism, inhibitory epitopes, and residues impacting DARC binding. The sequence of the solved Sal1strain PvDBP variant crystal structure [13] is shown. Polymorphic amino acids from 129 PvDBP sequences [28], [29] are listed below the Sal1 sequence. Alpha helices in the PvDBP crystal structure [13] are indicated by “h” and labeled helix 1a to helix 9 according to convention [71]. Circles above the line-up indicate important residues. N-glycosylation sites are numbered according to Table 1 and colored green (wild type), blue (STBP glycan), orange (P1 and Max), and red (Max). Dimer interface [13] – black circles; polymorphism – light grey (rare, <10% of sequences), dark grey (>10% of sequences); mutations that effect DARC binding from this study and others [13], [17]–[19], are colored blue (no effect), yellow with black shadowing (minor), orange (moderate), red (major), and black, differences between studies (see Table S1); linear epitopes targeted by inhibitory antibodies [20] – black or grey shading (low inhibitory), blue shading (medium inhibitory), red shading (high inhibitory).

The PvDBP sequence from the *P. vivax* Salvador 1 strain (Sal1) contains three potential N-glycosylation sites at positions 255, 351 and 420 (Figure 2). As the three native positions were located at the surface of subdomain 2 or subdomain 3 of PvDBPII distinct from the putative DARC interaction surface or dimerization surfaces (Figure 1), all three were retained in all of the hyperglycosylated variant sequences (Table 1). Three DBPII glycosylation variants were designed containing a total of four, six and 11 N-linked glycans, called “STBP glycan”, “P1” and “Max” respectively (Table 1, Figure S1). To evaluate the PvDBP-DARC interaction model [14], the “STBP glycan” was generated by adding an N-glycan site at position 374, where it would potentially cover the predicted sulfotyrosine binding pocket in subdomain 2 (model 1, Figure 1) and inhibit binding to reticulocytes. The remaining two glycan variants were designed to shield polymorphic residues in PvDBP but retain exposure of proposed interaction surfaces for DARC binding and/or PvDBP dimerization (Figures 1 and 2). There are at least 36 polymorphic positions in PvDBPII [28], [29], but many sequence variants are extremely rare (Figure S2). The majority of polymorphic residues are dimorphic and localized to subdomain 2 (Figure 2). Critical DARC binding residues and several linear antibody inhibitory epitopes have been mapped to the N-terminus of subdomain 1, helices 2–5, and the connecting loop between subdomains 2 and 3 (Figure 2) [17]–[20]. P1, the second DBPII glycosylation variant was generated by adding three extra N-linked glycosylation sites at positions 264, 462, 486. The addition of an N-glycan site at 264, along with the native N- glycan sites at position 255 and 351 was designed to shield polymorphic residues that are present in subdomain 2 (Figures 1 and 2). N-glycan sites at position 462 and 486 along with the native glycosylation site at position 420 were designed to cover a portion of subdomain 3 (Figures 1 and 2). A third DBPII glycosylation variant, “Max”, contained the six N-glycan sites on P1, plus five additional N-glycan sites at 232, 341, 412, 467 and 495, thus bringing the consensus N-linked glycan sites in Max to 11. Addition of N-glycan sites at 412, 462, 467, 486, and 495, along with the native site at 420, were designed to cover all the surface accessible loops on subdomain 3. Further addition of N-glycan sites at positions 264 and 341, along with the native N-glycosylation sites at 255 and 351, were designed to cover all loops in subdomain 2 including a polymorphic loop between helices 1 and 2 and the highly polymorphic helix 5 (Figures 1 and 2). The Max glycosylation variant, with 11 N-glycans, should have a large surface area of PvDBPII shielded by glycosylation (Figure 1), but none of the N-glycosylation sites are at the dimer interface or predicted to interfere with DARC binding.

**Table 1:** PvDBPII recombinant proteins.

Immunogen	No. of glycosylation	N-glycosylation sites	Molecular mass (kDa)	Percent Binding
Wild type (HEK293)	3	N255, N351, N420	49	97
STBP glycan	4	N255, N351, N374, N420	51	4
P1	6	N255, N264, N351, N420, N462, N486	55	75
Max	11	N232, N255, N264, N341, N351, N412, N420, N462, N467, N486, N495	65	88

### Expression and purification of surface re-engineered PvDBPII recombinant proteins containing additional N-linked glycan residues

**Figure 3:**
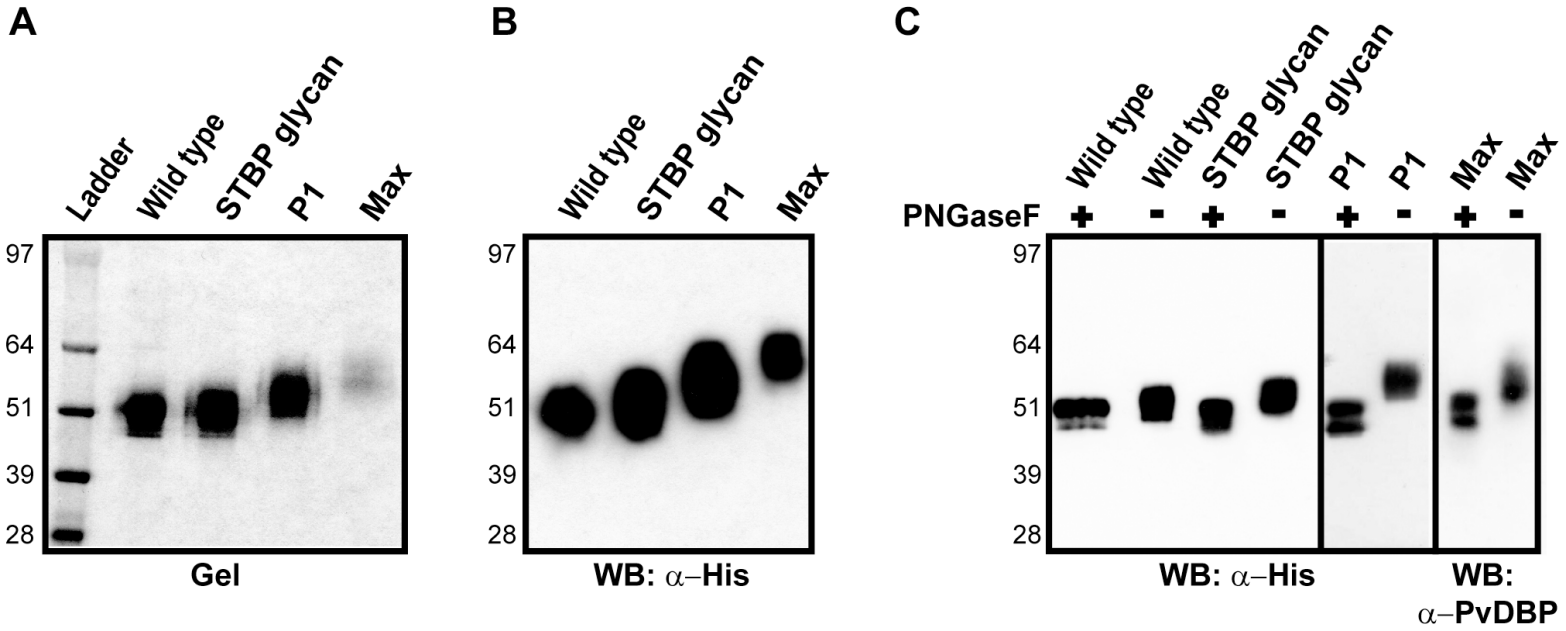
Expression and purification of surface re-engineered PvDBPII recombinant proteins containing additional N-linked glycan residues. (A) 2 µg of PvDBPII wild type and DBPII glycosylation variants were run on SDS-PAGE gel and stained with GelCode Blue reagent. A ladder effect is seen consistent with increasing number of N-glycosylation sites present in the STBP glycan, P1 and Max hyperglycosylated variants compared to PvDBPII wild-type. (B) Western blot of 1 µg of PvDBPII and DBPII glycosylation variants probed with anti-His antibody. (C) PvDBPII wild type and DBPII glycosylation variants were either untreated (−) or digested (+) with N-glycosidase PNGaseF, run on SDS-PAGE gel and probed with anti-His antibody, except for Max variant which was probed with anti-PvDBPII serum. The P1 lanes were run separate from the other samples. Molecular mass is shown on the left.

To produce glycoengineered recombinant proteins, constructs were secreted from the mammalian cell line HEK293F. All of the recombinant proteins were purified to homogeneity, and no truncation products were seen (Figure 3A–B). Each N-glycan adds about 2 kDa to the protein. A ladder effect consistent with increasing N-glycosylation was observed for the recombinant proteins. The wild-type PvDBPII (3 N-glycan sites), STBP glycan and P1 ran at their expected molecular weight, whereas Max migrated slightly lower than the expected fully glycosylated molecular weight of 65 kDa (Figure 3A). Partial glycosylation (underutilization) of some of the 11 N-linked glycosylation sites could account for this. When the proteins were subjected to N-glycosidase treatment, all of DBPII glycosylation variants and the wild-type protein were reduced to a similar size (Figure 3C). These results suggest that many of the introduced sites were appropriately glycosylated in PvDBPII and its N-glycan variants.

### Functional characterization of hyperglycosylated PvDBPII variants by standard in vitro binding assay

**Figure 4:**
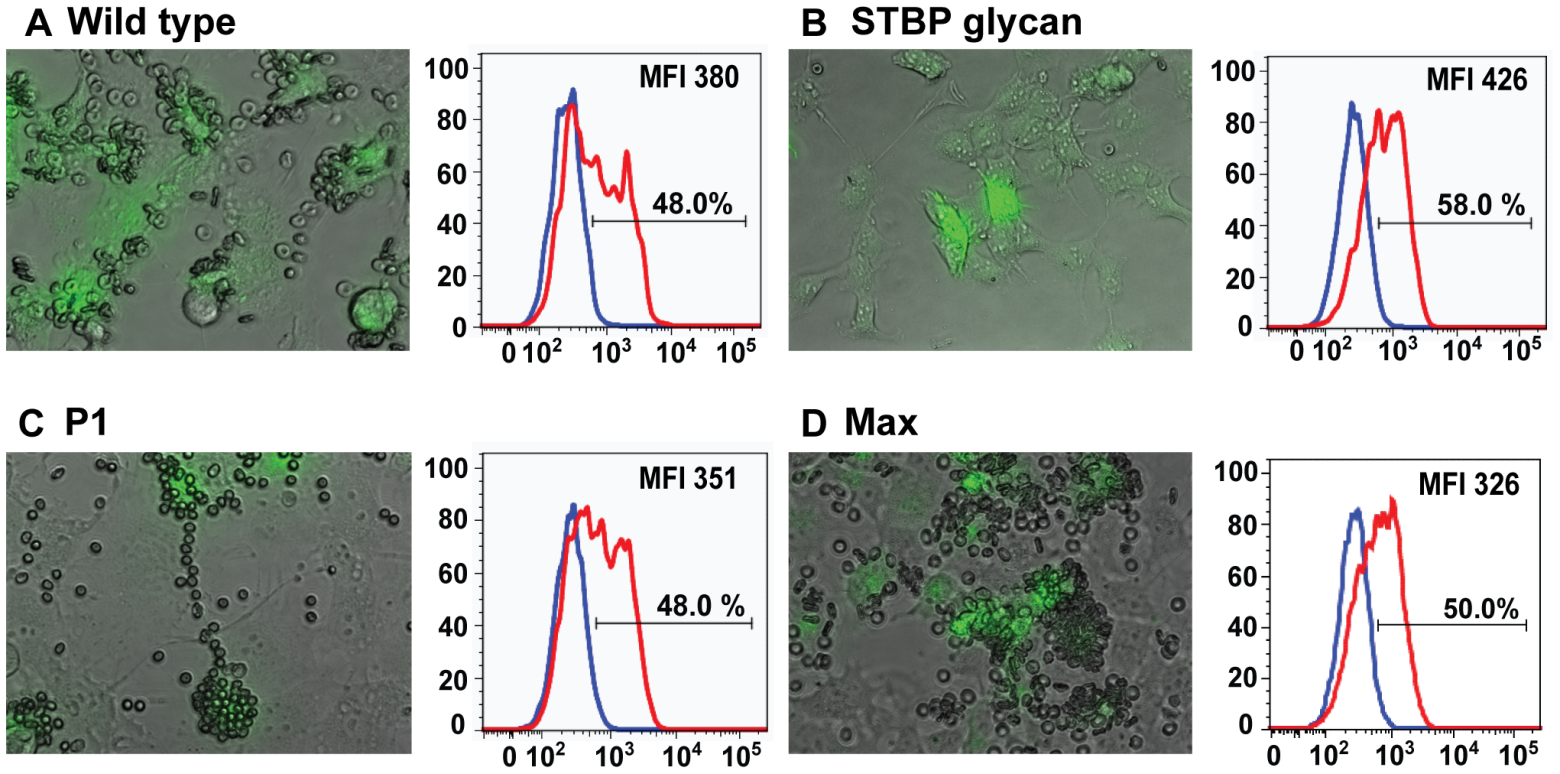
Functional characterization of hyperglycosylated PvDBPII variants by COS-7 cell-RBC binding assay. PvDBPII wild type (A), STBP glycan mutant (B), P1 (C) and Max (D) DBPII glycosylation variants were expressed as a GFP fusion protein in COS-7 cells and incubated with 0.5% hematocrit erythrocytes. GFP-positive transfected cells with five or more RBC (rosettes) were considered positive for binding. The histogram shows the percentage surface labeling when GFP-positive transfected cells were probed with pre-immune (blue) or anti-PvDBPII plasma (red). The gating strategy for GFP-positive and anti-PvDBPII positive is shown in Figure S3. The percentage signifies GFP-positive cells that are labeled with immune plasma, indicating surface expression of PvDBPII and DBPII glycosylation variants. The shift in mean fluorescence intensities between pre-immune and immune plasma is indicated as MFI.

The functional effect of adding N-glycans on PvDBPII was assessed by using an established in vitro PvDBPII cytoadherence assay [12]. In this assay PvDBPII is expressed at the surface of COS-7 cells as a fusion protein with HSVgD1 to aid in surface expression [23]. A GFP reporter is added at the cytoplasmic tail to facilitate identification of transfected cells. Fluorescent cells covered with five or more RBCs, termed rosettes, are considered positive for binding [38]. As expected, nearly all of the cells transfected with wild-type PvDBPII bound to RBCs (Table 1 and Figure 4A). In contrast, addition of the single, extra N-glycan site at N374 nearly completely abolished binding of the STBP glycan mutant (4%) (Table 1 and Figure 4B), while both the P1 and Max variants exhibited binding similar to wild type PvDBP (Figure 4C and D). The reduced binding activity of the STBP glycan mutant was not due to defective surface expression, since it was surface expressed as well or better than the wild-type, P1, or Max glycosylation variants (Figure 4 and Figure S3 for flow cytometry gating strategy). Furthermore, although it was difficult to resolve if there was a difference between STBP glycan and wild-type DBPII in the COS-7 system, P1 and Max migrated slower than wild-type protein prior to N-glycosidase treatment indicating they were hyperglycosylated (Figure S4). Thus, consistent with prior data showing that mutation at I376 strongly reduces DARC binding [17], we find that introduction of the N374 glycan site (I374N/I376T) nearly abolishes DARC binding, either as a consequence of the mutation or addition of N-glycosylation. In addition, the P1 and Max mutants were functionally folded, despite additional surface glycoengineering.

A meta-analysis of previous PvDBP-DARC binding studies and the new glycan mutants was performed. The majority of previous site-directed mutagenesis was concentrated in subdomains 1 and 2 (Table S1). Fewer residues had been mutated in subdomain 3 and many of these were buried residues (Table S1). In this study, a total of two, three and seven surface residues were modified in subdomains 1, 2, and 3 with no effect on DARC binding. Overall, critical binding residues (30% or more loss of binding) are concentrated to the predicted binding sites in subdomains 1 and 2 (model 1) or at the dimer interface (model 2), suggesting both areas are critical for DARC binding. Conversely, no surface changes in the highly conserved subdomain 3 have affected DARC binding (Figure 1G, H and Table S1).

### Immunogenic characterization of PvDBPII and its hyperglycosylated variants

**Figure 5:**
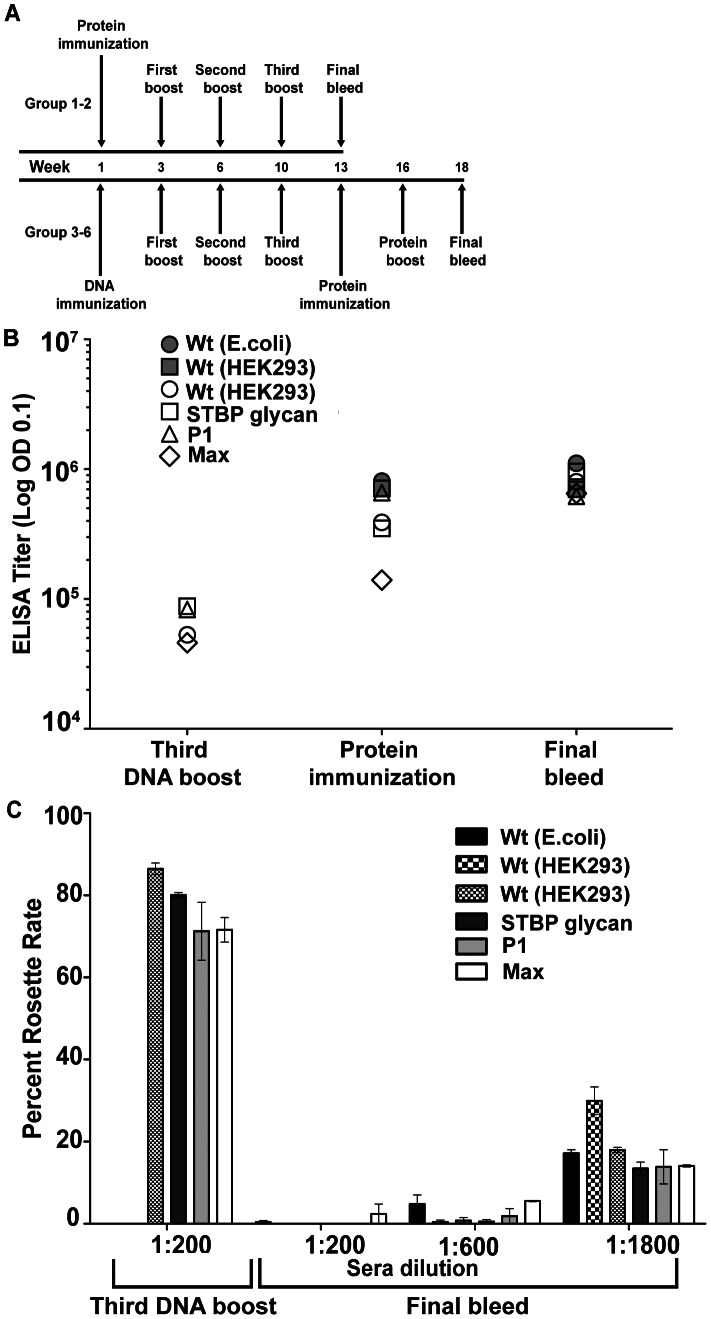
Immunogenic characterization of PvDBPII and its hyperglycosylated variants. (A) Schematic representation of immunization scheme for PvDBPII wild type and DBPII glycosylation variants. Group 1 mice received PvDBPII wild type generated in *E. coli*, Group 2 mice received PvDBPII wild type generated in HEK-293F cells. Mice in groups 3–6 received a combination of DNA and homologous protein; group 3, PvDBPII wildtype; group 4, STBP glycan; group 5, P1; group 6, Max hyperglycosylated variant. (B) The ELISA end-point antibody titers are shown for each of the immunization groups after the third DNA boost, the first protein immunization, and the second protein boost (final bleed). (C) The inhibitory activity of vaccine plasma in a COS-7-RBC binding assay. COS-7 cells expressing PvDBPII wild type protein were pre-incubated with varying dilutions of PvDBPII wild type and DBPII glycosylation variant immune plasma obtained after the third DNA boost or the second protein immunization (final bleed). The values are an average of duplicate experiments.

To investigate immunogenicity, mice were immunized with wild-type and DBPII glycosylation variants. Since the yield of PvDBP recombinant proteins was low (between 0.1 to 0.5 mg/L) we employed a DNA prime–protein boost strategy (Figure 5A). In this approach, mice were immunized by DNA electroporation with plasmid DNA and boosted with the matching DBPII wild-type or DBPII glycosylated variant protein produced in HEK293F cells (Figure 5A). As a control, mice were immunized with wild-type protein alone that was produced in *Escherichia coli* (no N-glycosylation) or HEK293 cells (3 native N-glycosylation sites).

Antibody titers were compared using the *E. coli* recombinant protein that lacks N-glycosylation and therefore has no potential antibody epitopes hidden. One-way analysis of variance (ANOVA) of individual mouse ELISA titers showed no significant difference in antibody titer between any of the six immunization groups (Table S2) at the time of the final bleed (Figure 5B). This indicated that there was no loss of immunogenicity of glycoengineered recombinant proteins (groups 4–6), despite having significant surface glycosylation (Figure 1). Furthermore, mice that received four protein immunizations with 5 µg of protein per immunization (groups 1 and 2) had similar final antibody titers to mice that received four DNA immunizations followed by two protein boosts with 2.5 µg of protein per immunization (group 3). Thus, DNA electroporation–protein boost offers a strategy to circumvent issues that arise due to limited availability of recombinant PvDBPII protein. However, DNA electroporation alone was insufficient to elicit strong adhesion blocking antibody responses. The antibody titer increased by more than an order of magnitude after the two protein boosts (Figure 5B) and there was almost no inhibitory activity following the fourth DNA immunization (Figure 5C). Conversely, plasma obtained after the two protein boosts exhibited considerable inhibitory activity even at 1∶1800 dilution in a standard COS-7 cell-red blood cell (RBC) binding inhibition assay [23] (Figure 5C).

### Inhibition of PvDBPII binding to DARC on erythrocytes

**Figure 6:**
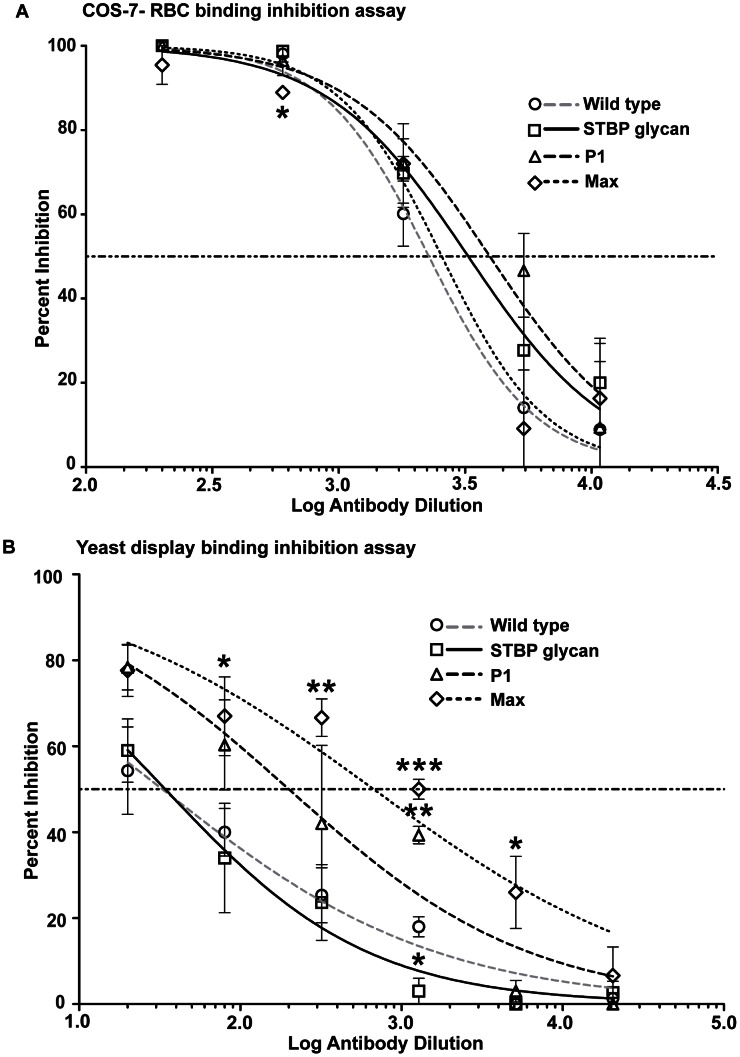
Inhibition of PvDBPII binding to DARC in different assay platforms. (A) Inhibition of PvDBPII – DARC interaction by immune plasma in the COS-7 cell–RBC binding inhibition assay. COS-7 cells expressing wild-type PvDBPII were incubated with varying dilutions of immune plasma obtained after the final protein boost. The values are an average of two experiments. (B) Inhibition of PvDBPII – DARC interaction by immune plasma in the yeast display binding inhibition assay. Yeast cells expressing wild-type PvDBPII on the surface were incubated with varying dilutions of immune plasma obtained after final protein boost and then probed with recombinant DARC-Fc. Values are an average of three experiments, standard deviation is shown as error bars. Student's t test, p value<0.001 is indicated by ***, p value from 0.001 to 0.01 is shown by ** and 0.01 to 0.05 by *.

Plasma from the different immunization groups were evaluated for inhibitory activity using the COS-7 cell–RBC binding assay. The inhibitory assay was carried out for plasma dilutions ranging from 1∶200 to 1∶10800 (Figure 6A and S4). A 1∶200 dilution of pre-immune plasma showed no inhibition of RBC binding (rosette formation) and was used as control. A statistical comparison of the Least Squared Distance of data points shows that the non-linear model provides a good fit. Thus, the percent inhibition of rosette formation was plotted against log plasma dilution and analyzed by non-linear regression to determine the IC50. The IC50 values of the wild-type *E. coli* and HEK293 DBPII proteins were similar (Figure S5). To investigate the effect of glycan masking the IC50 values of wild-type PvDBPII and the three DBPII glycosylation variants in the DNA-protein prime boost regimen were compared. Individual values ranged from 1∶2100 to 1∶2800 and were not statistically different in the non-linear model (Figure 6A, Table S3). This result shows that DBPII glycosylation variants elicited similar levels of inhibitory antibodies to wild-type immunogen in the COS-7-RBC binding format, despite having significantly greater surface glycosylation.

### Inhibition of PvDBPII binding to recombinant DARC

To further investigate whether any of the glycosylated immunogens provided a small improvement in inhibitory antibodies, we developed a novel flow cytometry based antibody-inhibition assay that is easier to quantify and more amenable to high-throughput analysis than the COS-7-RBC binding assay. In this assay, wild-type PvDBPII, minus the 3 native N-glycan sites, is expressed by yeast surface display [39] and probed with recombinant DARC-Fc. Binding of DARC-Fc is detected using an antibody to the Fc fusion domain. In control experiments, the wild-type YY-DARC-Fc recombinant protein bound to transfected yeast cells, while the YF-DARC-Fc variant lacking the critical sulfotyrosine site at position 41 [15] did not bind (Figure S6). The percent inhibition of wild-type DARC-Fc binding was then plotted against log plasma dilutions from the four immunization groups. In this assay, plasma from the STBP glycan variant had slightly weaker inhibitory activity than the wild-type PvDBP immunogen (Figure 6B), while P1 and Max elicited stronger inhibitory activity. The IC50 values for P1 and Max were 7-fold and 20-fold lower than for wild-type immunogen (Figure 6B, Table S3). This difference was statistically significant (p = 0.02 for both P1 and Max, one tailed Student's *t* test). Moreover, the percent inhibition by P1 or Max plasma was significantly better than wild-type at multiple plasma dilutions (Figure 6B). This analysis suggests that an inhibitory epitope in PvDBPII was eliminated in the STBP glycan immunogen, whereas increased levels of DARC-binding-inhibitory antibodies were elicited by P1 and Max DBPII glycosylation variants.

### Effect of the FyA/B DARC allele on binding inhibition

**Figure 7:**
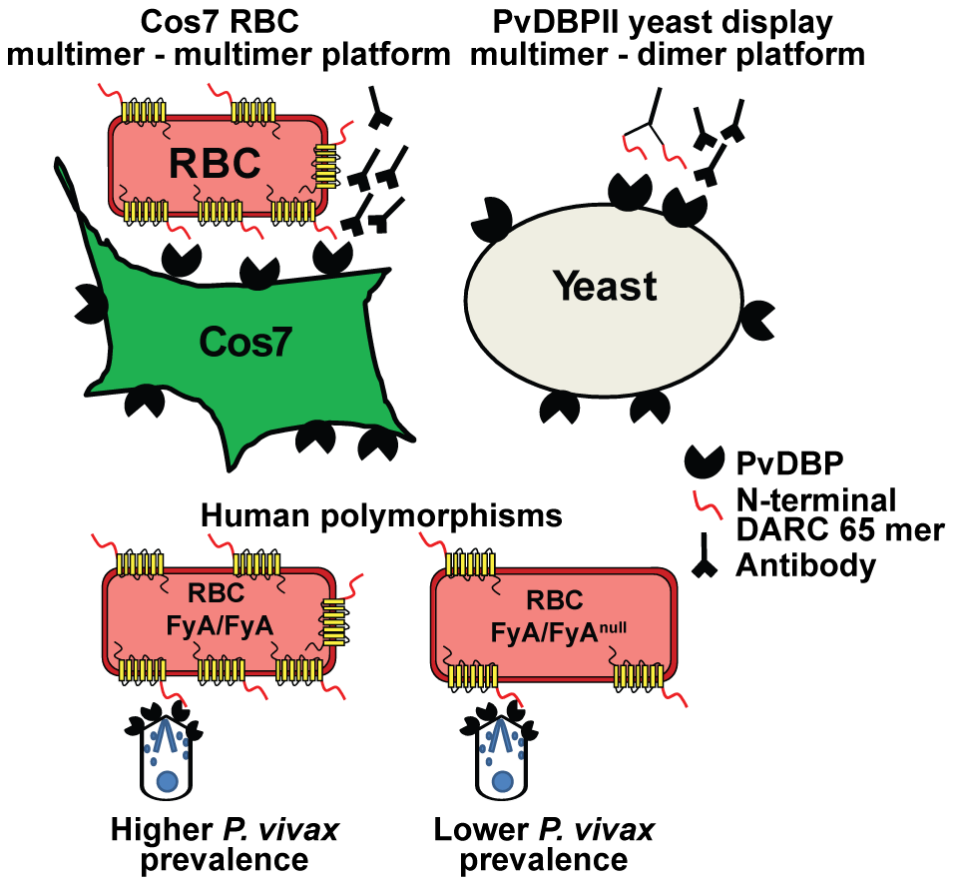
PvDBP – DARC interaction. DARC, a seven transmembrane chemokine receptor on erythrocytes, has been shown to bind to PvDBP [16]. Binding has been mapped to the N-terminal 65 amino acids (illustrated as a red tail). The COS-7-RBC cytoadherance assay is based upon a multivalent interaction between PvDBPII present on surface of COS-7 cells and DARC expressed by RBC. In the yeast-PvDBPII display assay, PvDBPII present on the yeast surface interacts with dimeric recombinant DARC-Fc recombinant protein (N-terminal 65 mer region). The two assays offer different platforms to reveal inhibitory effects of antibodies using a potentially higher affinity, multimer-multimer interaction (COS-7 format) or a lower affinity multimer-dimer interaction (yeast display). Bottom of figure, polymorphisms in DARC that generate FyA/FyA^null^ genotype are associated with half of the number of surface DARC protein and lower *P. vivax* infection [4].

**Figure 8:**
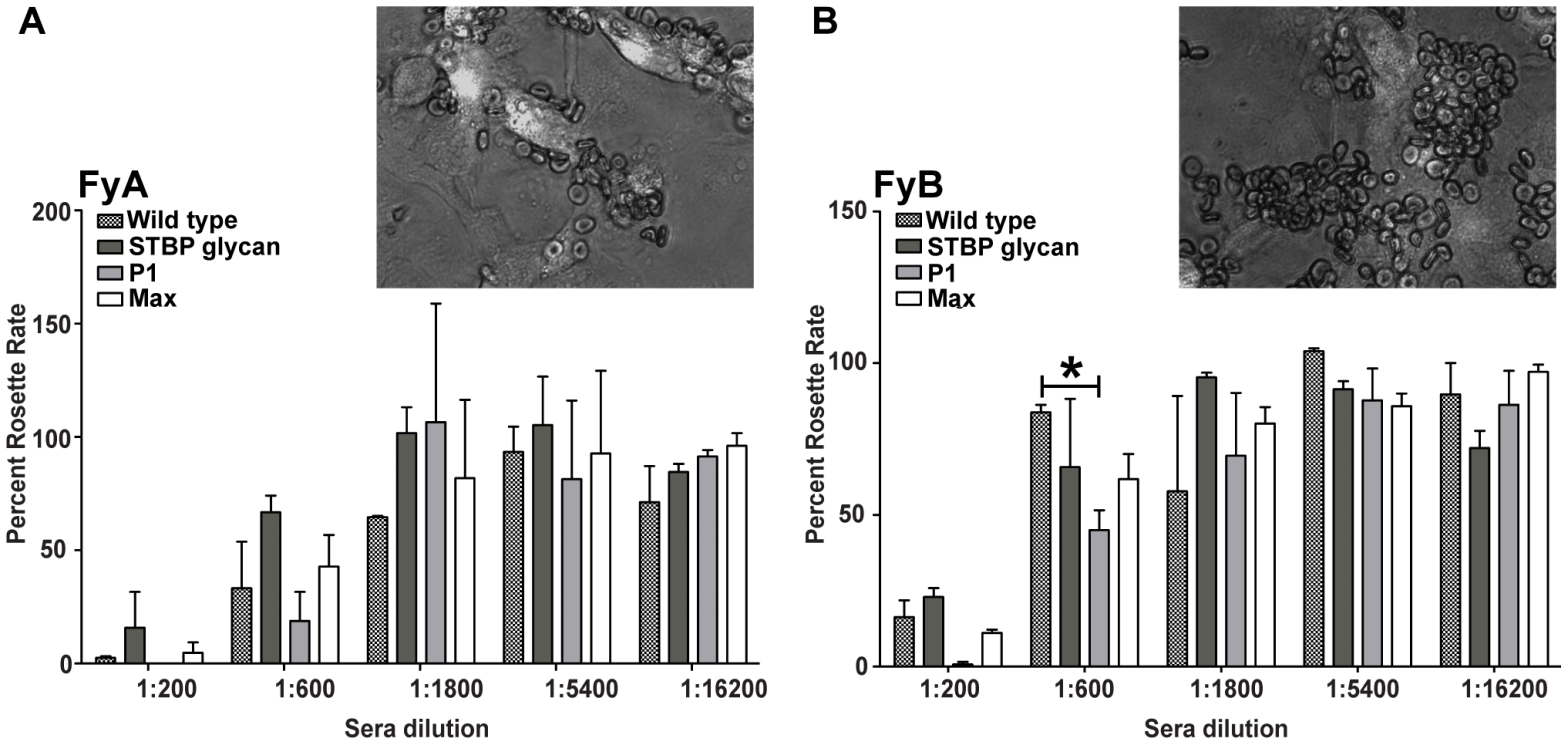
Effect of DARC phenotype on antibody binding inhibitory activity. COS-7 cells expressing PvDBPII as a GFP fusion protein were incubated with immune plasma and then RBC expressing the FyA (A) or FyB (B) Duffy blood group antigen were added. The inset shows that FyA RBCs (A) gave smaller rosettes than FyB RBCs (B) in the COS-7 cell–RBC binding assay. Statistical testing and p values as explained in Figure 6B.

A potential explanation for the discrepancy in antibody inhibition results between COS-7 and yeast display formats is that the yeast assay may be more sensitive to detect differences in antibody inhibitory activity. In the yeast assay, antibodies are challenged to inhibit an interaction of limited valency (the binding of DARC-Fc to yeast-displayed-PvDBPII is mono- or bi-valent) while in the COS-7 assay, antibodies are challenged to inhibit an interaction between two cells (RBCs displaying DARC and COS-7 cells displaying PvDBPII) that is likely to be multi-valent and potentially highly multi-valent (Figure 7). We initially conducted our assays using an FyB RBC donor (COS-7) or an FyB DARC recombinant protein (yeast) [15]. However, the FyA allele is more common in *P. vivax* endemic regions, and recent work suggests it has lower PvDBP binding activity and greater susceptibility to antibody blockade than FyB [8]. To investigate if the immune plasma would have greater activity on FyA donors in the COS-7 format, we screened five FyA/FyA donors and one FyB/FyB donor using the COS-7 cell–RBC binding assay. Surprisingly, only two of the five FyA donors exhibited reduced binding activity. This was mostly manifest as smaller RBC rosettes (Figure 8 inset). The COS-7 cell–RBC antibody inhibition assay was then repeated with RBCs from the weakest binding FyA donor and an FyB donor (Figure 8). Notably, a similar percentage of transfected COS-7 cells bound FyA and FyB RBCs. However, FyA RBCs formed smaller rosettes and were more susceptible to antibody inhibition from each of the four immunization groups than FyB cells (Figure 8). P1 plasma had measurably higher inhibitory activity than wild-type plasma at 1∶200 and 1∶600 dilutions against FyA and FyB RBCs, but this trend only achieved significance for one dilution value in the FyB experiments and was not apparent at higher dilutions. No equivalently significant trends were observable for STBP or Max plasma.

## Discussion

Efforts to make a blood stage malaria vaccine have proven extremely challenging because of antigen polymorphism, redundant pathways of RBC invasion, and the rapid kinetics of parasite invasion [40]. Although *P. vivax* is highly restricted to reticulocytes and theoretically poses fewer challenges for blood stage vaccine development than *P. falciparum*, PvDBP is polymorphic [28], [29] and vaccine-induced antibodies are only partially effective at inhibiting the PvDBP-DARC binding interaction [21], [24], [41]–[44]. Therefore, new immunogen design strategies and adjuvants [45] are required to enhance neutralizing antibody responses and confer cross-strain protection.

Glycan masking is an emerging vaccine design strategy to target antibodies to specific antibody epitopes. A glycan shield is employed by HIV and other enveloped viruses, such as influenza, to hide regions of viral surface proteins from neutralizing antibodies [46]–[48]. N-linked glycans are bulky, flexible structures, more than 20 times the size of an amino acid side-chain. Carbohydrates are poorly immunogenic, and the large carbohydrate unit effectively shields the underlying amino acids from antibody recognition [49]–[52]. Although some HIV-broadly-neutralizing antibodies isolated from natural infection utilize glycans as part or all of their epitope [39], [53]–[56], the basis for glycan-targeting in these cases may be due to the extraordinarily high density of glycans on HIV Envelope (which may create “non-self” epitopes from “self” glycan molecules) and/or to the fact that HIV infection causes significant dis-regulation of the immune system or other factors. Indeed, glycan reactive monoclonal antibodies display extensive somatic hypermutation and/or unusually long complementarity determining regions [57], [58], suggesting that their elicitation may require highly persistent infection. In the context of vaccine design, glycan masking has mostly been evaluated on HIV Envelope glycoproteins. Glycan masking has reduced monoclonal antibody recognition of targeted epitopes on HIV Envelope gp120/gp140, but it has not led to broader neutralizing antibody responses [32]–[34]. However, gp140 contains 30 potential N-glycosylation sites on average, and most of its surface is predicted to be covered by N-glycans [59]. In contrast, malaria encodes a minimal N-glycosylation machinery and malaria surface proteins may contain limited or no N-glycosylation. *P. falciparum* encodes an identifiable Alg7 glycosyltransferase and SST3 oligosaccharyltransferase, and it is predicted that only a short GlcNAc_2_ group would be transferred to proteins [36]. Thus, *Plasmodium* proteins may be better platforms to evaluate this vaccine design strategy because they are not already highly evolved to evade antibody responses via glycan masking.

Here, glycan masking was used both to probe PvDBP binding function and to test a vaccine strategy to focus antibodies on critical interaction surfaces. Although several different PvDBP-DARC binding mutagenesis studies have been done, there is still controversy surrounding the PvDBPII-DARC binding model [13], [14]. The first mutagenesis studies were performed before the PvDBP structure was solved [17], [18] and there has been limited structure-guided mutagenesis analysis. The present study shows that elements of both the “just in time” and “receptor-mediated dimerization” models are correct. We found that an N-glycan site added to a predicted sulfotryosine binding pocket in subdomain 2 abolished DARC binding, and that N-glycan sites added distant from this interaction site or the dimer interface had no effect on DARC binding. These findings confirm the importance of the subdomain 2 interaction site for DARC binding, as predicted in the “just in time” model, and extend our understanding of the DARC “binding footprint”. The meta-analysis also suggests that residues critical for DARC binding [13], [17]–[19] are localized to the dimer interface (as predicted by the “receptor-mediated dimerization” model) and at a narrow patch on subdomain 2 and wrapping around to adjacent regions in subdomain 1 (consistent with the “just in time” model). It is possible that the DARC interface encompasses both regions or that the DARC binding site and dimer interface are separate and distinct, but both may contribute to efficient DARC binding. There are still many surface residues in close proximity to the predicted DARC interface in model 1 that need to be evaluated for binding, including the adjacent subdomain 3.

In mouse immunization studies, glycoengineered proteins were equally immunogenic to wild-type proteins by ELISA. This finding is encouraging because it suggests that substantial glycoengineering did not inadvertently blunt T helper cell priming [60], [61] or the development of anti-PvDBP antibody responses. However, differentially glycosylated PvDBP variants failed to yield significant differences in antibody inhibitory activity in the COS-7-RBC binding assay. While the response of P1 plasma at 1∶200 and 1∶600 dilutions hints at improved response against the P1 variant, the difference in response was insufficient to allow proper determination of a significant difference in elicited IC50.

In contrast, consistent and significant enhancement of PvDBP∶DARC binding inhibition was observed in the yeast display assay used in this study. Of particular interest is the observed increase in inhibitory potency elicited by the P1 and Max constructs with increased glycosylation distal to predicted inhibitory antibody binding areas, as well as correspondingly decreased inhibition elicited by the STBP construct. The extra N-glycosylation site in the STBP construct covers a linear inhibitory antibody epitope from natural *P. vivax* infections [20], adding plausibility to the interpretation that inhibitory antibodies map near this region and can be targeted by vaccination. The yeast display assay provides greater ease and throughput and may be more sensitive to inhibitory antibody responses due to the lower PvDBP-DARC binding valency in the yeast format (Figure 7). This sensitivity to detect small differences in antibody activity may be important in engineering more potent PvDBP immunogens in the future. Indeed, small 2-fold differences in DARC surface levels [4] or FyA allelic polymorphism [8], are thought to affect PvDBP binding activity and the ability of anti-PvDBP antibodies to block parasite invasion (model in Figure 7). Thus, this interaction may be particularly vulnerable to antibody blockade.

If the P1 and Max variants successfully enhanced inhibitory binding titers as implied by the yeast display assay, critical questions for further study are whether the increased potency observed in the yeast assay is indeed clinically relevant, and whether the glycosylated constructs developed in this study are amenable to further engineering and concomitant improvement in elicited immune response. One possibility is that in designing the current constructs we may have inadvertently masked inhibitory epitopes that have recently been localized to subdomain 3 [24] and are only partially mapped [20] (Figure 2). In addition, it remains possible that the more pronounced activity of Max plasma in the yeast assay compared to the COS-7 platform may be a consequence of the fact that the PvDBP constructs in the COS-7 and yeast display assays were smaller than the PvDBP vaccine immunogen and slightly different from each other to facilitate protein expression (Figure S1). In particular, the N-terminus of the yeast display construct was 8 amino acids shorter than the COS-7 construct and 18 amino acids longer at the C-terminus. Thus, potentially inhibitory antibodies directed against the C-termini of PvDBP would be less likely to be detected in the COS-7 assay than the yeast assay. As more inhibitory epitopes are mapped in PvDBP, it may be possible to improve glycoengineered PvDBPII immunogens by repositioning N-glycosylation sites to shield off-target epitopes and focus antibodies on more strain-conserved inhibitory epitopes. Whereas this study used a DNA and protein prime-boost approach, glycoengineering could also be applied with viral vectored vaccines, or with protein-only immunization if a strategy for improved PvDBP expression could be developed.

In conclusion, this analysis shows that PvDBPII recombinant proteins retain immunogenicity despite considerable glycoengineering and that glycan resurfacing offers an integrated approach to characterize protein function and immunogenicity. Glycan resurfacing of PvDBPII immunogens may have utility in *P. vivax*-malaria vaccine development.

## Materials and Methods

### Cloning of PvDBPII in pTT3 vector

PvDBPII and PvDBPII glycosylated variants were synthesized at Genscript with mammalian codon optimization and cloned into a modified version of the pTT3 vector (kindly provided by Dr. Yves Durocher, Biotechnology Research Institute, National Research Council Canada) using HindIII and BamHI restriction sites. The PvDBPII is in frame with a signal sequence from murine IgG kappa chain at the N-terminus and 6× His tag at the C-terminus [62]. Sequences of wild-type PvDBPII (Salvador 1 strain, Sal1) and glycosylated variants are provided in Figure S1.

### Design of glycosylated variants

N-linked glycosylation site selection for PvDBPII (Sal1 variant) was performed by fixed-backbone redesign in Rosetta, using Rosetta-generated homology models derived from the crystal structure of the *P. knowlesi* PkDBPII (2C6J) [14], [63], [64]. The PvDBPII sequence was checked for positions where one mutation adds an N-glycan consensus sequence N-X-(S/T) where X is any amino acid except for proline [65]. Putative glycan sites were screened for solvent accessibility of the glycan-bearing asparagine residue; sites were mostly spaced no closer than 5 amino acids to an existing glycosylation site based on prior work showing decreased efficiency for closely spaced sites [66]. Steric clashes due to N-glycans being in proximity were addressed by using the Rosetta program. During site selection, consideration was also given to secondary structure preferences for N-glycosylation sites [37], and to avoid the introduction of buried unsatisfied polar groups at the S/T position.

### Expression and purification of PvDBPII and PvDBPII glycosylated variants

HEK-293F cells were cultured in SFM4TRNSFX media (Hyclone). For expression and purification, 400 million cells were suspended in 20 ml of fresh SFM4TRNSFX media. Cells were transfected with 500 µg of DNA and 1 mg of PEI MAX (Polysciences.com) using a previously described procedure [67]. This suspension was shaken at 125 rpm and incubated at 37°C and 5% CO_2_ for 4 h, then made up to 400 ml with Freestyle media (Invitrogen) and placed back in the shaker. Tissue culture supernatant was harvested 5 days after transfection and spun at 1000 rpm for 20 min to remove cell debris. Following centrifugation, the supernatant was balanced with 20 mM HEPES pH 7 and 200 mM sodium chloride. Recombinant PvDBPII proteins were affinity purified using Nickel Sepharose 6 Fast Flow resin (GE Healthcare), dialyzed into 1× PBS and stored. To assess purity, recombinant PvDBPII and glycosylated variants were run on 4 to 20% SDS gel (Invitrogen) and stained with GelCode Blue solution (Invitrogen) or transferred to PVDF membranes and probed with anti-His antibody or with rabbit anti-PvDBP plasma for Western blots.

### Ethics statement

This study was carried out in strict accordance with the recommendations in the Guide for the Care and Use of Laboratory Animals of the National Institutes of Health. Rabbit immunizations were performed by custom vendor at R&R Rabbitry. Mice immunizations were performed by Seattle Biomedical Research Institute personnel. Animals were housed under controlled laboratory conditions. The protocols were approved by the Institute Animal and Care Use Committee at Seattle Biomedical Research Institute (Protocols JS-ABP and JS-04).

### Rabbit and mice immunizations

For rabbit immunizations, four rabbits were immunized with *E. coli* generated refolded PvDBPII protein at R&R Rabbitry in accordance with current guidelines on animal immunizations. Two animals received 50 µg of protein in Freund's complete adjuvant for the first injection followed by protein in incomplete Freund's adjuvant for the subsequent 5 boosts. Two others received 50 µg of protein in TiterMax gold adjuvant (TiterMax.com) for the immunization and the boosts. Boosts were carried out every 3 weeks. For mice immunizations, 6 groups of 10 mice each were subjected to immunization according to animal immunization guidelines. Group 1 and 2 mice received protein only for the immunization and three boosts. Mice in groups 3 to 6 received DNA immunization followed by three DNA boosts and two protein boosts at three week intervals. Protein immunizations were given as intramuscular injection into the left tibialis muscle. DNA immunization was done by DNA electroporation in the anterior tibialis muscle using Trigrid delivery system (ICHOR). Group 1 mice received 10 µg of *E. coli* generated, refolded PvDBPII. Group 2 mice received 5 µg of PvDBPII generated in HEK-293F cells. Groups 3–6 mice received 20 µg of DNA and 2.5 µg of PvDBPII, PvDBPII STBP glycan, PvDBPII partial glycosylated variant (P1) and PvDBPII maximum glycosylated variant (Max), respectively (Table S2). A combination of CpG Oligo dinucleotide (ODN1826, Invitrogen) and alum was used as the adjuvant for protein immunizations [68].

### ELISA

100 ng of recombinant PvDBPII produced in *E. coli*, refolded, and lacking in glycosylation was coated on 96 well plates (Nunc) by overnight incubation at 4°C. Mice plasma dilutions ranging from 1∶500 to 1∶512,000 were added to the plate and probed with ELISA kit (Alpha Diagnostic International Inc.) according to manufacturer's protocol. The results were analyzed using SoftMaxpro 5.0, graph fitted to 4-pt fit curve and OD determined at 0.1.

### Flow cytometry analysis of wild-type and glycoengineered PvDBPII

COS-7 cells were grown in 60 mm culture dishes containing one coverslip each. Cells were transfected using GeneJuice transfection reagent and 1 µg plasmid DNA of wild-type PvDBPII-GFP fusion (pEGFP-DBPII, kindly provided by Dr. John Adams, University of South Florida) or PvDBPII glycosylated variants (construct boundaries, DHKK…EVVT) (Figure S1) in pEGFP-N1 vector [23]. 48 h after transfection, the coverslips were transferred to a fresh culture dish for performing a COS-7-RBC binding assay described below. The remaining cells in the culture dish were lifted with 8% EDTA and washed with 1× PBS (0.1% BSA). The cells were then stained with rabbit anti-PvDBPII plasma for 1 h at 4°C, followed by washing and labeling with goat anti-rabbit Texas Red (Southern Biotech) for 30 min. Antibody-labeled cells were washed and resuspended in PBS and analyzed by LSRII (Becton Dickinson). The data was analyzed by FlowJO 8.1 software (Tree Star Inc.)

### COS-7 cells-RBC binding assay

To the coverslips generated above, 0.5% hematocrit blood was added and left for 1 h in a humidified chamber at 37°C. The culture dish was then filled with complete DMEM, a glass stand was placed in the dish, and the submerged coverslip was flipped on the stand for 10 min to allow unbound RBC to settle by gravity. Coverslips were then washed with 1× PBS and fixed with 1% glutaraldehyde for 20 min. The coverslips were moved into 1× PBS and 10 fields were counted at 200× magnification using a fluroscence microscope. Transfected cells bearing 5 or more RBCs were counted as rosettes.

### COS-7 cells-RBC antibody binding inhibition assay

COS-7 cells were cultured and transfected as described above. Coverslips were incubated with varying dilutions of final bleed mouse plasma or a 1∶200 dilution of pre-bleed mouse plasma in incomplete DMEM for 1 h at 37°C in a humidified chamber [23]. Coverslips were washed in 1× PBS and layered with 0.5% hematocrit blood for 1 h in a humidified chamber at 37°C. The cells were then washed, fixed, and counted, as described above. Percent inhibition was calculated at each plasma dilution as the percent decrease in RBC rosette count with plasma incubation relative to the rosette count without plasma using the formula [(percent rosette rate for no plasma - percent rosette rate for plasma dilution)/percent rosette rate for no plasma]×100. For each immunization group, an inhibition curve (percent inhibition versus plasma dilution) was generated using GraphPad Prism version 5.02 (GraphPad Software) and the plasma dilution at which 50% inhibition was recorded (IC50) was determined by transforming the data to a log10 scale with fitted sigmoidal dose-response curves.

### Yeast display antibody binding inhibition assay of PvDBPII and DARC-Fc

Yeast display of PvDBPII constructs was performed as per [39], using PvDBPII construct boundaries corresponding to the 2C6J PkDBPII crystallization construct [14]. In brief, a PvDBPII insert (amino acids, AIIN…PISQ) (Figure S1) was expressed as an Aga2p fusion protein at the yeast cell surface under the control of a galactose-inducible promoter in the episomally maintained yeast display plasmid pCTCON2. A linear PvDBPII insert with flanking regions homologous to pCTCON2 was designed using DNAWorks and synthesized by PCR assembly of oligonucleotides [69]. Nucleotides were codon-optimized for *S. cerevisiae* and all asparagines in potential N-linked glycosylation sites were mutated to glutamines. Viable plasmid was generated by homologous recombination following electroporation into EBY100 yeast as reported by Chao et al. [39]. Yeast clones were isolated by serial dilution on selective media and sequenced using the Zymo Research Yeast Plasmid Miniprep II kit. A yeast colony carrying the desired PvDBPII sequence was used for all further work. Induction of the Aga2p-PvDBPII fusion protein from the pCTCON2 vector and passaging of the transformed yeast were performed as described [39].

Binding and flow cytometry assays were modeled on the primary-secondary antibody protocol of Chao et al. [39]. In brief, 80,000 induced yeast per well were deposited in a 96-well plate, centrifuged for 5 minutes at 700 g, washed with PBSF (PBS+0.1% bovine serum albumin) and incubated for 10 minutes with serial dilutions of immune plasma. The yeast cells were then washed with PBSF again and incubated with a 1∶100 dilution of DARC-Fc (10 mg/L stock) in PBSF. After 10 mins incubation, the cells were washed with PBSF again and binding of the DARC-Fc protein was detected with a 1∶250 dilution of phycoerythrin-conjugated goat anti-human polyclonal antibody preadsorbed against mouse and rabbit antibodies. DARC-Fc and DARC-Fc Y43F inactive mutant [17] were used as positive and negative controls respectively in wells without immune plasma incubations. Antibody-labeled cells were washed and resuspended in PBS and analyzed by LSRII (Becton Dickinson). The data was analyzed by FlowJO 8.1 software (Tree Star Inc.). To calculate plasma inhibition, the population of PvDBPII expressing yeast was first determined by comparing uninduced to induced yeast after incubation with DARC-Fc and secondary antibody. The percent inhibition at each plasma dilution was then calculated as the percentage reduction in the shifted yeast population between the immune and pre-immune plasma. An inhibition curve and IC50 values were generated using GraphPad Prism as described above.

### PCR-RFLP analysis to determine the DARC promoter type and FyA or FyB allele

The following protocol was adapted from [4]. Genomic DNA was extracted from 10 mls of whole blood using the QIAamp blood extraction kit following the manufacturer's directions (Qiagen). PCR was performed in 50 µl reactions containing 200 nM of the appropriate positive-strand and negative-strand primer (IDT DNA technologies); 200 µM each dNTP (New England Biolabs); 1.25 units of Taq DNA polymerase (25 and 50 µL reactions respectively; NEB); 1× polymerase buffer (NEB) and at least 200 ng of purified human genomic DNA. Two amplicons were used to characterize FY polymorphisms. The promoter fragment was characterized with (FYPup 5′-CTTCGGTAAAATCT CTACTTGCTGGAAAGC-3′ and FYPdn 5′-CCATGGCACCGTTTGGTTCAGG-3′) and the coding sequence with (FYup 5′-GACTCTTCCGGTGTAACTCTGATG-3′ and FY851[-] 5′GGCCAAGACGGGCACCACAATG-3′). The thermocycling program used for both amplifications was 30 seconds at 95°C, 30 seconds at 58°C, and 30 seconds at 68°C for 30 to 45 cycles. The promoter-specific fragment was digested with StyI, and the coding-specific fragment digested with BanI for 2 h at 37°C. Restriction fragments were visualized after electrophoresis on 4% agarose (Lonza) gels stain. BanI digestion of the coding-specific PCR produces segments of 212 and 151 bp (FY*A allele) and/or 363 bp (Fy*B allele). StyI digestion of the Fy promoter-specific fragment produces segments of 144, 108, and 77 bp (erythrocyte active promoter) or 144, 108, 65 bp (erythrocyte silent promoter).

### Entropy calculations

The amino acid variation at single multiple alignment position of a line-up of PvDBP sequences (Figure S2) was calculated using the formula:

Fractional Shannon entropy values 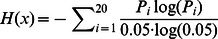
where *P_i_* represents the observed frequency of a residue type *i* in the aligned column [70]; the probability of a particular residue occurring at a given position is thus 0.05 ( = 1/20) for a uniform amino acid distribution, and the choice of logarithmic base is rendered irrelevant by the normalization process. The minimum positional entropy (0) occurs at perfectly conserved positions and the maximum positional entropy (4.32 = log_2_ 20) occurs at positions where all amino acids are observed with equal frequency.

## Supporting Information

Figure S1 **Sequence of PvDBPII wild type and glycoengineered variants.** N-glycan sites are highlighted in yellow. The PvDBP sequence indicates the amino acid boundaries for the DNA and protein immunization constructs. The PvDBP construct boundaries for COS-7 assay are underlined and those for yeast display are shown in red.
(PDF)

Figure S2 **Alignment of PvDBP sequences.** A multiple alignment was generated by Clustal 2.1 from 129 PvDBP sequences [28], [29] and used to generate the entropy scores at each amino acid position in the line-up. *, fully conserved residue; : strong conservation; . weak conservation.
(PDF)

Figure S3 **Transfection of COS-7 cells with PvDBPII-GFP fusion proteins.** The gating strategy for GFP and anti-PvDBPII doubly positive transfected COS-7 cells is shown. The gate for GFP-positive cells was set by comparison to untransfected cells. The gate for anti-PvDBPII-positive (PE-Texas Red) was set by comparison to cells labeled with secondary antibody alone.
(PDF)

Figure S4 **Western blot of DBPII glycosylation variants expressed in COS-7 cells with or without PNGaseF treatment.** COS-7 cells transfected with recombinant DBPII glycosylation variants were lysed 48 h post transfection and immunoprecipitated with anti-GFP agarose resin. Half of the sample was subjected to PNGaseF treatment and the other half was untreated to observe glycosylation modifications.
(PDF)

Figure S5 **Inhibition of PvDBPII binding to DARC in different assay formats.** Mice were immunized with wild-type DBPII protein produced in *E. coli* or HEK293 cells. Antibody inhibition of PvDBPII-DARC interaction in COS-7-RBC binding inhibition assay (A) and yeast display binding inhibition assay format (B).
(PDF)

Figure S6 **Yeast display antibody binding inhibition assay.** Histograms showing DARC-Fc binding to yeast. (A) Uninduced yeast with no PvDBPII surface expression plus wild-type DARC-Fc (negative control). (B) Induced yeast with wild-type DARC-Fc (positive control). (C) Induced yeast with an inactive DARC-Fc mutant (negative control).
(PDF)

Table S1 **Adhesion of PvDBP mutants to DARC.**
(PDF)

Table S2 **Immunization schedule.**
(PDF)

Table S3 **IC50 of DBPII glycosylation variants.**
(PDF)
