# Supplementary material for: Glycan Masking of Plasmodium vivax Duffy Binding Protein for Probing Protein Binding Function and Vaccine Development
Source: PLoS Pathog. 2013 Jun 13;9(6):e1003420. doi: 10.1371/journal.ppat.1003420 (PMC3681752; doi:10.1371/journal.ppat.1003420)
Supplement: Figure S1 — Sequence of PvDBPII wild type and glycoengineered variants. N-glycan sites are highlighted in yellow. The PvDBP sequence indicates the amino acid boundaries for the DNA and protein immunization constructs. The PvDBP construct boundaries for COS-7 assay are underlined and those for yeast display are shown in red. (PDF) [file ppat.1003420.s001.pdf]

Wild type

D H K K T I S S A I I N H A F L Q N T V M K N C N Y K R K R R E R D W D C N T
K K D V C I P D R R Y Q L C M K E L T N L V N N T D T N F H R D I T F R K L Y
L K R K L I Y D A A V E G D L L L K L N N Y R Y N K D F C K D I R W S L G D F
G D I I M G T D M E G I G Y S K V V E N N L R S I F G T D E K A Q Q R R K Q W
W N E S K A Q I W T A M M Y S V K K R L K G N F I W I C K L N V A V N I E P Q
I Y R W I R E W G R D Y V S E L P T E V Q K L K E K C D G K I N Y T D K K V C
K V P P C Q N A C K S Y D Q W I T R K K N Q W D V L S N K F I S V K N A E K V
Q T A G I V T P Y D I L K Q E L D E F N E V A F E N E I N K R D G A Y I E L C
V C S V E E A K K N T Q E V V T N V D N A A K S Q A T N S N P I S Q P V D S S
K A E K V P

STBP glycan

D H K K T I S S A I I N H A F L Q N T V M K N C N Y K R K R R E R D W D C N T
K K D V C I P D R R Y Q L C M K E L T N L V N N T D T N F H R D I T F R K L Y
L K R K L I Y D A A V E G D L L L K L N N Y R Y N K D F C K D I R W S L G D F
G D I I M G T D M E G I G Y S K V V E N N L R S I F G T D E K A Q Q R R K Q W
W N E S K A Q I W T A M M Y S V K K R L K G N F N W T C K L N V A V N I E P Q
I Y R W I R E W G R D Y V S E L P T E V Q K L K E K C D G K I N Y T D K K V C
K V P P C Q N A C K S Y D Q W I T R K K N Q W D V L S N K F I S V K N A E K V
Q T A G I V T P Y D I L K Q E L D E F N E V A F E N E I N K R D G A Y I E L C
V C S V E E A K K N T Q E V V T N V D N A A K S Q A T N S N P I S Q P V D S S
K A E K V P

P1

D H K K T I S S A I I N H A F L Q N T V M K N C N Y K R K R R E R D W D C N T
K K D V C I P D R R Y Q L C M K E L T N L V N N T D T N F H R N I T F R K L Y
L K R K L I Y D A A V E G D L L L K L N N Y R Y N K D F C K D I R W S L G D F
G D I I M G T D M E G I G Y S K V V E N N L R S I F G T D E K A Q Q R R K Q W
W N E S K A Q I W T A M M Y S V K K R L K G N F I W I C K L N V A V N I E P Q
I Y R W I R E W G R D Y V S E L P T E V Q K L K E K C D G K I N Y T D K K V C
K V P P C Q N A C K S Y D Q W I T R K K N Q W D V L S N K F I S V K N A S K V
Q T A G I V T P Y D I L K Q E L D E F N E T A F E N E I N K R D G A Y I E L C
V C S V E E A K K N T Q E V V T N V D N A A K S Q A T N S N P I S Q P V D S S
K A E K V P

Max

D H K K T I S S A I I N H A F L Q N T V M K N C N Y K R K R R E R D W D C N N
K S D V C I P D R R Y Q L C M K E L T N L V N N T D T N F H R N I T F R K L Y
L K R K L I Y D A A V E G D L L L K L N N Y R Y N K D F C K D I R W S L G D F
G D I I M G T D M E G I G Y S K V V E N N L R S I F G T D E N A S Q R R K Q W
W N E S K A Q I W T A M M Y S V K K R L K G N F I W I C K L N V A V N I E P Q
I Y R W I R E W G R D Y V S E L P T E V Q K L N E S C D G K I N Y T D K K V C
K V P P C Q N A C K S Y D Q W I T R K K N Q W D V L S N K F I S V K N A S K V
N T T G I V T P Y D I L K Q E L D E F N E T A F E N E I N K S D G A Y I E L C
V C S V E E A K K N T Q E V V T N V D N A A K S Q A T N S N P I S Q P V D S S
K A E K V P
