# Supplementary material for: Glycan Masking of Plasmodium vivax Duffy Binding Protein for Probing Protein Binding Function and Vaccine Development
Source: PLoS Pathog. 2013 Jun 13;9(6):e1003420. doi: 10.1371/journal.ppat.1003420 (PMC3681752; doi:10.1371/journal.ppat.1003420)
Supplement: Figure S2 — Alignment of PvDBP sequences. A multiple alignment was generated by Clustal 2.1 from 129 PvDBP sequences [28], [29] and used to generate the entropy scores at each amino acid position in the line-up. *, fully conserved residue; : strong conservation; . weak conservation. (PDF) [file ppat.1003420.s002.pdf]

```
gi|11120233|gb|AAG30847.1|         DHKKTISSAIINHAFLQNTVMKNCNYKRKRRERDWDCNTKKDVCIPDRRY
gi|12407747|gb|AAG53634.1|         DHKKTISSAIINHAFLQNTVMKNCNYKRKRRERDWDCNTKKDVCIPDRRY
gi|18766659|gb|AAL79050.1|AF46     ----------------------------------------------DRRY
gi|18766819|gb|AAL79130.1|AF46     ----------------------------------------------DRRY
gi|63146462|gb|AAY34059.1|         ----------------------------------------------DRRY
gi|63146616|gb|AAY34136.1|         ----------------------------------------------DRRY
gi|11138983|gb|AAG31571.1|         DHKKTISSAIINHAFLQNTVMKNCNYKRKRRERDWDCNTKKDVCIPDRRY
gi|73697858|gb|AAZ81532.1|         DHKKTISSAIINHAFLQNTVMKNCNYKRKRRERDWDCNTKKDVCIPDRRY
gi|73697846|gb|AAZ81526.1|         DHKKTISSAIINHAFLQNTVMKNCNYKRKRRERDWDCNTKKDVCIPDRRY
gi|12004297|gb|AAG43989.1|         -------------------------------ERDWDCNTKKDVCIPDRRY
gi|12004299|gb|AAG43990.1|         -------------------------------ERDWDCNTKKDVCIPDRRY
gi|73697866|gb|AAZ81536.1|         DHKKTISSAIINHAFLQNTVMKNCNYKRKRRERDWDCNTKKDVCIPDRRY
gi|73697848|gb|AAZ81527.1|         DHKKTISSAIINHAFLQNTVMKNCNYKRKRRERDWDCNTKKDVCIPDRRY
gi|73697856|gb|AAZ81531.1|         DHKKTISSAIINHAFLQNTVMKNCNYKRKRRERDWDCNTKKDVCIPDRRY
gi|73697862|gb|AAZ81534.1|         DHKKTISSAIINHAFLQNTVMKNCNYKRKRRERDWDCNTKKDVCIPDRRY
gi|160276|gb|AAA63423.1|           DHKKTISSAIINHAFLQNTVMKNCNYKRKRRERDWDCNTKKDVCIPDRRY
gi|73697844|gb|AAZ81525.1|         DHKKTISSAIINHAFLQNTVMKNCNYKRKRRERDWDCNTKKDVCIPDRRY
gi|339961368|pdb|3RRC|A            ---------------MGNTVMKNCNYKRKRRERDWDCNTKKDVCIPDRRY
gi|339961369|pdb|3RRC|B            ---------------MGNTVMKNCNYKRKRRERDWDCNTKKDVCIPDRRY
gi|255914751|dbj|BAH96577.1|       -------------------------------ERDWDCNTKKDVCIPDRRY
gi|224926624|gb|ACN69874.1|        ----TISSAIINHAFLQNTVMKNCNYKRKRRERDWDCNTKKDVCIPDRRY
gi|224926626|gb|ACN69875.1|        ----TISSAIINHAFLQNTVMKNCNYKRKRRERDWDCNTKKDVCIPDRRY
gi|224926628|gb|ACN69876.1|        ----TISSAIINHAFLQNTVMKNCNYKRKRRERDWDCNTKKDVCIPDRRY
gi|224926630|gb|ACN69877.1|        ----TISSAIINHAFLQNTVMKNCNYKRKRRERDWDCNTKKDVCIPDRRY
gi|224926632|gb|ACN69878.1|        ----TISSAIINHAFLQNTVMKNCNYKRKRRERDWDCNTKKDVCIPDRRY
gi|224926634|gb|ACN69879.1|        ----TISSAIINHAFLQNTVMKNCNYKRKRRERDWDCNTKKDVCIPDRRY
gi|224926636|gb|ACN69880.1|        ----TISSAIINHAFLQNTVMKNCNYKRKRRERDWDCNTKKDVCIPDRRY
gi|224926638|gb|ACN69881.1|        ----TISSAIINHAFLQNTVMKNCNYKRKRRERDWDCNTKKDVCIPDRRY
gi|224926640|gb|ACN69882.1|        ----TISSAIINHAFLQNTVMKNCNYKRKRRERDWDCNTKKDVCIPDRRY
gi|224926642|gb|ACN69883.1|        ----TISSAIINHAFLQNTVMKNCNYKRKRRERDWDCNTKKDGCIPERRY
gi|224926644|gb|ACN69884.1|        ----TISSAIINHAFLQNTVMKNCNYKRKRRERDWDCNTKKDVCIPDRRY
gi|224926646|gb|ACN69885.1|        ----TISSAIINHAFLQNTVMKNCNYKRKRRERDWDCNTKKDVCIPDRRY
gi|224926648|gb|ACN69886.1|        ----TISSAIINHAFLQNTVMKNCNYKRKRRERDWDCNTKKDVCIPDRRY
gi|224926650|gb|ACN69887.1|        ----TISSAIINHAFLQNTVMKNCNYKRKRRERDWDCNTKKDVCIPDRRY
gi|224926652|gb|ACN69888.1|        ----TISSAIINHAFLQNTVMKNCNYKRKRRERDWDCNTKKDVCIPDRRY
gi|224926654|gb|ACN69889.1|        ----TISSAIINHAFLQNTVMKNCNYKRKRRERDWDCNTKKDVCIPDRRY
gi|224926656|gb|ACN69890.1|        ----TISSAIINHAFLQNTVMKNCNYKRKRRERDWDCNTKKDVCIPDRRY
gi|224926658|gb|ACN69891.1|        ----TISSAIINHAFLQNTVMKNCNYKRKRRERDWDCNTKKDVCIPDRRY
gi|224926660|gb|ACN69892.1|        ----TISSAIINHAFLQNTVMKNCNYKRKRRERDWDCNTKKDVCIPDRRY
gi|224926662|gb|ACN69893.1|        ----TISSAIINHAFLQNTVMKNCNYKRKRRERDWDCNTKKDVCIPDRRY
gi|224926664|gb|ACN69894.1|        ----TISSAIINHAFLQNTVMKNCNYKRKRRERDWDCNTKKDVCIPDRRY
gi|224926666|gb|ACN69895.1|        ----TISSAIINHAFLQNTVMKNCNYKRKRRERDWDCNTKKDVCIPDRRY
gi|224926668|gb|ACN69896.1|        ----TISSAIINHAFLQNTVMKNCNYKRKRRERDWDCNTKKDVCIPDRRY
gi|224926670|gb|ACN69897.1|        ----TISSAIINHAFLQNTVMKNCNYKRKRRERDWDCNTKKDVCIPDRRY
gi|224926672|gb|ACN69898.1|        ----TISSAIINHAFLQNTVMKNCNYKRKRRERDWDCNTKKDVCIPDRRY
gi|224926674|gb|ACN69899.1|        ----TISSAIINHAFLQNTVMKNCNYKRKRRERDWDCNTKKDVCIPDRRY
gi|224926676|gb|ACN69900.1|        ----TISSAIINHAFLQNTVMKNCNYKRKRRERDWDCNTKKDVCIPDRRY
gi|224926678|gb|ACN69901.1|        ----TISSAIINHAFLQNTVMKNCNYKRKRRERDWDCNTKKDVCIPDRRY
gi|224926680|gb|ACN69902.1|        ----TISSAIINHAFLQNTVMKNCNYKRKRRERDWDCNTKKDVCIPDRRY
gi|224926682|gb|ACN69903.1|        ----TISSAIINHAFLQNTVMKNCNYKRKRRERDWDCNTKKDVCIPDRRY
gi|224926684|gb|ACN69904.1|        ----TISSAIINHAFLQNTVMKNCNYKRKRRERDWDCNTKKDVCIPDRRY
gi|224926686|gb|ACN69905.1|        ----TISSAIINHAFLQNTVMKNCNYKRKRRERDWDCNTKKDVCIPDRRY
gi|224926688|gb|ACN69906.1|        ----TISSAIINHAFLQNTVMKNCNYKRKRRERDWDCNTKKDVCIPDRRY
gi|224926690|gb|ACN69907.1|        ----TISSAIINHAFLQNTVMKNCNYKRKRRERDWDCNTKKDVCIPDRRY
gi|224926692|gb|ACN69908.1|        ----TISSAIINHAFLQNTVMKNCNYKRKRRERDWDCNTKKDVCIPDRRY
gi|224926694|gb|ACN69909.1|        ----TISSAIINHAFLQNTVMKNCNYKRKRRERDWDCNTKKDVCIPDRRY
gi|224926696|gb|ACN69910.1|        ----TISSAIINHAFLQNTVMKNCNYKRKRRERDWDCNTKKDVCIPDRRY
gi|224926698|gb|ACN69911.1|        ----TISSAIINHAFLQNTVMKNCNYKRKRRERDWDCNTKKDVCIPDRRY
gi|224926700|gb|ACN69912.1|        ----TISSAIINHAFLQNTVMKNCNYKRKRRERDWDCNTKKDVCIPDRRY
```

```
gi|224926702|gb|ACN69913.1|          ----TISSAIINHAFLQNTVMKNCNYKRKRRERDWDCNTKKDVCIPDRRY
gi|224926704|gb|ACN69914.1|          ----TISSAIINHAFLQNTVMKNCNYKRKRRERDWDCNTKKDVCIPDRRY
gi|224926706|gb|ACN69915.1|          ----TISSAIINHAFLQNTVMKNCNYKRKRRERDWDCNTKKDVCIPDRRY
gi|224926708|gb|ACN69916.1|          ----TISSAIINHALLQNTVMKNCNYKRKRRERDWGCNTKKDVCIPDRRY
gi|224926710|gb|ACN69917.1|          ----TISSAIINHAFLQNTVMKNCNYKRKRRERDWDCNTKKDVCIPDRRY
gi|224926714|gb|ACN69918.1|          ----TISSAIINHAFLQNTVMKNCNYKRKRRERDWDCNTKKDVCIPDRRY
gi|224926716|gb|ACN69919.1|          ----TISSAIINHAFLQNTVMKNCNYKRKRRERDWDCNTKKDVCIPDRRY
gi|224926718|gb|ACN69920.1|          ----TISSAIINHAFLQNTVMKNCNYKRKRRERDWDCNTKKDVCIPDRRY
gi|224926720|gb|ACN69921.1|          ----TISSAIINHAFLQNTVMKNCNYKRKRRERDWDCNTKKDVCIPDRRY
gi|224926722|gb|ACN69922.1|          ----TISSAIINHAFLQNTVMKNCNYKRKRRERDWDCNTKKDVCIPDRRY
gi|224926724|gb|ACN69923.1|          ----TISSAIINHAFLQNTVMKNCNYKRKRRERDWDCNTKKDVCIPDRRY
gi|224926726|gb|ACN69924.1|          ----TISSAIINHAFLQNTVMKNCNYKRKRRERDWDCNTKKDVCIPDRRY
gi|224926728|gb|ACN69925.1|          ----TISSAIINHAFLQNTVMKNCNYKRKRRERDWDCNTKKDVCIPDRRY
gi|224926730|gb|ACN69926.1|          ----TISSAIINHAFLQNTVMKNCNYKRKRRERDWDCNTKKDVCIPDRRY
gi|224926732|gb|ACN69927.1|          ----TISSAIINHAFLQNTVMKNCNYKRKRRERDWDCNTKKDVCIPDRRY
gi|224926734|gb|ACN69928.1|          ----TISSAIINHAFLQNTVMKNCNYKRKRRERDWDCNTKKDVCIPDRRY
gi|224926736|gb|ACN69929.1|          ----TISSAIINHAFLQNTVMKNCNYKRKRRERDWDCNTKKDVCIPDRRY
gi|224926738|gb|ACN69930.1|          ----TISSAIINHAFLQNTVMKNCNYKRKRRERDWDCNTKKDVCIPDRRY
gi|224926740|gb|ACN69931.1|          ----TISSAIINHAFLQNTVMKNCNYKRKRRERDWDCNTKKDVCIPDRRY
gi|224926742|gb|ACN69932.1|          ----TISSAIINHAFLQNTVMKNCNYKRKRRERDWDCNTKKDVCIPDRRY
gi|224926744|gb|ACN69933.1|          ----TISSAIINHAFLQNTVMKNCNYKRKRRERDWDCNTKKDVCIPDRRY
gi|224926746|gb|ACN69934.1|          ----TISSAIINHAFLQNTVMKNCNYKRKRRERDWDCNTKKDVCIPDRRY
gi|224926748|gb|ACN69935.1|          ----TISSAIINHAFLQNTVMKNCNYKRKRRERDWDCNTKKDVCIPDRRY
gi|224926750|gb|ACN69936.1|          ----TISSAIINHAFLQNTVMKNCNYKRKRRERDWDCNTKKDVCIPDRRY
gi|224926752|gb|ACN69937.1|          ----TISSAIINHAFLQNTVMKNCNYKRKRRERDWDCNTKKDVCIPDRRY
gi|224926754|gb|ACN69938.1|          ----TISSAIINHAFLQNTVMKNCNYKRKRRERDWDCNTKKDVCIPDRRY
gi|224926756|gb|ACN69939.1|          ----TISSAIINHAFLQNTVMKNCNYKRKRRERDWDCNTKKDVCIPDRRY
gi|224926758|gb|ACN69940.1|          ----TISSAIINHAFLQNTVMKNCNYKRKRRERDWDCNTKKDVCIPDRRY
gi|224926760|gb|ACN69941.1|          ----TISSAIINHAFLQNTVMKNCNYKRKRRERDWDCNTKKDVCIPDRRY
gi|224926762|gb|ACN69942.1|          ----TISSAIINHAFLQNTVMKNCNYKRKRRERDWDCNTKKDVCIPDRRY
gi|224926764|gb|ACN69943.1|          ----TISSAIINHAFLQNTVMKNCNYKRKRRERDWDCNTKKDVCIPDRRY
gi|224926766|gb|ACN69944.1|          ----TISSAIINHAFLQNTVMKNCNYKRKRRERDWDCNTKKDVCIPDRRY
gi|224926768|gb|ACN69945.1|          ----TISSAIINHAFLQNTVMKNCNYKRKRRERDWDCNTKKDVCIPDRRY
gi|224926770|gb|ACN69946.1|          ----TISSAIINHAFLQNTVMKNCNYKRKRRERDWDCNTKKDVCIPDRRY
gi|224926772|gb|ACN69947.1|          ----TISSAIINHAFLQNTVMKNCNYKRKRRERDWDCNTKKDVCIPDRRY
gi|224926774|gb|ACN69948.1|          ----TISSAIINHAFLQNTVMKNCNYKRKRRERDWDCNTKKDVCIPDRRY
gi|224926776|gb|ACN69949.1|          ----TISSAIINHAFLQNTVMKNCNYKRKRRERDWDCNTKKDVCIPDRRY
gi|224926778|gb|ACN69950.1|          ----TISSAIINHAFLQNTVMKNCNYKRKRRERDWDCNTKKDVCIPDRRY
gi|224926780|gb|ACN69951.1|          ----TISSAIINHAFLQNTVMKNCNYKRKRRERDWDCNTKKDVCIPDRRY
gi|224926782|gb|ACN69952.1|          ----TISSAIINHAFLQNTVMKNCNYKRKRRERDWDCNTKKDVCIPDRRY
gi|224926784|gb|ACN69953.1|          ----TISSAIINHAFLQNTVMKNCNYKRKRRERDWDCNTKKDVCIPDRRY
gi|224926786|gb|ACN69954.1|          ----TISSAIINHAFLQNTVMKNCNYKRKRRERDWDCNTKKDVCIPDRRY
gi|224926788|gb|ACN69955.1|          ----TISSAIINHAFLQNTVMKNCNYKRKRRERDWDCNTKKDVCIPDRRY
gi|224926790|gb|ACN69956.1|          ----TISSAIINHAFLQNTVMKNCNYKRKRRERDWDCNTKKDVCIPDRRY
gi|224926792|gb|ACN69957.1|          ----TISSAIINHAFLQNTVMKNCNYKRKRRERDWDCNTKKDVCIPDRRY
gi|224926794|gb|ACN69958.1|          ----TISSAIINHAFLQNTVMKNCNYKRKRRERDWDCNTKKDVCIPDRRY
gi|224926796|gb|ACN69959.1|          ----TISSAIINHAFLQNTVMKNCNYKRKRRERDWDCNTKKDVCIPDRRY
gi|224926798|gb|ACN69960.1|          ----TISSAIINHAFLQNTVMKNCNYKRKRRERDWDCNTKKDVCIPDRRY
gi|224926800|gb|ACN69961.1|          ----TISSAIINHAFLQNTVMKNCNYKRKRRERDWDCNTKKDVCIPDRRY
gi|224926802|gb|ACN69962.1|          ----TISSAIINHAFLQNTVMKNCNYKRKRRERDWDCNTKKDVCIPDRRY
gi|224926804|gb|ACN69963.1|          ----TISSAIINHAFLQNTVMKNCNYKRKRRERDWDCNTKKDVCIPDRRY
gi|224926806|gb|ACN69964.1|          ----TISSAIINHAFLQNTVMKNCNYKRKRRERDWDCNTKKDVCIPDRRY
gi|224926808|gb|ACN69965.1|          ----TISSAIINHAFLQNTVMKNCNYKRKRRERDWDCNTKKDVCIPDRRY
gi|224926810|gb|ACN69966.1|          ----TISSAIINHAFLQNTVMKNCNYKRKRRERDWDCNTKKDVCIPDRRY
gi|224926812|gb|ACN69967.1|          ----TISSAIINHAFLQNTVMKNCNYKRKRRERDWDCNTKKDVCIPDRRY
gi|224926814|gb|ACN69968.1|          ----TISSAIINHAFLQNTVMKNCNYKRKRRERDWDCNTKKDVCIPDRRY
gi|224926816|gb|ACN69969.1|          ----TISSAIINHAFLQNTVMKNCNYKRKRRERDWDCNTKKDVCIPDRRY
gi|224926818|gb|ACN69970.1|          ----TISSAIINHAFLQNTVMKNCNYKRKRRERDWDCNTKKDVCIPDRRY
gi|224926820|gb|ACN69971.1|          ----TISSAIINHAFLQNTVMKNCNYKRKRRERDWDCNTKKDVCIPDRRY
```

```
gi|213868281|gb|ACJ54187.1|         DHKKTISSAIINHAFLQNTVMKNCNYKRKRRERDWDCNTKKDVCIPDRRY
gi|213868283|gb|ACJ54188.1|         DHKKTISSAIINHAFLQNTVMKNCNYKRKRRERDWDCNTKKDVCIPDRRY
gi|213868285|gb|ACJ54189.1|         DHKKTISSAIINHAFLQNTVMKNCNYKRKRRERDWDCNTKKDVCIPDRRY
gi|213868287|gb|ACJ54190.1|         DHKKTISSAIINHAFLQNTVMKNCNYKRKRRERDWDCNTKKDVCIPDRRY
gi|213868289|gb|ACJ54191.1|         DHKKTISSAIINHAFLQNTVMKNCNYKRKRRERDWDCNTKKDVCIPDRRY
gi|213868291|gb|ACJ54192.1|         DHKKTISSAIINHAFLQNTVMKNCNYKRKRRERDWDCNTKKDVCIPDRRY
gi|213868293|gb|ACJ54193.1|         DHKKTISSAIINHAFLQNTVMKNCNYKRKRRERDWDCNTKKDVCIPDRRY
gi|213868295|gb|ACJ54194.1|         DHKKTISSAIINHAFLQNTVMKNCNYKRKRRERDWDCNTKKDVCIPDRRY
gi|213868297|gb|ACJ54195.1|         DHKKTISSAIINHAFLQNTVMKNCNYKRKRRERDWDCNTKKDVCIPDRRY
gi|213868299|gb|ACJ54196.1|         DHKKTISSAIINHAFLQNTVMKNCNYKRKRRERDWDCNTKKDVCIPDRRY
gi|213868301|gb|ACJ54197.1|         DHKKTISSAIINHAFLQNTVMKNCNYKRKRRERDWDCNTKKDVCIPDRRY
                                                                                :***

gi|11120233|gb|AAG30847.1|          QLCMKELTNLVNNTDTNFHSDITFRKLYLKRKLIYDAAVEGDLLLKLNNY
gi|12407747|gb|AAG53634.1|          QLCMKELTNLVNNTDTNFHSDITFRKLYLKRKLIYDAAVEGDLLLKLNNY
gi|18766659|gb|AAL79050.1|AF46      QLCMKELTNLVNNTDTNFHRDITFRKLYLKRKLIYDAAVEGDLLLKLNNY
gi|18766819|gb|AAL79130.1|AF46      QLCMKELTNLVNNTDTNFHSDITFRKLYLKRKLIYDAAVEGDLLLKLNNY
gi|63146462|gb|AAY34059.1|          QLCMKELTNLVNNTDTNFHSDITFRKLYLKRKLIYDAAVEGDLLLKLNNY
gi|63146616|gb|AAY34136.1|          QLCMKELTNLVNNTDTNFHRDITFRKLYLKRKLIYDAAVEGDLLLKLNNY
gi|11138983|gb|AAG31571.1|          QLCMKELTNLVNNTDTNFHSDITFRKLYLKRKLIYDAAVEGDLLLKLNNY
gi|73697858|gb|AAZ81532.1|          QLCMKELTNLVNNTDTNFHSDITFRKLYLKRKLIYDAAVEGDLLLKLNNY
gi|73697846|gb|AAZ81526.1|          QLCMKELTNLVNNTDTNFHRDITFRKLYLKRKLIYDAAVEGDLLLKLNNY
gi|12004297|gb|AAG43989.1|          QLCMKELTNLVNNTDTNFHRDITFRKLYLKRKLIYDAAVEGDLLLKLNNY
gi|12004299|gb|AAG43990.1|          QLCMKELTNLVNNTDTNFHRDITFRKLYLKRKLIYDAAVEGDLLLKLNNY
gi|73697866|gb|AAZ81536.1|          QLCMKELTNLVNNTDTNFHRDITFRKLYLKRKLIYDAAVEGDLLLKLNNY
gi|73697848|gb|AAZ81527.1|          QLCMKELTNLVNNTDTNFHRDITFRKLYLKRKLIYDAAVEGDLLLKLNNY
gi|73697856|gb|AAZ81531.1|          QLCMKELTNLVNNTDTNFHSDITFRKLYLKRKLIYDAAVEGDLLLKLNNY
gi|73697862|gb|AAZ81534.1|          QLCMKELTNLVNNTDTNLHSDITFRKLYLKRKLIYDAAVEGDLLLKLNNY
gi|160276|gb|AAA63423.1|            QLCMKELTNLVNNTDTNFHRDITFRKLYLKRKLIYDAAVEGDLLLKLNNY
gi|73697844|gb|AAZ81525.1|          QLCMKELTNLVNNTDTNFHRDITFRKLYLKRKLIYDAAVEGDLLLKLNNY
gi|339961368|pdb|3RRC|A             QLCMKELTNLVNNTDTNFHRDITFRKLYLKRKLIYDAAVEGDLLLKLNNY
gi|339961369|pdb|3RRC|B             QLCMKELTNLVNNTDTNFHRDITFRKLYLKRKLIYDAAVEGDLLLKLNNY
gi|255914751|dbj|BAH96577.1|        QLCMKELTNLVNNTDTNFHRDITFRKLYLKRKLIYDAAVEGDLLLKLNNY
gi|224926624|gb|ACN69874.1|         QLCMKELTNLVNNTDTNFHRDITFRKLYLKRKLIYDAAVEGDLLLKLNNY
gi|224926626|gb|ACN69875.1|         QLCMKELTNLVNNTDTNFHRDITFRKLYLKRKLIYDAAVEGDLLLKLNNY
gi|224926628|gb|ACN69876.1|         QLCMKELTNLVNNTDTNFHRDITFRKLYLKRKLIYDAAVEGDLLLKLNNY
gi|224926630|gb|ACN69877.1|         QLCMKELTNLVNNTDTNFHSDITFRKLYLKRKLIYDAAVEGDLLLKLNNY
gi|224926632|gb|ACN69878.1|         QLCMKELTNLVNNTDTNFHRDITFRKLYLKRKLIYDAAVEGDLLLKLNNY
gi|224926634|gb|ACN69879.1|         QLCMKELTNLVNNTDTNFHRDITFRKLYLKRKLIYDAAVEGDLLLKLNNY
gi|224926636|gb|ACN69880.1|         QLCMKELTNLVNNTDTNFHRDITFRKLYLKRKLIYDAAVEGDLLLKLNNY
gi|224926638|gb|ACN69881.1|         QLCMKELTNLVNNTDTNFHRDITFRKLYLKRKLIYDAAVEGDLLLKLNNY
gi|224926640|gb|ACN69882.1|         QLCMKELTNLVNNTDTNFHRDITFRKLYLKRKLIYDAAVEGDLLLKLNNY
gi|224926642|gb|ACN69883.1|         QLCMKELTNLVNNTDTNFHRDITFRKLYLKRKLIYDAAVEGDLLLKLNNY
gi|224926644|gb|ACN69884.1|         QLCMKELTNLVNNTDTNFHRDITFRKLYLKRKLIYDAAVEGDLLLKLNNY
gi|224926646|gb|ACN69885.1|         QLCMKELTNLVNNTDTNFHRDITFRKLYLKRKLIYDAAVEGDLLLKLNNY
gi|224926648|gb|ACN69886.1|         QLCMKELTNLVNNTDTNFHRDITFRKLYLKRKLIYDAAVEGDLLLKLNNY
gi|224926650|gb|ACN69887.1|         QLCMKELTNLVNNTDTNFHRDITFRKLYLKRKLIYDAAVEGDLLLKLNNY
gi|224926652|gb|ACN69888.1|         QLCMKELTNLVNNTDTNFHRDITFRKLYLKRKLIYDAAVEGDLLLKLNNY
gi|224926654|gb|ACN69889.1|         QLCMKELTNLVNNTDTNFHRDITFRKLYLKRKLIYDAAVEGDLLLKLNNY
gi|224926656|gb|ACN69890.1|         QLCMKELTNLVNNTDPNFHRDITFRKLYLKRKLIYDAAVEGDLLLKLNNY
gi|224926658|gb|ACN69891.1|         QLCMKELTNLVNNTDTNFHRDITFRKLYLKRKLIYDAAVEGDLLLKLNNY
gi|224926660|gb|ACN69892.1|         QLCMKELTNLVNNTDTNFHRDITFRKLYLKRKLIYDAAVEGDLLLKLNNY
gi|224926662|gb|ACN69893.1|         QLCMKELTNLVNNTDTNFHRDITFRKLYLKRKLIYDAAVEGDLLLKLNNY
gi|224926664|gb|ACN69894.1|         QLCMKELTNLVNNTDTNLHSDITFRKLYLKRKLIYDAAVEGDLLLKLNNY
gi|224926666|gb|ACN69895.1|         QLCMKELTNLVNNTDTNFHRDITFRKLYLKRKLIYDAAVEGDLLLKLNNY
gi|224926668|gb|ACN69896.1|         QLCMKELTNLVNNTDTNFHRDITFRKLYLKRKLIYDAAVEGDLLLKLNNY
gi|224926670|gb|ACN69897.1|         QLCMKELTNLVNNTDTNFHRDITFRKLYLKRKLIYDAAVEGDLLLKLNNY
gi|224926672|gb|ACN69898.1|         QLCMKELTNLVNNTDTNFHRDITFRKLYLKRKLIYDAAVEGDLLLKLNNY
gi|224926674|gb|ACN69899.1|         QLCMKELTNLVNNTDTNFHRDITFRKLYLKRKLIYDAAVEGDLLLKLNNY
```

```
gi|224926676|gb|ACN69900.1|         QLCMKELTNLVNNTDTNFHRDITFRKLYLKRKLIYDAAVEGDLLLKLNNY
gi|224926678|gb|ACN69901.1|         QLCMKELTNLVNNTDTNFHRDITFRKLYLKRKLIYDAAVEGDLLLKLNNY
gi|224926680|gb|ACN69902.1|         QLCMKELTNLVNNTDTNLHSDITFRKLYLKRKLIYDAAVEGDLLLKLNNY
gi|224926682|gb|ACN69903.1|         QLCMKELTNLVNNTDTNFHRDITFRKLYLKRKLIYDAAVEGDLLLKLNNY
gi|224926684|gb|ACN69904.1|         QLCMKELTNLVNNTDTNFHRDITFRKLYLKRKLIYDAAVEGDLLLKLNNY
gi|224926686|gb|ACN69905.1|         QLCMKELTNLVNNTDTNFHRDITFRKLYLKRKLIYDAAVEGDLLLKLNNY
gi|224926688|gb|ACN69906.1|         QLCMKELTNLVNNTDTNFHSDITFRKLYLKRKLIYDAAVEGDLLLKLNNY
gi|224926690|gb|ACN69907.1|         QLCMKELTNLVNNTDTNFHRDITFRKLYLKRKLIYDAAVEGDLLLKLNNY
gi|224926692|gb|ACN69908.1|         QLCMKELTNLVNNTDTNFHRDITFRKLYLKRKLIYDAAVEGDLLLKLNNY
gi|224926694|gb|ACN69909.1|         QLCMKELTNLVNNTDTNFHRDITFRKLYLKRKLIYDAAVEGDLLLKLNNY
gi|224926696|gb|ACN69910.1|         QLCMKELTNLVNNTDTNFHRDITFRKLYLKRKLIYDAAVEGDLLLKLNNY
gi|224926698|gb|ACN69911.1|         QLCMKELTNLVNNTDTNFHRDITFRKLYLKRKLIYDAAVEGDLLLKLNNY
gi|224926700|gb|ACN69912.1|         QLCMKELTNLVNNTDTNFHRDITFRKLYLKRKLIYDAAVEGDLLLKLNNY
gi|224926702|gb|ACN69913.1|         QLCMKELTNLVNNTDTNFHRDITFRKLYLKRKLIYDAAVEGDLLLKLNNY
gi|224926704|gb|ACN69914.1|         QLCMKELTNLVNNTDTNFHRDITFRKLYLKRKLIYDAAVEGDLLLKLNNY
gi|224926706|gb|ACN69915.1|         QLCMKELTNLVNNTDTNFHRDITFRKLYLKRKLIYDAAIKGDLLLKLNNY
gi|224926708|gb|ACN69916.1|         QLCMKELTNLVNNTDTNFHRDITFRKLYLKRKLIYDAAVEGDLLLKLNNY
gi|224926710|gb|ACN69917.1|         QLCMKELTNLVNNTDTNFHRDITFRKLYLKRKLIYDAAVEGDLLLKLNNY
gi|224926714|gb|ACN69918.1|         QLCMKELTNLVNNTDTNFHRDITFRKLYLKRKLIYDAAVEGDLLLKLNNY
gi|224926716|gb|ACN69919.1|         QLCMKELTNLVNNTDTNFHRDITFRKLYLKRKLIYDAAVEGDLLLKLNNY
gi|224926718|gb|ACN69920.1|         QLCMKELTNLVNNTDTNFHRDITFRKLYLKRKLIYDAAVEGDLLLKLNNY
gi|224926720|gb|ACN69921.1|         QLCMKELTNLVNNTDTNFHRDITFRKLYLKRKLIYDAAVEGDLLLKLNNY
gi|224926722|gb|ACN69922.1|         QLCMKELTNLVNNTDTNFHRDITFRKLYLKRKLIYDAAVEGDLLLKLNNY
gi|224926724|gb|ACN69923.1|         QLCMKELTNLVNNTDTNFHRDITFRKLYLKRKLIYDAAVEGDLLLKLNNY
gi|224926726|gb|ACN69924.1|         QLCMKELTNLVNNTDTNFHRDITFRKLYLKRKLIYDAAVEGDLLLKLNNY
gi|224926728|gb|ACN69925.1|         QLCMKELTNLVNNTDTNFHRDITFRKLYLKRKLIYDAAVEGDLLLKLNNY
gi|224926730|gb|ACN69926.1|         QLCMKELTNLVNNTDTNFHRDITFRKLYLKRKLIYDAAVEGDLLLKLNNY
gi|224926732|gb|ACN69927.1|         QLCMKELTNLVNNTDTNFHRDITFRKLYLKRKLIYDAAVEGDLLLKLNNY
gi|224926734|gb|ACN69928.1|         QLCMKELTNLVNNTDTNFHRDITFRKLYLKRKLIYDAAVEGDLLLKLNNY
gi|224926736|gb|ACN69929.1|         QLCMKELTNLVNNTDTNFHRDITFRKLYLKRKLIYDAAVEGDLLLKLNNY
gi|224926738|gb|ACN69930.1|         QLCMKELTNLVNNTDTNFHRDITFRKLYLKRKLIYDAAVEGDLLLKLNNY
gi|224926740|gb|ACN69931.1|         QLCMKELTNLVNNTDTNFHRDITFRKLYLKRKLIYDAAVEGDLLLKLNNY
gi|224926742|gb|ACN69932.1|         QLCMKELTNLVNNTDTNFHRDITFRKLYLKRKLIYDAAVEGDLLLKLNNY
gi|224926744|gb|ACN69933.1|         QLCMKELTNLVNNTDTNFHRDITFRKLYLKRKLIYDAAVEGDLLLKLNNY
gi|224926746|gb|ACN69934.1|         QLCMKELTNLVNNTDTNFHRDITFRKLYLKRKLIYDAAVEGDLLLKLNNY
gi|224926748|gb|ACN69935.1|         QLCMKELTNLVNNTDTNFHRDITFRKLYLKRKLIYDAAVEGDLLLKLNNY
gi|224926750|gb|ACN69936.1|         QLCMKELTNLVNNTDTNFHRDITFRKLYLKRKLIYDAAVEGDLLLKLNNY
gi|224926752|gb|ACN69937.1|         QLCMKELTNLVNNTDTNFHRDITFRKLYLKRKLIYDAAVEGDLLLKLNNY
gi|224926754|gb|ACN69938.1|         QLCMKELTNLVNNTDTNFHRDITFRKLYLKRKLIYDAAVEGDLLFKLNNY
gi|224926756|gb|ACN69939.1|         QLCMKELTNLVNNTDTNFHRDITFRKLYLKRKLIYDAAVEGDLLLKLNNY
gi|224926758|gb|ACN69940.1|         QLCMKELTNLVNNTDTNFHRDITFRKLYLKRKLIYDAAVEGDLLLKLNNY
gi|224926760|gb|ACN69941.1|         QLCMKELTNLVNNTDTNFHSDITFRKLYLKRKLIYDAAVEGDLLFKLNNY
gi|224926762|gb|ACN69942.1|         QLCMKELTNLVNNTDTNFHSDITFRKLYLKRKLIYDAAVEGDLLFKLNNY
gi|224926764|gb|ACN69943.1|         QLCMKELTNLVNNTDTNFHRDITFRKLYLKRKLIYDAAVEGDLLLKLNNY
gi|224926766|gb|ACN69944.1|         QLCMKELTNLVNNTDTNFHRDITFRKLYLKRKLIYDAAVEGDLLFKLNNY
gi|224926768|gb|ACN69945.1|         QLCMKELTNLVNNTDTNFHRDITFRKLYLKRKLIYDAAVEGDLLLKLNNY
gi|224926770|gb|ACN69946.1|         QLCMKELTNLVNNTDTNFHRDITFRKLYLKRKLIYDAAVEGDLLLKLNNY
gi|224926772|gb|ACN69947.1|         QLCMKELTNLVNNTDTNFHRDITFRKLYLKRKLIYDAAVEGDLLFKLNNY
gi|224926774|gb|ACN69948.1|         QLCMKELTNLVNNTDTNFHRDITFRKLYLKRKLIYDAAVEGDLLLKLNNY
gi|224926776|gb|ACN69949.1|         QLCMKELTNLVNNTDTNFHSDITFRKLYLKRKLIYDAAVEGDLLLKLNNY
gi|224926778|gb|ACN69950.1|         QLCMKELTNLVNNTDTNFHSDITFRKLYLKRKLIYDAAVEGDLLLKLNNY
gi|224926780|gb|ACN69951.1|         QLCMKELTNLVNNTDTNFHRDITFRKLYLKRKLIYDAAVEGDLLLKLNNY
gi|224926782|gb|ACN69952.1|         QLCMKELTNLVNNTDTNFHRDITFRKLYLKRKLIYDAAVEGDLLLKLNNY
gi|224926784|gb|ACN69953.1|         QLCMKELTNLVNNTDTNFHRDITFRKLYLKRKLIYDAAVEGDLLLKLNNY
gi|224926786|gb|ACN69954.1|         QLCMKELTNLVNNTDTNFHRDITFRKLYLKRKLIYDAAVEGDLLLKLNNY
gi|224926788|gb|ACN69955.1|         QLCMKELTNLVNNTDTNFHRDITFRKLYLKRKLIYDAAVEGDLLLKLNNY
gi|224926790|gb|ACN69956.1|         QLCMKELTNLVNNTDTNFHRDITFRKLYLKRKLIYDAAVEGDLLLKLNNY
gi|224926792|gb|ACN69957.1|         QLCMKELTNLVNNTDTNFHRDITFRKLYLKRKLIYDAAVEGDLLLKLNNY
gi|224926794|gb|ACN69958.1|         QLCMKELTNLVNNTDTNFHRDITFRKLYLKRKLIYDAAVEGDLLLKLNNY
```

```
gi|224926796|gb|ACN69959.1|        QLCMKELTNLVNNTDTNFHRDITFRKLYLKRKLIYDAAVEGDLLLKLNNY
gi|224926798|gb|ACN69960.1|        QLCMKELTNLVNNTDTNFHRDITFRKLYLKRKLIYDAAVEGDLLLKLNNY
gi|224926800|gb|ACN69961.1|        QLCMKELTNLVNNTDTNFHRDITFRKLYLKRKLIYDAAVEGDLLLKLNNY
gi|224926802|gb|ACN69962.1|        QLCMKELTNLVNNTDTNFHRDITFRKLYLKRKLIYDAAVEGDLLLKLNNY
gi|224926804|gb|ACN69963.1|        QLCMKELTNLVNNTDTNFHSDITFRKLYLKRKLIYDAAVEGDLLLKLNNY
gi|224926806|gb|ACN69964.1|        QLCMKELTNLVNNTDTNFHRDITFRKLYLKRKLIYDAAVEGDLLLKLNNY
gi|224926808|gb|ACN69965.1|        QLCMKELTNLVNNTDTNLHSDITFRKLYLKRKLIYDAAVEGDLLLKLNNY
gi|224926810|gb|ACN69966.1|        QLCMKELTNLVNNTDTNFHRDITFRKLYLKRKLIYDAAVEGDLLLKLNNY
gi|224926812|gb|ACN69967.1|        QLCMKELTNLVNNTDTNFHRDITFRKLYLKRKLIYDAAVEGDLLLKLNNY
gi|224926814|gb|ACN69968.1|        QLCMKELTNLVNNTDTNFHRDITFRKLYLKRKLIYDAAVEGDLLLKLNNY
gi|224926816|gb|ACN69969.1|        QLCMKELTNLVNNTDTNFHRDITFRKLYLKRKLIYDAAVEGDLLLKLNNY
gi|224926818|gb|ACN69970.1|        QLCMKELTNLVNNTDTNFHRDITFRKLYLKRKLIYDAAVEGDLLLKLNNY
gi|224926820|gb|ACN69971.1|        QLCMKELTNLVNNTDTNFHRDITFRKLYLKRKLIYDAAVEGDLLLKLNNY
gi|213868281|gb|ACJ54187.1|        QLCMKELTNLVNNTDTNLHSDITFRKLYLKRKLIYDAAVEGDLLLKLNNY
gi|213868283|gb|ACJ54188.1|        QLCMKELTNLVNNTDTNFHSDITFRKLYLKRKLIYDAAVEGDLLLKLNNY
gi|213868285|gb|ACJ54189.1|        QLCMKELTNLVNNTDTNFHRDITFRKLYLKRKLIYDAAVEGDLLLKLNNY
gi|213868287|gb|ACJ54190.1|        QLCMKELTNLVNNTDTNFHRDITFRKLYLKRKLIYDAAVEGDLLLKLNNY
gi|213868289|gb|ACJ54191.1|        QLCMKELTNLVNNTDTNFHRDITFRKLYLKRKLIYDAAVEGDLLLKLNNY
gi|213868291|gb|ACJ54192.1|        QLCMKELTNLVNNTDTNFHRDITFRKLYLKRKLIYDAAVEGDLLLKLNNY
gi|213868293|gb|ACJ54193.1|        QLCMKELTNLVNNTDTNFHRDITFRKLYLKRKLIYDAAVEGDLLLKLNNY
gi|213868295|gb|ACJ54194.1|        QLCMKELTNLVNNTDTNFHRDITFRKLYLKRKLIYDAAVEGDLLLKLNNY
gi|213868297|gb|ACJ54195.1|        QLCMKELTNLVNNTDTNFHRDITFRKLYLKRKLIYDAAVEGDLLLKLNNY
gi|213868299|gb|ACJ54196.1|        QLCMKELTNLVNNTDTNFHRDITFRKLYLKRKLIYDAAVEGDLLLKLNNY
gi|213868301|gb|ACJ54197.1|        QLCMKELTNLVNNTDTNFHRDITFRKLYLKRKLIYDAAVEGDLLLKLNNY
                                   ***************.*:* ******************::****:*****

gi|11120233|gb|AAG30847.1|         RYNKDFCKDIRWSLGDFGDIIMGTDMEGIGYSKVVENNSRSIFGTGEKAQ
gi|12407747|gb|AAG53634.1|         RYNKDFCKDIRWSLGDFGDIIMGTDMEGIGYSKVVENNLRSIFGTGEKAQ
gi|18766659|gb|AAL79050.1|AF46     RYNKDFCKDIRWSLGDFGDIIMGTDMEGIGYSKVVENNLRSIFGTDEKAQ
gi|18766819|gb|AAL79130.1|AF46     RYNKDFCKDIRWSLGDFGDIIMGTDMEGIGYSKVVENNLRSIFGTGEKAQ
gi|63146462|gb|AAY34059.1|         RYNKDFCKDIRWSLGDFGDIIMGTDMEGIGYSKVVENNLRSIFGTGEKAQ
gi|63146616|gb|AAY34136.1|         RYNKDFCKDIRWSLGDFGDIIMGTDMEGIGYSKVVENNLRSIFGTDEKAQ
gi|11138983|gb|AAG31571.1|         RYNKDFCKDIRWSLGDFGDIIMGTDMEGIGYSKVVENNLRSIFGTGEKAQ
gi|73697858|gb|AAZ81532.1|         RYNKDFCKDIRWSLGDFGDIIMGTDMEGIGYSKVVENNLRSIFGTDEKAQ
gi|73697846|gb|AAZ81526.1|         RYNKDFCKDIRWSLGDFGDIIMGTDMEGIGYSKVVENNLRSIFGTGEKAQ
gi|12004297|gb|AAG43989.1|         RYNKDFCKDIRWSLGDFGDIIMGTDMEGIGYSEVVENNLRSIFGTGEKAQ
gi|12004299|gb|AAG43990.1|         RYNKDFCKDIRWSLGDFGDIIMGTDMEGIGYSKVVENNLRSIFGTDEKAQ
gi|73697866|gb|AAZ81536.1|         RYNKDFCKDIRWSLGDFGDIIMGTDMEGIGYSKVVENNLRSIFGTGEKAQ
gi|73697848|gb|AAZ81527.1|         RYNKDFCKDIRWSLGDFGDIIMGTDMEGIGYSKVVENNLRSIFGTDEKAQ
gi|73697856|gb|AAZ81531.1|         RYNKDFCKDIRWSLGDFGDIIMGTDMEGIGYSKVVENNLRSIFGTGKNAQ
gi|73697862|gb|AAZ81534.1|         RYNKDFCKDIRWSLGDFGDIIMGTDMEGIGYSKVVENNLRSIFGTGKNAQ
gi|160276|gb|AAA63423.1|           RYNKDFCKDIRWSLGDFGDIIMGTDMEGIGYSKVVENNLRSIFGTDEKAQ
gi|73697844|gb|AAZ81525.1|         RYNKDFCKDIRWSLGDFGDIIMGTDMEGIGYSKVVENNLRSIFGTDEKAQ
gi|339961368|pdb|3RRC|A            RYNKDFCKDIRWSLGDFGDIIMGTDMEGIGYSKVVENNLRSIFGTDEKAQ
gi|339961369|pdb|3RRC|B            RYNKDFCKDIRWSLGDFGDIIMGTDMEGIGYSKVVENNLRSIFGTDEKAQ
gi|255914751|dbj|BAH96577.1|       RYNKDFCKDIRWSLGDFGDIIMGTDMEGIGYSKVVENNLRSIFGTGEKAQ
gi|224926624|gb|ACN69874.1|        RYNKDFCKDIRWSLGDFGDIIMGTDMEGIGYSEVVENNLRSIFGTGEQAQ
gi|224926626|gb|ACN69875.1|        RYNKDFCKDIRWSLGDFGDIIMGTDMEGIGYSEVVENNLRSIFGTGEKAQ
gi|224926628|gb|ACN69876.1|        RYNKDFCKDIRWSLGDFGDIIMGTDMEGIGYSKVVENNLRSIFGTGEKAQ
gi|224926630|gb|ACN69877.1|        RYNKDFCKDIRWSLGDFGDIIMGTDMEGIGYSKVVENNLRSIFGTGKNAQ
gi|224926632|gb|ACN69878.1|        RYNKDFCKDIRWSLGDFGDIIMGTDMEGIGYSKVVENNLRSIFGTGEKAQ
gi|224926634|gb|ACN69879.1|        RYNKDFCKDIRWSLGDFGDIIMGTDMEGIGYSEVVENNLRSIFGTGKNAQ
gi|224926636|gb|ACN69880.1|        RYNKDFCKDIRWSLGDFGDIIMGTDMEGIGYSEVVENNLRSIFGTGEKAQ
gi|224926638|gb|ACN69881.1|        RYNKDFCKDIRWSLGDFGDIIMGTDMEGIGYSKVVENNLRSIFGTGEKAQ
gi|224926640|gb|ACN69882.1|        RYNKDFCKDIRWSLGDFGDIIMGTDMEGIGYSEVVENNLRSIFGTGKNAQ
gi|224926642|gb|ACN69883.1|        RYNKDFCKDIRWSLGDFGDIIMGTDMEGIGYSEVVENNLRSIFGTGEKAQ
gi|224926644|gb|ACN69884.1|        RYNKDFCKDIRWSLGDFGDIIMGTDMEGIGYSEVVENNLRSIFGTGEKAQ
gi|224926646|gb|ACN69885.1|        RYNKDFCKDIRWSLGDFGDIIMGTDMEGIGYSEVVENNLRSIFGTGEKAQ
gi|224926648|gb|ACN69886.1|        RYNKDFCKDIRWSLGDFGDIIMGTDMEGIGYSKVVENNLRSIFGTGEKAQ
```

gi|224926650|gb|ACN69887.1|	RYNKDFCKDIRWSLGDFGDIIMGTDMEGIGYSEVVENNLRSIFGTGKNAQ
gi|224926652|gb|ACN69888.1|	RYNKDFCKDIRWSLGDFGDIIMGTDMEGIGYSEVVENNLRSIFGTGEKAQ
gi|224926654|gb|ACN69889.1|	RYNKDFCKDIRWSLGDFGDIIMGTDMEGIGYSEVVENNLRSIFGTGEKAQ
gi|224926656|gb|ACN69890.1|	RYNKDFCKDIRWSLGDFGDIIMGTDMEGIGYSKVVENNLRSIFGTGEKAQ
gi|224926658|gb|ACN69891.1|	RYNKDFCKDIRWSLGDFGDIIMGTDMEGIGYSKVVENNLRSIFGTGEKAQ
gi|224926660|gb|ACN69892.1|	RYNKDFCKDIRWSLGDFGDIIMGTDMEGIGYSEVVENNLRSIFGTGKNAQ
gi|224926662|gb|ACN69893.1|	RYNKDFCKDIRWSLGDFGDIIMGTDMEGIGYSKVVENNLRSIFGTGEKAQ
gi|224926664|gb|ACN69894.1|	RYNKDFCKDIRWSLGDFGDIIMGTDMEGIGYSKVVENNLRSIFGTGKNAQ
gi|224926666|gb|ACN69895.1|	RYNKDFCKDIRWSLGDFGDIIMGTDMEGIGYSKVVENNLRSIFGTGEKAQ
gi|224926668|gb|ACN69896.1|	RYNKDFCKDIRWSLGDFGDIIMGTDMEGIGYSEVVENNLRSIFGTGKNAQ
gi|224926670|gb|ACN69897.1|	RYNKDFCKDIRWSLGDFGDIIMGTDMEGIGYSKVVENNLRSIFGTGEKAQ
gi|224926672|gb|ACN69898.1|	RYNKDFCKDIRWSLGDFGDIIMGTDMEGIGYSKVVENNLRSIFGTGEKAQ
gi|224926674|gb|ACN69899.1|	RYNKDFCKDIRWSLGDFGDIIMGTDMEGIGYSEVVENNLRSIFGTGKNAQ
gi|224926676|gb|ACN69900.1|	RYNKDFCKDIRWSLGDFGDIIMGTDMEGIGYSEVVENNLRSIFGTGKNAQ
gi|224926678|gb|ACN69901.1|	RYNKDFCKDIRWSLGDFGDIIMGTDMEGIGYSKVVENNLRSIFGTGEKAQ
gi|224926680|gb|ACN69902.1|	RYNKDFCKDIRWSLGDFGDIIMGTDMEGIGYSKVVENNLRSIFGTGKNAQ
gi|224926682|gb|ACN69903.1|	RYNKDFCKDIRWSLGDFGDIIMGTDMEGIGYSKVVENNLRSIFGTGEKAQ
gi|224926684|gb|ACN69904.1|	RYNKDFCKDIRWSLGDFGDIIMGTDMEGIGYSKVVENNLRSIFGTDEKAQ
gi|224926686|gb|ACN69905.1|	RYNKDFCKDIRWSLGDFGDIIMGTDMEGIGYSKVVENNLRSIFGTDEKAQ
gi|224926688|gb|ACN69906.1|	RYNKDFCKDIRWSLGDFGDIIMGTDMEGIGYSKVVENNLRSIFGTGKNAQ
gi|224926690|gb|ACN69907.1|	RYNKDFCKDIRWSLGDFGDIIMGTDMEGIGYSEVVENNLRSIFGTGKNAQ
gi|224926692|gb|ACN69908.1|	RYNKDFCKDIRWSLGDFGDIIMGTDMEGIGYSKVVENNLRSIFGTDEKAQ
gi|224926694|gb|ACN69909.1|	RYNKDFCKDIRWSLGDFGDIIMGTDMEGIGYSKVVENNLRSIFGTGKNAQ
gi|224926696|gb|ACN69910.1|	RYNKDFCKDIRWSLGDFGDIIMGTDMEGIGYSEVVENNLRSIFGTGKNAQ
gi|224926698|gb|ACN69911.1|	RYNKDFCKDIRWSLGDFGDIIMGTDMEGIGYSKVVENNLRSIFGTGEKAQ
gi|224926700|gb|ACN69912.1|	RYNKDFCKDIRWSLGDFGDIIMGTDMEGIGYSKVVENNLRSIFGTGEKAQ
gi|224926702|gb|ACN69913.1|	RYNKDFCKDIRWSLGDFGDIIMGTDMEGIGYSEVVENNLRSIFGTGKNAQ
gi|224926704|gb|ACN69914.1|	RYNKDFCKDIRWSLGDFGDIIMGTDMEGIGYSKVVENNLRSIFGTGEKAQ
gi|224926706|gb|ACN69915.1|	RYNKDFCKDIRWSLGDFGDIIMGTDMEGIGYSEVVENNLRSIFGTGKNAQ
gi|224926708|gb|ACN69916.1|	RYNKDFCKDIRWSLGDFGDIIMGTDMEGIGYSEVVENNLRSIFGTGKNAQ
gi|224926710|gb|ACN69917.1|	RYNKDFCKDIRWSLGDFGDIIMGTDMEGIGYSKVVENNLRSIFGTDEKAQ
gi|224926714|gb|ACN69918.1|	RYNKDFCKDIRWSLGDFGDIIMGTDMEGIGYSKVVENNLRSIFGTDEKAQ
gi|224926716|gb|ACN69919.1|	RYNKDFCKDIRWSLGDFGDIIMGTDMEGIGYSKVVENNLRSIFGTGEKAQ
gi|224926718|gb|ACN69920.1|	RYNKDFCKDIRWSLGDFGDIIMGTDMEGIGYSKVVENNLRSIFGTDEKAQ
gi|224926720|gb|ACN69921.1|	RYNKDFCKDIRWSLGDFGDIIMGTDMEGIGYSKVVENNLRSIFGTDEKAQ
gi|224926722|gb|ACN69922.1|	RYNKDFCKDIRWSLGDFGDIIMGTDMEGIGYSKVVENNLRSIFGTGEKAQ
gi|224926724|gb|ACN69923.1|	RYNKDFCKDIRWSLGDFGDIIMGTDMEGIGYSKVVENNLRSIFGTGEKAQ
gi|224926726|gb|ACN69924.1|	RYNKDFCKDIRWSLGDFGDIIMGTDMEGIGYSEVVENNLRSIFGTGKNAQ
gi|224926728|gb|ACN69925.1|	RYNKDFCKDIRWSLGDFGDIIMGTDMEGIGYSKVVENNLRSIFGTGEKAQ
gi|224926730|gb|ACN69926.1|	RYNKDFCKDIRWSLGDFGDIIMGTDMEGIGYSKVVENNLRSIFGTDEKAQ
gi|224926732|gb|ACN69927.1|	RYNKDFCKDIRWSLGDFGDIIMGTDMEGIGYSEVVENNLRSIFGTGKNAQ
gi|224926734|gb|ACN69928.1|	RYNKDFCKDIRWSLGDFGDIIMGTDMEGIGYSKVVENNLRSIFGTGEKAQ
gi|224926736|gb|ACN69929.1|	RYNKDFCKDIRWSLGDFGDIIMGTDMEGIGYSKVVENNLRSIFGTGEKAQ
gi|224926738|gb|ACN69930.1|	RYNKDFCKDIRWSLGDFGDIIMGTDMEGIGYSEVVENNLRSIFGTGEQAQ
gi|224926740|gb|ACN69931.1|	RYNKDFCKDIRWSLGDFGDIIMGTDMEGIGYSEVVENNLRSIFGTGEQAQ
gi|224926742|gb|ACN69932.1|	RYNKDFCKDIRWSLGDFGDIIMGTDMEGIGYSKVVENNLRSIFGTDEKAQ
gi|224926744|gb|ACN69933.1|	RYNKDFCKDIRWSLGDFGDIIMGTDMEGIGYSEVVENNLRSIFGTGEKAQ
gi|224926746|gb|ACN69934.1|	RYNKDFCKDIRWSLGDFGDIIMGTDMEGIGYSKVVENNLRSIFGTGEKAQ
gi|224926748|gb|ACN69935.1|	RYNKDFCKDIRWSLGDFGDIIMGTDMEGIGYSKVVENNLRSIFGTGEKAQ
gi|224926750|gb|ACN69936.1|	RYNKDFCKDIRWSLGDFGDIIMGTDMEGIGYSKVVENNLRSIFGTGEKAQ
gi|224926752|gb|ACN69937.1|	RYNKDFCKDIRWSLGDFGDIIMGTDMEGIGYSKVVENNLRSIFGTGEKAQ
gi|224926754|gb|ACN69938.1|	RYNKDFCKDIRWSLGDFGDIIMGTDMEGIGYSKVVEDNLRSIFGTGKNAQ
gi|224926756|gb|ACN69939.1|	RYNKDFCKDIRWSLGDFGDIIMGTDMEGIGYSKVVENNLRSIFGTGEKAQ
gi|224926758|gb|ACN69940.1|	RYNKDFCKDIRWSLGDFGDIIMGTDMEGIGYSEVVENNLRSIFGTGEQAQ
gi|224926760|gb|ACN69941.1|	RYNKDFCKDIRWSLGDFGDIIMGTDMEGIGYSKVVEDNLRSIFGTGKNAQ
gi|224926762|gb|ACN69942.1|	RYNKDFCKDIRWSLGDFGDIIMGTDMEGIGYSKVVEDNLRSIFGTGKNAQ
gi|224926764|gb|ACN69943.1|	RYNKDFCKDIRWSLGDFGDIIMGTDMEGIGYSKVVENNLRSIFGTGEKAQ
gi|224926766|gb|ACN69944.1|	RYNKDFCKDIRWSLGDFGDIIMGTDMEGIGYSKVVEDNLRSIFGTGKNAQ
gi|224926768|gb|ACN69945.1|	RYNKDFCKDIRWSLGDFGDIIMGTDMEGIGYSKVVENNLRSIFGTDEKAQ

```
gi|224926770|gb|ACN69946.1|        RYNKDFCKDIRWSLGDFGDIIMGTDMEGIGYSEVVENNLRSIFGTGEQAQ
gi|224926772|gb|ACN69947.1|        RYNKDFCKDIRWSLGDFGDIIMGTDMEGIGYSEVVENNLRSIFGTGKNAQ
gi|224926774|gb|ACN69948.1|        RYNKDFCKDIRWSLGDFGDIIMGTDMEGIGYSKVVENNLRSIFGTDEKAQ
gi|224926776|gb|ACN69949.1|        RYNKDFCKDIRWSLGDFGDIIMGTDMEGIGYSKVVENNLRSIFGTGKNAQ
gi|224926778|gb|ACN69950.1|        RYNKDFCKDIRWSLGDFGDIIMGTDMEGIGYSKVVENNLRSIFGTGKNAQ
gi|224926780|gb|ACN69951.1|        RYNKDFCKDIRWSLGDFGDIIMGTDMEGIGYSKVVENNLRSIFGTGEKAQ
gi|224926782|gb|ACN69952.1|        RYNKDFCKDIRWSLGDFGDIIMGTDMEGIGYSKVVENNLRSIFGTGEKAQ
gi|224926784|gb|ACN69953.1|        RYNKDFCKDIRWSLGDFGDIIMGTDMEGIRYSEVVENILRSIFGTGEQAQ
gi|224926786|gb|ACN69954.1|        RYNKDFCKDIRWSLGDFGDIIMGTDMEGIGYSKVVENNLRSIFGTGEKAQ
gi|224926788|gb|ACN69955.1|        RYNKDFCKDIRWSLGDFGDIIMGTDMEGIGYSKVVENNLRSIFGTGEKAQ
gi|224926790|gb|ACN69956.1|        RYNKDFCKDIRWSLGDFGDIIMGTDMEGIGYSKVVENNLRSIFGTDEKAQ
gi|224926792|gb|ACN69957.1|        RYNKDFCKDIRWSLGDFGDIIMGTDMEGIGYSKVVENNLRSIFGTGEKAQ
gi|224926794|gb|ACN69958.1|        RYNKDFCKDIRWSLGDFGDIIMGTDMEGIGYSKVVENNLRSIFGTGEKAQ
gi|224926796|gb|ACN69959.1|        RYNKDFCKDIRWSLGDFGDIIMGTDMEGIGYSKVVENNLRSIFGTGEKAQ
gi|224926798|gb|ACN69960.1|        RYNKDFCKDIRWSLGDFGDIIMGTDMEGIGYSKVVENNLRSIFGTGEKAQ
gi|224926800|gb|ACN69961.1|        RYNKDFCKDIRWSLGDFGDIIMGTDMEGIGYSKVVENNLRSIFGTDEKAQ
gi|224926802|gb|ACN69962.1|        RYNKDFCKDIRWSLGDFGDIIMGTDMEGIGYSEVVENNLRSIFGTGKNAQ
gi|224926804|gb|ACN69963.1|        RYNKDFCKDIRWSLGDFGDIIMGTDMEGIGYSEVVENNLRSIFGTGKNAQ
gi|224926806|gb|ACN69964.1|        RYNKDFCKDIRWSLGDFGDIIMGTDMEGIGYSKVVENNLRSIFGTGEKAQ
gi|224926808|gb|ACN69965.1|        RYNKDFCKDIRWSLGDFGDIIMGTDMEGIGYSKVVENNLRSIFGTGKNAQ
gi|224926810|gb|ACN69966.1|        RYNKDFCKDIRWSLGDFGDIIMGTDMEGIGYSEVVENNLRSIFGTGKNAQ
gi|224926812|gb|ACN69967.1|        RYNKDFCKDIRWSLGDFGDIIMGTDMEGIGYSEVVENNLRSIFGTGKNAQ
gi|224926814|gb|ACN69968.1|        RYNKDFCKDIRWSLGDFGDIIMGTDMEGIGYSKVVENNLRSIFGTGEKAQ
gi|224926816|gb|ACN69969.1|        KYNKDFCKDIRWSLGDFGDIIMGTDMEGIGYSKVVENNLRSIFGTGEKAQ
gi|224926818|gb|ACN69970.1|        RYNKDFCKDIRWSLGDFGDIIMGTDMEGIGYSKVVENNLRSIFGTGEKAQ
gi|224926820|gb|ACN69971.1|        RYNKDFCKDIRWSLGDFGDIIMGTDMEGIGYSKVVENNLRSIFGTGEKAQ
gi|213868281|gb|ACJ54187.1|        RYNKDFCKDIRWSLGDFGDIIMGTDMEGIGYSKVVENNLRSIFGTGKNAQ
gi|213868283|gb|ACJ54188.1|        RYNKDFCKDIRWSLGDFGDIIMGTDMEGIGYSKVVENNLRSIFGTGKNAQ
gi|213868285|gb|ACJ54189.1|        RYNKDFCKDIRWSLGDFGDIIMGTDMEGIGYSEVVENNLRSIFGTGEKAQ
gi|213868287|gb|ACJ54190.1|        RYNKDFCKDIRWSLGDFGDIIMGTDMEGIGYSEVVENNLRSIFGTGEKAQ
gi|213868289|gb|ACJ54191.1|        RYNKDFCKDIRWSLGDFGDIIMGTDMEGIGYSKVVENNLRSIFGTDEKAQ
gi|213868291|gb|ACJ54192.1|        RYNKDFCKDIRWSLGDFGDIIMGTDMEGIGYSKVVENNLRSIFGTGEKAQ
gi|213868293|gb|ACJ54193.1|        RYNKDFCKDIRWSLGDFGDIIMGTDMEGIGYSKVVENNLRSIFGTDEKAQ
gi|213868295|gb|ACJ54194.1|        RYNKDFCKDIRWSLGDFGDIIMGTDMEGIGYSKVVENNLRSIFGTDEKAQ
gi|213868297|gb|ACJ54195.1|        RYNKDFCKDIRWSLGDFGDIIMGTDMEGIGYSKVVENNLRSIFGTDEKAQ
gi|213868299|gb|ACJ54196.1|        RYNKDFCKDIRWSLGDFGDIIMGTDMEGIGYSKVVENNLRSIFGTDEKAQ
gi|213868301|gb|ACJ54197.1|        RYNKDFCKDIRWSLGDFGDIIMGTDMEGIGYSKVVENNLRSIFGTGEKAQ
                                   :**************************** **:***:  ******.::**

gi|11120233|gb|AAG30847.1|         QHRKQRWNESKAQIWTAMMYSVKKRLKGNFIWICKINVAVNIEPQIYRWI
gi|12407747|gb|AAG53634.1|         QHRKQWWNESKAQIWTAMMYSVKKRLKGNFIWICKINVAVNIEPQIYRWI
gi|18766659|gb|AAL79050.1|AF46     QRRKQWWNESKAQIWTAMMYSVKKRLKGNFIWICKLNVAVNIEPQIYRWI
gi|18766819|gb|AAL79130.1|AF46     QHRKQWWNESKAQIWTAMMYSVKKRLKGNFIWICKINVAVNIEPQIYRWI
gi|63146462|gb|AAY34059.1|         QHRKQWWNESKAQIWTAMMYSVKKRLKGNFIWICKINVAVNIEPQIYRWI
gi|63146616|gb|AAY34136.1|         QRRKQWWNESKAQIWTAMMYSVKKRLKGNFIWICKLNVAVNIEPQIYRWI
gi|11138983|gb|AAG31571.1|         QHRKQRWNESKAQIWTAMMYSVKKRLKGNFIWICKINVAVNIEPQIYRWI
gi|73697858|gb|AAZ81532.1|         QRRKQWWNESKAQIWTAMMYSVKKRLKGKFIWICKINVAVNIEPQIYRRI
gi|73697846|gb|AAZ81526.1|         QHRKQWWNESKAQIWTAMMYSVKKRLKGNFIWICKLNVAVNIEPQIYRWI
gi|12004297|gb|AAG43989.1|         QHRKQWWNESKAQIWTAMMYSVKKRLKGKFIWICKINVAVNIEPQIYRRI
gi|12004299|gb|AAG43990.1|         QRRKQWWNESKAQIWTAMMYSVKKRLKGKFIWICKINVAVNIEPQIYRRI
gi|73697866|gb|AAZ81536.1|         QRHKQWWNESKAQIWTAMMYSVKKRLKGKFIWICKINVAVNIEPQIYRRI
gi|73697848|gb|AAZ81527.1|         QRRKQWWNESKAQIWTAMMYSVKKRLKGNFIWICKLNVAVNIEPQIYRWI
gi|73697856|gb|AAZ81531.1|         QHRKQWWNESKAQIWTAMMYSVKKRLKGKFIWICKINVAVNIEPQIYRRI
gi|73697862|gb|AAZ81534.1|         QHRKQWWNETKAQIWRAMMYSVKKRLKGNFIWICKINVAVNIEPQIYRWI
gi|160276|gb|AAA63423.1|           QRRKQWWNESKAQIWTAMMYSVKKRLKGNFIWICKLNVAVNIEPQIYRWI
gi|73697844|gb|AAZ81525.1|         QRRKQWWNESKAQIWTAMMYSVKKRLKGNFIWICKLNVAVNIEPQIYRWI
gi|339961368|pdb|3RRC|A            QRRKQWWNESKAQIWTAMMYSVKKRLKGNFIWICKLNVAVNIEPQIYRWI
gi|339961369|pdb|3RRC|B            QRRKQWWNESKAQIWTAMMYSVKKRLKGNFIWICKLNVAVNIEPQIYRWI
gi|255914751|dbj|BAH96577.1|       QHRKQWWNESKAQIWTAMMYSVKKRLKGNFIWICKINVAVNIEPQIYRWI
```

gi|224926624|gb|ACN69874.1|	QRRKQWWNESKAQIWTAMMYSVKKRLKGKFIWICKINVAVNIEPQIYRRI
gi|224926626|gb|ACN69875.1|	QRRKQWWNESKAQIWTAMMYSVKKRLKGKFMWICKINVAVNIEPQIYRRI
gi|224926628|gb|ACN69876.1|	QHRKQWWNESKAQIWTAMMYSVKKRLKGNFIWICKLNVAVNIEPQIYRWI
gi|224926630|gb|ACN69877.1|	QHRKQWWNETKAQIWRAMMYSVKKRLKGNFIWICKINVAVNIEPQIYRWI
gi|224926632|gb|ACN69878.1|	QHRKQWWNESKAQIWTAMMYSVKKRLKGNFIWICKLNVAVNIEPQIYRWI
gi|224926634|gb|ACN69879.1|	QRRKQWWNESKAQIWTAMMYSVKKRLKGKFIWICKINVAVNIEPQIYRRI
gi|224926636|gb|ACN69880.1|	QRRKQWWNESKAQIWTAMMYSVKKRLKGKFMWICKINVAVNIEPQIYRRI
gi|224926638|gb|ACN69881.1|	QHRKQWWNESKAQIWTAMMYSVKKRLKGNFIWICKLNVAVNIEPQIYRWI
gi|224926640|gb|ACN69882.1|	QHRKQWWNESKAQIWTAMMYSVKKRLKGKFIWICKINVAVNIEPQIYRRI
gi|224926642|gb|ACN69883.1|	QRRKQWWNESKAQIWTAMMYSVKKRLKGKFMWICKINVAVNIEPQIYRRI
gi|224926644|gb|ACN69884.1|	QRRKQWWNESKAQIWTAMMYSVKKRLKGKFMWICKINVAVNIEPQIYRRI
gi|224926646|gb|ACN69885.1|	QRRKQWWNESKAQIWTAMMYSVKKRLKGKFIWICKINVAVNIEPQIYRRI
gi|224926648|gb|ACN69886.1|	QHRKQWWNESKAQIWTAMMYSVKKRLKGNFIWICKLNVAVNIEPQIYRWI
gi|224926650|gb|ACN69887.1|	QRRKQWWNESKAQIWTAMMYSVKKRLKGKFIWICKINVAVNIEPQIYRRI
gi|224926652|gb|ACN69888.1|	QRRKQWWNESKAQIWTAMMYSVKKRLKGKFIWICKINVAVNIEPQIYRRI
gi|224926654|gb|ACN69889.1|	QRRKQWWNESKAQIWTAMMYSVKKRLKGKFMWICKINVAVNIEPQIYRRI
gi|224926656|gb|ACN69890.1|	QHRKQWWNESKAQIWTAMMYSVKKRLKGNFIWICKLNVAVNIEPQIYRWI
gi|224926658|gb|ACN69891.1|	QHRKQWWNESKAQIWTAMMYSVKKRLKGNFIWICKLNVAVNIEPQIYRWI
gi|224926660|gb|ACN69892.1|	QHRKQWWNESKAQIWTAMMYSVKKRLKGKFIWICKINVAVNIEPQIYRRI
gi|224926662|gb|ACN69893.1|	QHRKQWWNESKAQIWTAMMYSVKKRLKGNFIWICKLNVAVNIEPQIYRWI
gi|224926664|gb|ACN69894.1|	QHRKQWWNETKAQIWRAMMYSVKKRLKGNFIWICKINVAVNIEPQIYRWI
gi|224926666|gb|ACN69895.1|	QHRKQWWNESKAQIWTAMMYSVKKRLKGNFIWICKLNVAVNIEPQIYRWI
gi|224926668|gb|ACN69896.1|	QRRKQWWNESKAQIWTAMMYSVKKRLKGKFIWICKINVAVNIEPQIYRRI
gi|224926670|gb|ACN69897.1|	QHRKQWWNESKAQIWTAMMYSVKKRLKGNFIWICKLNVAVNIEPQIYRWI
gi|224926672|gb|ACN69898.1|	QHRKQWWNESKAQIWTAMMYSVKKRLKGNFIWICKLNVAVNIEPQIYRWI
gi|224926674|gb|ACN69899.1|	QHRKQWWNESKAQIWTAMMYSVKKRLKGKFIWICKINVAVNIEPQIYRRI
gi|224926676|gb|ACN69900.1|	QHRKQWWNESKAQIWTAMMYSVKKRLKGKFIWICKINVAVNIEPQIYRRI
gi|224926678|gb|ACN69901.1|	QHRKQWWNESKAQIWTAMMYSVKKRLKGNFIWICKLNVAVNIEPQIYRWI
gi|224926680|gb|ACN69902.1|	QHRKQWWNETKAQIWRAMMYSVKKRLKGNFIWICKINVAVNIEPQIYRWI
gi|224926682|gb|ACN69903.1|	QHRKQWWNESKAQIWTAMMYSVKKRLKGNFIWICKLNVAVNIEPQIYRWI
gi|224926684|gb|ACN69904.1|	QRCKQWWNESKAQIWTAMMYSVKKRLKGNFIWICKLNVAVNIEPQIYRWI
gi|224926686|gb|ACN69905.1|	QRRKQWWNESKAQIWTAMMYSVKKRLKGNFIWICKLNVAVNIEPQIYRWI
gi|224926688|gb|ACN69906.1|	QHRKQWWNETKAQIWRAMMYSVKKRLKGNFIWICKINVAVNIEPQIYRWI
gi|224926690|gb|ACN69907.1|	QHRKQWWNESKAQIWTAMMYSVKKRLKGKFIWICKINVAVNIEPQIYRRI
gi|224926692|gb|ACN69908.1|	QRRKQWWNESKAQIWTAMMYSVKKRLKGNFIWICKLNVAVNIEPQIYRWI
gi|224926694|gb|ACN69909.1|	QRCKQWWNEFKAQIWTAMMYSVKKRLKGKFIWICKINVAVNIEPQIYRRI
gi|224926696|gb|ACN69910.1|	QRCKQWWNESKAQIWTAMMYSVKKRLKGKFIWICKINVAVNIEPQIYRRI
gi|224926698|gb|ACN69911.1|	QHCKQWWNESKAQIWTAMMYSVKKRLKGNFIWICKLNVAVNIEPQIYRWI
gi|224926700|gb|ACN69912.1|	QHCKQWWNESKAQIWTAMMYSVKKRLKGNFIWICKLNVAVNIEPQIYRWI
gi|224926702|gb|ACN69913.1|	QHRKQWWNESKAQIWTAMMYSVKKRLKGKFIWICKINVAVNIEPQIYRRI
gi|224926704|gb|ACN69914.1|	QHRKQWWNESKAQIWTAMMYSVKKRLKGNFIWICKLNVAVNIEPQIYRWI
gi|224926706|gb|ACN69915.1|	QHRKQWWNESKAQIWTAMMYSVKKRLKGKFMWICKINVAVNIEPQIYRRI
gi|224926708|gb|ACN69916.1|	QHRKQWWNESKAQIWTAMMYSVKKRLKGKFIWICKINVAVNIEPQIYRRI
gi|224926710|gb|ACN69917.1|	QRRKQWWNESKAQIWTAMMYSVKKRLKGNFIWICKLNVAVNIEPQIYRWI
gi|224926714|gb|ACN69918.1|	QRRKQWWNESKAQIWTAMMYSVKKRLKGNFIWICKLNVAVNIEPQIYRWI
gi|224926716|gb|ACN69919.1|	QHRKQWWNESKAQIWTAMMYSVKKRLKGNFIWICKLNVAVNIEPQIYRWI
gi|224926718|gb|ACN69920.1|	QRRKQWWNESKAQIWTAMMYSVKKRLKGNFIWICKLNVAVNIEPQIYRWI
gi|224926720|gb|ACN69921.1|	QRRKQWWNESKAQIWTAMMYSVKKRLKGNFIWICKLNVAVNIEPQIYRWI
gi|224926722|gb|ACN69922.1|	QHRKQWWNESKAQIWTAMMYSVKKRLKGNFIWICKLNVAVNIEPQIYRWI
gi|224926724|gb|ACN69923.1|	QHRKQWWNESKAQIWTAMMYSVKKRLKGNFIWICKLNVAVNIEPQIYRWI
gi|224926726|gb|ACN69924.1|	QHRKQWWNESKAQIWTAMMYSVKKRLKGKFIWICKINVAVNIEPQIYRRI
gi|224926728|gb|ACN69925.1|	QHRKQWWNESKAQIWTAMMYSVKKRLKGNFIWICKLNVAVNIEPQIYRWI
gi|224926730|gb|ACN69926.1|	QRRKQWWNESKAQIWTAMMYSVKKRLKGNFIWICKLNVAVNIEPQIYRWI
gi|224926732|gb|ACN69927.1|	QHRKQWWNESKAQIWTAMMYSVKKRLKGKFIWICKINVAVNIEPQIYRRI
gi|224926734|gb|ACN69928.1|	QHRKQWWNESKAQIWTAMMYSVKKRLKGNFIWICKLNVAVNIEPQIYRWI
gi|224926736|gb|ACN69929.1|	QHRKQWWNESKAQIWTAMMYSVKKRLKGNFIWICKINVAVNIEPQIYRWI
gi|224926738|gb|ACN69930.1|	QRRKQWWNESKAQIWTAMMYSVKKRLKGKFIWICKINVAVNIEPQIYRRI
gi|224926740|gb|ACN69931.1|	QRRKQWWNESKAQIWTAMMYSVKKRLKGKFIWICKLNVAVNIEPQIYRRI
gi|224926742|gb|ACN69932.1|	QRRKQWWNESKAQIWTAMMYSVKKRLKGNFIWICKLNVAVNTEPQIYRWI

```
gi|224926744|gb|ACN69933.1|         QRRKQWWNESKAQIWTAMMYSVKKRLKGKFMWICKINVAVNIEPQIYRRI
gi|224926746|gb|ACN69934.1|         QHRKQWWNESKAQIWTAMMYSVKKRLKGNFIWICKLNVAVNIEPQIYRWI
gi|224926748|gb|ACN69935.1|         QHRKQWWNESKAQIWTAMMYSVKKRLKGNFIWICKLNVAVNIEPQIYRWI
gi|224926750|gb|ACN69936.1|         QHRKQWWNESKAQIWTAMMYSVKKRLKGNFIWICKLNVAVNIEPQIYRWI
gi|224926752|gb|ACN69937.1|         QHRKQWWNESKAQIWTAMMYSVKKRLKGNFIWICKLNVAVNIEPQIYRWI
gi|224926754|gb|ACN69938.1|         QHRKQWWNESKAQIWTAMMYSVKKRLKGKFIWICKINVAVNIEPQIYRRI
gi|224926756|gb|ACN69939.1|         QHRKQWWNESKAQIWTAMMYSVKKRLKGNFIWICKLNVAVNIEPQIYRWI
gi|224926758|gb|ACN69940.1|         QRCKQWWNEFKAQIWTAMMYSVKKRLKGKFIWICKINVAVNIEPQIYRRI
gi|224926760|gb|ACN69941.1|         QHRKQWWNESKAQIWTAMMYSVKKRLKGKFIWICKINVAVNIEPQIYRRI
gi|224926762|gb|ACN69942.1|         QHRKQWWNESKAQIWTAMMYSVKKRLKGKFIWICKINVAVNIEPQIYRRI
gi|224926764|gb|ACN69943.1|         QHRKQWWNESKAQIWTAMMYSVKKRLKGNFIWICKLNVAVNIEPQIYRWI
gi|224926766|gb|ACN69944.1|         QHRKQWWNESKAQIWTAMMYSVKKRLKGKFIWICKINVAVNIEPQIYRRI
gi|224926768|gb|ACN69945.1|         QRRKQWWNESKAQIWTAMMYSVKKRLKGNFIWICKLNVAVNIEPQIYRWI
gi|224926770|gb|ACN69946.1|         QRRKQWWNESKAQIWTAMMYSVKKRLKGKFIWICKINVAVNIEPQIYRRI
gi|224926772|gb|ACN69947.1|         QRRKQWWNESKAQIWTAMMYSVKKRLKGKFIWICKINVAVNIEPQIYRRI
gi|224926774|gb|ACN69948.1|         QRRKQWWNESKAQIWTAMMYSVKKRLKGNFIWICKLNVAVNIEPQIYRWI
gi|224926776|gb|ACN69949.1|         QHRKQWWNETKAQIWRAMMYSVKKRLKGNFIWICKINVAVNIEPQIYRWI
gi|224926778|gb|ACN69950.1|         QHRKQWWNETKAQIWRAMMYSVKKRLKGNFIWICKINVAVNIEPQIYRWI
gi|224926780|gb|ACN69951.1|         QHRKQWWNESKAQIWTAMMYSVKKRLKGNFIWICKLNVAVNIEPQIYRWI
gi|224926782|gb|ACN69952.1|         QHRKQWWNESKAQIWTAMMYSVKKRLKGNFIWICKLNVAVNIEPQIYRWI
gi|224926784|gb|ACN69953.1|         QRRKQWWNESKAQIWTAMMYSVKKRLKGKFIWICKINVAVNIDPQIYRRF
gi|224926786|gb|ACN69954.1|         QHRKQWWNESKAQIWTAMMYSVKKRLKGNFIWICKLNVAVNIEPQIYRWI
gi|224926788|gb|ACN69955.1|         QHRKQWWNESKAQIWTAMMYSVKKRLKGNFIWICKLNVAVNIEPQIYRWI
gi|224926790|gb|ACN69956.1|         QRRKQWWNESKAQIWTAMMYSVKKRLKGNFIWICKLNVAVNIEPQIYRWI
gi|224926792|gb|ACN69957.1|         QHRKQWWNESKAQIWTAMMYSVKKRLKGNFIWICKLNVAVNIEPQIYRWI
gi|224926794|gb|ACN69958.1|         QHRKQWWNESKAQIWTAMMYSVKKRLKGNFIWICKLNVAVNIEPQIYRWI
gi|224926796|gb|ACN69959.1|         QHRKQWWNESKAQIWTAMMYSVKKRLKGNFIWICKLNVAVNIEPQIYRWI
gi|224926798|gb|ACN69960.1|         QHRKQWWNESKAQIWTAMMYSVKKRLKGNFIWICKLNVAVNIEPQIYRWI
gi|224926800|gb|ACN69961.1|         QRRKQWWNESKAQIWTAMMYSVKKRLKGNFIWICKLNVAVNIEPQIYRWI
gi|224926802|gb|ACN69962.1|         QHRKQWWNESKAQIWTAMMYSVKKRLKGKFIWICKINVAVNIEPQIYRRI
gi|224926804|gb|ACN69963.1|         QRRKQWWNESKAQIWTAMMYSVKKRLKGKFIWICKINVAVNIEPQIYRRI
gi|224926806|gb|ACN69964.1|         QHRKQWWNESKAQIWTAMMYSVKKRLKGNFIWICKLNVAVNIEPQIYRWI
gi|224926808|gb|ACN69965.1|         QHRKQWWNETKAQIWRAMMYSVKKRLKGNFIWICKINVAVNIEPQIYRWI
gi|224926810|gb|ACN69966.1|         QRRKQWWNESKAQIWTAMMYSVKKRLKGKFIWICKINVAVNIEPQIYRRI
gi|224926812|gb|ACN69967.1|         QHRKQWWNESKAQIWTAMMYSVKKRLKGKFIWICKINVAVNIEPQIYRRI
gi|224926814|gb|ACN69968.1|         QHRKQWWNESKAQIWTAMMYSVKKRLKGNFIWICKLNVAVNIEPQIYRWI
gi|224926816|gb|ACN69969.1|         QHRKQWWNESKAQIWTAMMYSVKKRLKGNFIWICKLNVAVNIEPQIYRWI
gi|224926818|gb|ACN69970.1|         QHRKQWWNESKAQIWTAMMYSVKKRLKGNFIWICKLNVAVNIEPQIYRWI
gi|224926820|gb|ACN69971.1|         QHRKQWWNESKAQIWTAMMYSVKKRLKGNFIWICKLNVAVNIEPQIYRWI
gi|213868281|gb|ACJ54187.1|         QHRKQWWNETKAQIWRAMMYSVKKRLKGNFIWICKINVAVNIEPQIYRWI
gi|213868283|gb|ACJ54188.1|         QHRKQWWNETKAQIWRAMMYSVKKRLKGNFIWICKINVAVNIEPQIYRWI
gi|213868285|gb|ACJ54189.1|         QRRKQWWNESKAQIWTAMMYSVKKRLKGKFMWICKINVAVNIEPQIYRRI
gi|213868287|gb|ACJ54190.1|         QRRKQWWNESKAQIWTAMMYSVKKRLKGKFIWICKINVAVNIEPQIYRRI
gi|213868289|gb|ACJ54191.1|         QRRKQWWNESKAQIWTAMMYSVKKRLKGKFIWICKINVAVNIEPQIYRRI
gi|213868291|gb|ACJ54192.1|         QHRKQWWNESKAQIWTAMMYSVKKRLKGNFIWICKLNVAVNIEPQIYRWI
gi|213868293|gb|ACJ54193.1|         QRRKQWWNESKAQIWTAMMYSVKKRLKGNFIWICKLNVAVNIEPQIYRWI
gi|213868295|gb|ACJ54194.1|         QRRKQWWNESKAQIWTAMMYSVKKRLKGNFIWICKLNVAVNIEPQIYRRI
gi|213868297|gb|ACJ54195.1|         QRRKQWWNESKAQIWTAMMYSVKKRLKGNFIWICKLNVAVNIEPQIYRWI
gi|213868299|gb|ACJ54196.1|         QRRKQWWNESKAQIWTAMMYSVKKRLKGKFIWICKINVAVNIEPQIYRRI
gi|213868301|gb|ACJ54197.1|         QHRKQWWNESKAQIWTAMMYSVKKRLKGNFIWICKLNVAVNIEPQIYRWI
                                    *: ** *** ***** ************:*:****:***** :***** :

gi|11120233|gb|AAG30847.1|          REWGRDYVKELPTEVQKLKEKCDGKINYTDKKVCKV-PPCQNACKSYDQW
gi|12407747|gb|AAG53634.1|          REWGRDYVKELPTEVQKLKEKCDGKINYPDKKVCKV-PPCQNACKSYDQW
gi|18766659|gb|AAL79050.1|AF46      REWGRDYVSELPTEVQKLKEKCDGKINYTDKKVCKV-PPCQNACKSYDQW
gi|18766819|gb|AAL79130.1|AF46      REWGRDYVKELPTEVQKLKEKCDGKINYTDKKVCKV-PPCQNACKSYDQW
gi|63146462|gb|AAY34059.1|          REWGRDYVKELPTEVQKLKEKCDGKINYTDKKVCKV-PPCQNACKSYDQW
gi|63146616|gb|AAY34136.1|          REWGRDYVSELPTEVQKLKEKCDGKINYTDKKVCKV-PPCQNACKSYDQW
gi|11138983|gb|AAG31571.1|          REWGRDYVKELPTEVQKLKEKCDGKINYTDKKVCKV-PPCQNACKSYDQW
```

```
gi|73697858|gb|AAZ81532.1|          REWGRDYVSELPTEVQKLKEKCDGKINYTDKKVCKV-PPCQNACKSYDQW
gi|73697846|gb|AAZ81526.1|          REWGRDYVSELPTEVQKLKEKCDGKFNYTDKKVCKV-PPCQNACKSYDQW
gi|12004297|gb|AAG43989.1|          REWGRDYVSELPTEVQKLKEKCDGKINYTDKKVCKV-PPCQNACKSYDQW
gi|12004299|gb|AAG43990.1|          REWGRDYVSELPTEVQKLKEKCDGKINYTDKKVCKV-PPCQNACKSYDQW
gi|73697866|gb|AAZ81536.1|          REWGRDYVSELPTEVQKLKEKCDGKINYTDKKVCKV-PPCQNACKSYDQW
gi|73697848|gb|AAZ81527.1|          REWGRDYVSELPTEVQKLKEKCDGKINYTDKKVCKV-PPCQNACKSYDQW
gi|73697856|gb|AAZ81531.1|          REWGRDYVSELPTEVQKLKEKCDGKINYTDKKVCKV-PPCQNACKSYDQW
gi|73697862|gb|AAZ81534.1|          REWGRDYVSELPTEVQKLKEKCDGKINYTDKKVCKVLPPCQNACKSYDQW
gi|160276|gb|AAA63423.1|            REWGRDYVSELPTEVQKLKEKCDGKINYTDKKVCKV-PPCQNACKSYDQW
gi|73697844|gb|AAZ81525.1|          REWGRDYVSELPTEVQKLKEKCDGKINYTDKKVCKV-PPCQNACKSYDQW
gi|339961368|pdb|3RRC|A             REWGRDYVSELPTEVQKLKEKCDGKINYTDKKVCKV-PPCQNACKSYDQW
gi|339961369|pdb|3RRC|B             REWGRDYVSELPTEVQKLKEKCDGKINYTDKKVCKV-PPCQNACKSYDQW
gi|255914751|dbj|BAH96577.1|        REWGRDYVSELPTEVQKLKEKCDGKINYTDKKVCKV-PPCQNACKSYDQW
gi|224926624|gb|ACN69874.1|         REWGRDYVSELPTEVQKLKEKCDGKINYTDKKVCKV-PPCQNACKSYDQW
gi|224926626|gb|ACN69875.1|         REWGRDYVSELPTEVQKLKEKCDGKINYTDKKVCKV-PPCQNACKSYDQW
gi|224926628|gb|ACN69876.1|         REWGRDYVSELPTEVQKLKEKCDGKINYTDKKVCKV-PPCQNACKSYDQW
gi|224926630|gb|ACN69877.1|         REWGRDYVSELPTEVQKLKEKCDGKINYTDKKVCKVLPPCQNACKSYDQW
gi|224926632|gb|ACN69878.1|         REWGRDYVSELPTEVQKLKEKCDGKINYTDKKVCKV-PPCQNACKSYDQW
gi|224926634|gb|ACN69879.1|         REWGRDYVSELPTEVQKLKEKCDGKINYTDKKVCKV-PPCQNACKSYDQW
gi|224926636|gb|ACN69880.1|         REWGRDYVSELPTEVQKLKEKCDGKINYTDKKVCKV-PPCQNACKSYDQW
gi|224926638|gb|ACN69881.1|         REWGRDYVSELPTEVQKLKEKCDGKINYTDKKVCKV-PPCQNACKSYDQW
gi|224926640|gb|ACN69882.1|         REWGRDYVSELPTEVQKLKEKCDGKINYTDKKVCKV-PPCQNACKSYDQW
gi|224926642|gb|ACN69883.1|         REWGRDYVSELPTEVQKLKEKCDGKINYTDKKVCKV-PPCQNACKSYDQW
gi|224926644|gb|ACN69884.1|         REWGRDYVSELPTEVQKLKEKCDGKINYTDKKVCKV-PPCQNACKSYDQW
gi|224926646|gb|ACN69885.1|         REWGRDYVSELPTEVQKLKEKCDGKINYTDKKVCKV-PPCQNACKSYDQW
gi|224926648|gb|ACN69886.1|         REWGRDYVSELPTEVQKLKEKCDGKINYTDKKVCKV-PPCQNACKSYDQW
gi|224926650|gb|ACN69887.1|         REWGRDYVSELPTEVQKLKEKCDGKINYTDKKVCKV-PPCQNACKSYDQW
gi|224926652|gb|ACN69888.1|         REWGRDYVSELPTEVQKLKEKCDGKINYTDKKVCKV-PPCQNACKSYDQW
gi|224926654|gb|ACN69889.1|         REWGRDYVSELPTEVQKLKEKCDGKINYTDKKVCKV-PPCQNACKSYDQW
gi|224926656|gb|ACN69890.1|         REWGRDYVSELPTEVQKLKEKCDGKINYTDKKVCKV-PPCQNACKSYDQW
gi|224926658|gb|ACN69891.1|         REWGRDYVSELPTEVQKLKEKCDGKINYTDKKVCKV-PPCQNACKSYDQW
gi|224926660|gb|ACN69892.1|         REWGRDYVSELPTEVQKLKEKCDGKINYTDKKVCKV-PPCQNACKSYDQW
gi|224926662|gb|ACN69893.1|         REWGRDYVSELPTEVQKLKEKCDGKINYTDKKVCKV-PPCQNACKSYDQW
gi|224926664|gb|ACN69894.1|         REWGRDYVSELPTEVQKLKEKCDGKINYTDKKVCKVLPPCQNACKSYDQW
gi|224926666|gb|ACN69895.1|         REWGRDYVSELPTEVQKLKEKCDGKINYTDKKVCKV-PPCQNACKSYDQW
gi|224926668|gb|ACN69896.1|         REWGRDYVSELPTEVQKLKEKCDGKINYTDKKVCKV-PPCQNACKSYDQW
gi|224926670|gb|ACN69897.1|         REWGRDYVSELPTEVQKLKEKCDGKINYTDKKVCKV-PPCQNACKSYDQW
gi|224926672|gb|ACN69898.1|         REWGRDYVSELPTEVQKLKEKCDGKINYTDKKVCKV-PPCQNACKSYDQW
gi|224926674|gb|ACN69899.1|         REWGRDYVSELPTEVQKLKEKCDGKINYTDKKVCKV-PPCQNACKSYDQW
gi|224926676|gb|ACN69900.1|         REWGRDYVSELPTEVQKLKEKCDGKINYTDKKVCKV-PPCQNACKSYDQW
gi|224926678|gb|ACN69901.1|         REWGRDYVSELPTEVQKLKEKCDGKINYTDKKVCKV-PPCQNACKSYDQW
gi|224926680|gb|ACN69902.1|         REWGRDYVSELPTEVQKLKEKCDGKINYTDKKVCKVLPPCQNACKSYDQW
gi|224926682|gb|ACN69903.1|         REWGRDYVSELPTEVQKLKEKCDGKINYTDKKVCKV-PPCQNACKSYDQW
gi|224926684|gb|ACN69904.1|         REWGRDYVSELPTEVQKLKEKCDGKINYTDKKVCKV-PPCQNACKSYDQW
gi|224926686|gb|ACN69905.1|         REWGRDYVSELPTEVQKLKEKCDGKINYTDKKVCKV-PPCQNACKSYDQW
gi|224926688|gb|ACN69906.1|         REWGRDYVSELPTEVQKLKEKCDGKINYTDKKVCKVLPPCQNACKSYDQW
gi|224926690|gb|ACN69907.1|         REWGRDYVSELPTEVQKLKEKCDGKINYTDKKVCKV-PPCQNACKSYDQW
gi|224926692|gb|ACN69908.1|         REWGRDYVSELPTEVQKLKEKCDGKINYTDKKVCKV-PPCQNACKSYDQW
gi|224926694|gb|ACN69909.1|         REWGRDYVSELPTEVQKLKEKCDGKINYTDKKVCKV-PPCQNACKSYDQW
gi|224926696|gb|ACN69910.1|         REWGRDYVSELPTEVQKLKEKCDGKINYTDKKVCKV-PPCQNACKSYDQW
gi|224926698|gb|ACN69911.1|         REWGRDYVSELPTEVQKLKEKCDGKINYTDKKVCKV-PPCQNACKSYDQW
gi|224926700|gb|ACN69912.1|         REWGRDYVSELPTEVQKLKEKCDGKINYTDKKVCKV-PPCQNACKSYDQW
gi|224926702|gb|ACN69913.1|         REWGRDYVSELPTEVQKLKEKCDGKINYTDKKVCKV-PPCQNACKSYDQW
gi|224926704|gb|ACN69914.1|         REWGRDYVSELPTEVQKLKEKCDGKINYTDKKVCKV-PPCQNACKSYDQW
gi|224926706|gb|ACN69915.1|         REWGRDYVSELPTEVQKLKEKCDGKINYTDKKVCKV-PPCQNACKSYDQW
gi|224926708|gb|ACN69916.1|         REWGRDYVSELPTEVQKLKEKCDGKINYTDKKVCKV-PPCQNACKSYDQW
gi|224926710|gb|ACN69917.1|         REWGRDYVSELPTEVQKLKEKCDGKINYTDKKVCKV-PPCQNACKSYDQW
gi|224926714|gb|ACN69918.1|         REWGRDYVSELPTEVQKLKEKCDGKINYTDKKVCKV-PPCQNACKSYDQW
gi|224926716|gb|ACN69919.1|         REWGRDYVSELPTEVQKLKEKCDGKINYTDKKVCKV-PPCQNACKSYDQW
```

```
gi|224926718|gb|ACN69920.1|         REWGRDYVSELPTEVQKLKEKCDGKINYTDKKVCKV-PPCQNACKSYDQW
gi|224926720|gb|ACN69921.1|         REWGRDYVSELPTEVQKLKEKCDGKINYTDKKVCKV-PPCQNACKSYDQW
gi|224926722|gb|ACN69922.1|         REWGRDYVSELPTEVQKLKEKCDGKINYTDKKVCKV-PPCQNACKSYDQW
gi|224926724|gb|ACN69923.1|         REWGRDYVSELPTEVQKLKEKCDGKINYTDKKVCKV-PPCQNACKSYDQW
gi|224926726|gb|ACN69924.1|         REWGRDYVSELPTEVQKLKEKCDGKINYTDKKVCKV-PPCQNACKSYDQW
gi|224926728|gb|ACN69925.1|         REWGRDYVSELPTEVQKLKEKCDGKINYTDKKVCKV-PPCQNACKSYDQW
gi|224926730|gb|ACN69926.1|         REWGRDYVSELPTEVQKLKEKCDGKINYTDKKVCKV-PPCQNACKSYDQW
gi|224926732|gb|ACN69927.1|         REWGRDYVSELPTEVQKLKEKCDGKINYTDKKVCKV-PPCQNACKSYDQW
gi|224926734|gb|ACN69928.1|         REWGRDYVSELPTEVQKLKEKCDGKINYTDKKVCKV-PPCQNACKSYDQW
gi|224926736|gb|ACN69929.1|         REWGRDYVSELPTEVQKLKEKCDGKINYTDKKVCKV-PPCQNACKSYDQW
gi|224926738|gb|ACN69930.1|         REWGRDYVSELPTEVQKLKEKCDGKINYTDKKVCKV-PPCQNACKSYDQW
gi|224926740|gb|ACN69931.1|         REWGRDYVSELPTEVQKLKEKCDGKINYTDKKVCKV-PPCQNACKSYDQW
gi|224926742|gb|ACN69932.1|         REWGRDYVSELPTEVQKLKEKCDGKINYTDKKVCKV-PPCQNACKSYDQW
gi|224926744|gb|ACN69933.1|         REWGRDYVSELPTEVQKLKEKCDGKINYTDKKVCKV-PPCQNACKSYDQW
gi|224926746|gb|ACN69934.1|         REWGRDYVSELPTEVQKLKEKCDGKINYTDKKVCKV-PPCQNACKSYDEW
gi|224926748|gb|ACN69935.1|         REWGRDYVSELPTEVQKLKEKCDGKINYTDKKVCKV-PPCQNACKSYDQW
gi|224926750|gb|ACN69936.1|         REWGRDYVSELPTEVQKLKEKCDGKINYTDKKVCKV-PPCQNACKSYDQW
gi|224926752|gb|ACN69937.1|         REWGRDYVSELPTEVQKLKEKCDGKINYTDKKVCKV-PPCQNACKSYDQW
gi|224926754|gb|ACN69938.1|         REWGRDYVSELPTEVQKLKEKCDGKINYTDKKVCKV-PPCQNACKSYDQW
gi|224926756|gb|ACN69939.1|         REWGRDYVSELPTEVQKLKEKCDGKINYTDKKVCKV-PPCQNACKSYDEW
gi|224926758|gb|ACN69940.1|         REWGRDYVSELPTEVQKLKEKCDGKINYTDKKVCKV-PPCQNACKSYDQW
gi|224926760|gb|ACN69941.1|         REWGRDYVSELPTEVQKLKEKCDGKINYTDKKVCKV-PPCQNACKSYDQW
gi|224926762|gb|ACN69942.1|         REWGRDYVSELPTEVQKLKEKCDGKINYTDKKVCKV-PPCQNACKSYDQW
gi|224926764|gb|ACN69943.1|         REWGRDYVSELPTEVQKLKEKCDGKINYTDKKVCKV-PPCQNACKSYDQW
gi|224926766|gb|ACN69944.1|         REWGRDYVSELPTEVQKLKEKCDGKINYTDKKVCKV-PPCQNACKSYDQW
gi|224926768|gb|ACN69945.1|         REWGRDYVSELPTEVQKLKEKCDGKINYTDKKVCKV-PPCQNACKSYDQW
gi|224926770|gb|ACN69946.1|         REWGRDYVSELPTEVQKLKEKCDGKINYTDKKVCKV-PPCQNACKSYDQW
gi|224926772|gb|ACN69947.1|         REWGRDYVSELPTEVQKLKEKCDGKINYTDKKVCKV-PPCQNACKSYDQW
gi|224926774|gb|ACN69948.1|         REWGRDYVSELPTEVQKLKEKCDGKINYTDKKVCKV-PPCQNACKSYDQW
gi|224926776|gb|ACN69949.1|         REWGRDYVSELPTEVQKLKEKCDGKINYTDKKVCKVLPPCQNACKSYDQW
gi|224926778|gb|ACN69950.1|         REWGRDYVSELPTEVQKLKEKCDGKINYTDKKVCKVLPPCQNACKSYDEW
gi|224926780|gb|ACN69951.1|         REWGRDYVSELPTEVQKLKEKCDGKINYTDKKVCKV-PPCQNACKSYDQW
gi|224926782|gb|ACN69952.1|         REWGRDYVSELPTEVQKLKEKCDGKINYTDKKVCKV-PPCQNACKSYDQW
gi|224926784|gb|ACN69953.1|         REWGRDYVSELPTEVQKLKEKCDGKINYTDKKVCKV-PPCQNACKSYDQW
gi|224926786|gb|ACN69954.1|         REWGRDYVSELPTEVQKLKEKCDGKINYTDKKVCKV-PPCQNACKSYDQW
gi|224926788|gb|ACN69955.1|         REWGRDYVSELPTEVQKLKEKCDGKINYTDKKVCKV-PPCQNACKSYDQW
gi|224926790|gb|ACN69956.1|         REWGRDYVSELPTEVQKLKEKCDGKINYTDKKVCKV-PPCQNACKSYDQW
gi|224926792|gb|ACN69957.1|         REWGRDYVSELPTEVQKLKEKCDGKINYTDKKVCKV-PPCQNACKSYDQW
gi|224926794|gb|ACN69958.1|         REWGRDYVSELPTEVQKLKEKCDGKINYTDKKVCKV-PPCQNACKSYDQW
gi|224926796|gb|ACN69959.1|         REWGRDYVSELPTEVQKLKEKCDGKINYTDKKVCKV-PPCQNACKSYDQW
gi|224926798|gb|ACN69960.1|         REWGRDYVSELPTEVQKLKEKCDGKINYTDKKVCKV-PPCQNACKSYDQW
gi|224926800|gb|ACN69961.1|         REWGRDYVSELPTEVQKLKEKCDGKINYTDKKVCKV-PPCQNACKSYDQW
gi|224926802|gb|ACN69962.1|         REWGRDYVSELPTEVQKLKEKCDGKINYTDKKVCKV-PPCQNACKSYDQW
gi|224926804|gb|ACN69963.1|         REWGRDYVSELPTEVQKLKEKCDGKINYTDKKVCKV-PPCQNACKSYDQW
gi|224926806|gb|ACN69964.1|         REWGRDYVSELPTEVQKLKEKCDGKINYTDKKVCKV-PPCQNACKSYDQW
gi|224926808|gb|ACN69965.1|         REWGRDYVSELPTEVQKLKEKCDGKINYTDKKVCKVLPPCQNACKSYDQW
gi|224926810|gb|ACN69966.1|         REWGRDYVSELPTEVQKLKEKCDGKINYTDKKVCKV-PPCQNACKSYDQW
gi|224926812|gb|ACN69967.1|         REWGRDYVSELPTEVQKLKEKCDGKINYTDKKVCKV-PPCQNACKSYDQW
gi|224926814|gb|ACN69968.1|         REWGRDYVSELPTEVQKLKEKCDGKINYTDKKVCKV-PPCQNACKSYDQW
gi|224926816|gb|ACN69969.1|         REWGRDYVSELPTEVQKLKEKCDGKINYTDKKVCKV-PPCQNACKSYDQW
gi|224926818|gb|ACN69970.1|         REWGRDYVSELPTEVQKLKEKCDGKINYTDKKVCKV-PPCQNACKSYDQW
gi|224926820|gb|ACN69971.1|         REWGRDYVSELPTEVQKLKEKCDGKINYTDKKVCKV-PPCQNACKSYDQW
gi|213868281|gb|ACJ54187.1|         REWGRDYVSELPTEVQKLKEKCDGKINYTDKKVCKVLPPCQNACKSYDQW
gi|213868283|gb|ACJ54188.1|         REWGRDYVSELPTEVQKLKEKCDGKINYTDKKVCKVLPPCQNACKSYDQW
gi|213868285|gb|ACJ54189.1|         REWGRDYVSELPTEVQKLKEKCDGKINYTDKKVCKV-PPCQNACKSYDQW
gi|213868287|gb|ACJ54190.1|         REWGRDYVSELPTEVQKLKEKCDGKINYTDKKVCKV-PPCQNACKSYDQW
gi|213868289|gb|ACJ54191.1|         REWGRDYVSELPTEVQKLKEKCDGKINYTDKKVCKV-PPCQNACKSYDQW
gi|213868291|gb|ACJ54192.1|         REWGRDYVSELPTEVQKLKEKCDGKINYTDKKVCKV-PPCQNACKSYDEW
gi|213868293|gb|ACJ54193.1|         REWGRDYVSELPTEVQKLKEKCDGKINYTDKKVCKV-PPCQNACKSYDQW
```

```
gi|213868295|gb|ACJ54194.1|         REWGRDYVSELPTEVQKLKEKCDGKINYTDKKVCKV-PPCQNACKSYDQW
gi|213868297|gb|ACJ54195.1|         REWGRDYVSELPTEVQKLKEKCDGKINYTDKKVCKV-PPCQNACKSYDQW
gi|213868299|gb|ACJ54196.1|         REWGRDYVSELPTEVQKLKEKCDGKINYTDKKVCKV-PPCQNACKSYDQW
gi|213868301|gb|ACJ54197.1|         REWGRDYVSELPTEVQKLKEKCDGKINYTDKKVCKV-PPCQNACKSYDEW
                                    *********.***************:**.******* ***********:*

gi|11120233|gb|AAG30847.1|          ITRKKNQWDVLSNKFISVKNAEKVQTAGIVTPYDILKQELDEFNEVAFEN
gi|12407747|gb|AAG53634.1|          ITRKKNQWDVLSNKFISVKNAEKVQTAGIVTPYDILKQELDEFNEVAFEN
gi|18766659|gb|AAL79050.1|AF46     ITRKKNQWDVLSNKFISVKNAEKVQTAGIVTPYD----------------
gi|18766819|gb|AAL79130.1|AF46     ITRKKNQWDVLSNKFISVKNAEKVQTAGIVTPYD----------------
gi|63146462|gb|AAY34059.1|          ITRKKNQWDVLSNKFISVKNAEKVQTAGIVTPYD----------------
gi|63146616|gb|AAY34136.1|          ITRKKNQWDVLSNKFISVKNAEKVQTAGIVTP------------------
gi|11138983|gb|AAG31571.1|          ITRKKNQWDVLSNKFISVKNAEKVQTAGIVTPYDILKQELDEFNEVAFEN
gi|73697858|gb|AAZ81532.1|          ITRKKNQWDVLSNKFKSVKNAEKVQTAGIVTPYDILKQELDEFNEVAFEN
gi|73697846|gb|AAZ81526.1|          ITRKKNQWDVLSNKFISVKNAEKVQTAGIVTPYDILKQELDEFNEVAFEN
gi|12004297|gb|AAG43989.1|          ITRKKNQWDVLSNKFKSVKNAEKVQTAGIVTPYDILKQELDEFNEVAFEN
gi|12004299|gb|AAG43990.1|          ITRKKNQWDVLSNKFKSVKNAEKVQTAGIVTPYDILKQELDEFNEVAFEN
gi|73697866|gb|AAZ81536.1|          ITRKKNQWDVLSNKFKSVKNAEKVQTAGIVTPYDILKQELDEFNEVAFEN
gi|73697848|gb|AAZ81527.1|          ITRKKNQWDVLSNKFISVKNAEKVQTAGIVTPYDILKQELDEFNEVAFEN
gi|73697856|gb|AAZ81531.1|          ITRKKNQWDVLSNKFKSVKNAEKVQTAGIVTPYDILKQELDEFNEVAFEN
gi|73697862|gb|AAZ81534.1|          ITRKKNQWDVLSNKFISVKNAEKVQTAGIVTPYDILKQELDEFNEVAFEN
gi|160276|gb|AAA63423.1|            ITRKKNQWDVLSNKFISVKNAEKVQTAGIVTPYDILKQELDEFNEVAFEN
gi|73697844|gb|AAZ81525.1|          ITRKKNQWDVLSNKFISVKNAEKVQTAGIVTPYDILKQELDEFNEVAFEN
gi|339961368|pdb|3RRC|A             ITRKKNQWDVLSNKFISVKNAEKVQTAGIVTPYDILKQELDEFNEVAFEN
gi|339961369|pdb|3RRC|B             ITRKKNQWDVLSNKFISVKNAEKVQTAGIVTPYDILKQELDEFNEVAFEN
gi|255914751|dbj|BAH96577.1|        ITRKKNQWDVLSNKFKSVKNAEKVQTAGIVTPYDILKQELDEFNEVAFEN
gi|224926624|gb|ACN69874.1|         ITRKKNQWDVLSNKFKSVKNAEKVQTAGIVTPYDILKQELDEFNEVAFEN
gi|224926626|gb|ACN69875.1|         ITRKKNQWDVLSNKFISVKNAEKVQTAGIVTPYDILKQELDEFNEVAFEN
gi|224926628|gb|ACN69876.1|         ITRKKNQWDVLSNKFKSVKNAEKVQTAGIVTPYDILKQELDEFNEVAFEN
gi|224926630|gb|ACN69877.1|         ITRKKNQWDVLSNKFISVKNAEKVQTAGIVTPYDILKQELDEFNEVAFEN
gi|224926632|gb|ACN69878.1|         ITRKKNQWDVLSNKFKSVKNAEKVQTAGIVTPYDILKQELDEFNEVAFEN
gi|224926634|gb|ACN69879.1|         ITRKKNQWDVLSNKFKSVKNAEKVQTAGIVTPYDILKQELDEFNEVAFEN
gi|224926636|gb|ACN69880.1|         ITRKKNQWDVLSNKFISVKNAEKVQTAGIVTPYDILKQELDEFNEVAFEN
gi|224926638|gb|ACN69881.1|         ITRKKNQWDVLSNKFKSVKNAEKVQTAGIVTPYDILKQELDEFNEVAFEN
gi|224926640|gb|ACN69882.1|         ITRKKNQWDVLSNKFISVKNAEKVQTAGIVTPYDILKQELDEFNEVAFEN
gi|224926642|gb|ACN69883.1|         ITRKKNQWDVLSNKFISVKNAEKVQTAGIVTPYDILKQELDEFNEVAFEN
gi|224926644|gb|ACN69884.1|         ITRKKNQWDVLSNKFISVKNAEKVQTAGIVTPYDILKQELDEFNEVAFEN
gi|224926646|gb|ACN69885.1|         ITRKKNQWDVLSNKFKSVKNAEKVQTAGIVTPYDILKQELDEFNEVAFEN
gi|224926648|gb|ACN69886.1|         ITRKKNQWDVLSNKFKSVKNAEKVQTAGIVTPYDILKQELDEFNEVAFEN
gi|224926650|gb|ACN69887.1|         ITRKKNQWDVLSNKFKSVKNAEKVQTAGIVTPYDILKQELDEFNEVAFEN
gi|224926652|gb|ACN69888.1|         ITRKKNQWDVLSNKFKSVKNAEKVQTAGIVTPYDILKQELDEFNEVAFEN
gi|224926654|gb|ACN69889.1|         ITRKKNQWDVLSNKFISVKNAEKVQTAGIVTPYDILKQELDEFNEVAFEN
gi|224926656|gb|ACN69890.1|         ITRKKNQWDVLSNKFKSVKNAEKVQTAGIVTPYDILKQELDEFNEVAFEN
gi|224926658|gb|ACN69891.1|         ITRKKNQWDVLSNKFKSVKNAEKVQTAGIVTPYDILKQELDEFNEVAFEN
gi|224926660|gb|ACN69892.1|         ITRKKNQWDVLSNKFISVKNAEKVQTAGIVTPYDILKQELDEFNEVAFEN
gi|224926662|gb|ACN69893.1|         ITRKKNQWDVLSNKFKSVKNAEKVQTAGIVTPYDILKQELDEFNEVAFEN
gi|224926664|gb|ACN69894.1|         ITRKKNQWDVLSNKFISVKNAEKVQTAGIVTPYDILKQELDEFNEVAFEN
gi|224926666|gb|ACN69895.1|         ITRKKNQWDVLSNKFKSVKNAEKVQTAGIVTPYDILKQELDEFNEVAFEN
gi|224926668|gb|ACN69896.1|         ITRKKNQWDVLSNKFKSVKNAEKVQTAGIVTPYDILKQELDEFNEVAFEN
gi|224926670|gb|ACN69897.1|         ITRKKNQWDVLSNKFKSVKNAEKVQTAGIVTPYDILKQELDEFNEVAFEN
gi|224926672|gb|ACN69898.1|         ITRKKNQWDVLSNKFKSVKNAEKVQTAGIVTPYDILKQELDEFNEVAFEN
gi|224926674|gb|ACN69899.1|         ITRKKNQWDVLSNKFKSVKNAEKVQTAGIVTPYDILKQELDEFNEVAFEN
gi|224926676|gb|ACN69900.1|         ITRKKNQWDVLSNKFKSVKNAEKVQTAGIVTPYDILKQELDEFNEVAFEN
gi|224926678|gb|ACN69901.1|         ITRKKNQWDVLSNKFKSVKNAEKVQTAGIVTPYDILKQELDEFNEVAFEN
gi|224926680|gb|ACN69902.1|         ITRKKNQWDVLSNKFISVKNAEKVQTAGIVTPYDILKQELDEFNEVAFEN
gi|224926682|gb|ACN69903.1|         ITRKKNQWDVLSNKFKSVKNAEKVQTAGIVTPYDILKQELDEFNEVAFEN
gi|224926684|gb|ACN69904.1|         ITRKKNQWDVLSNKFISVKNAEKVQTAGIVTPYDILKQELDEFNEVAFEN
gi|224926686|gb|ACN69905.1|         ITRKKNQWDVLSNKFISVKNAEKVQTAGIVTPYDILKQELDEFNEVAFEN
gi|224926688|gb|ACN69906.1|         ITRKKNQWDVLSNKFISVKNAEKVQTAGIVTPYDILKQELDEFNEVAFEN
```

gi|224926690|gb|ACN69907.1| ITRKKNQWDVLSNKFKSVKNAEKVQTAGIVTPYDILKQELDEFNEVAFEN
gi|224926692|gb|ACN69908.1| ITRKKNQWDVLSNKFISVKNAEKVQTAGIVTPYDILKQELDEFNEVAFEN
gi|224926694|gb|ACN69909.1| VTRKKNQWDVLSNKFKSVKNAEKVQTAGIVTPYDILKQELDEFNEVAFEN
gi|224926696|gb|ACN69910.1| ITRKKNQWDVLSNKFKSVKNAEKVQTAGIVTPYDILKQELDEFNEVAFEN
gi|224926698|gb|ACN69911.1| ITRKKNQWDVLSNKFKSVKNAEKVQTAGIVTPYDILKQELDEFNEVAFEN
gi|224926700|gb|ACN69912.1| VTRKKNQWDVLSNKFKSVKNAEKVQTAGIVTPYDILKQELDEFNEVAFEN
gi|224926702|gb|ACN69913.1| ITRKKNQWDVLSNKFKSVKNAEKVQTAGIVTPYDILKQELDEFNEVAFEN
gi|224926704|gb|ACN69914.1| ITRKKNQWDVLSNKFKSVKNAEKVQTAGIVTPYDILKQELDEFNEVAFEN
gi|224926706|gb|ACN69915.1| ITRKKNQWDVLSNKFISVKNAEKVQTAGIVTPYDILKQELDEFNEVAFEN
gi|224926708|gb|ACN69916.1| ITRKKNQWDVLSNKFKSVKNAEKVQTAGIVTPYDILKQELDEFNEVAFEN
gi|224926710|gb|ACN69917.1| ITRKKNQWDVLSNKFISVKNAEKVQTAGIVTPYDILKQELDEFNEVAFEN
gi|224926714|gb|ACN69918.1| ITRKKNQWDVLSNKFKSVKNAEKVQTAGIVTPYDILKQELDEFNEVAFEN
gi|224926716|gb|ACN69919.1| ITRKKNQWDVLSNKFKSVKNAEKVQTAGIVTPYDILKQELDEFNEVALEN
gi|224926718|gb|ACN69920.1| ITRKKNQWDVLSNKFISVKNAEKVQTAGIVTPYDILKQELDEFNEVAFEN
gi|224926720|gb|ACN69921.1| ITRKKNQWDVLSNKFISVKNAEKVQTAGIVTPYDILKQELDEFNEVAFEN
gi|224926722|gb|ACN69922.1| ITRKKNQWDVLSNKFKSVKNAEKVQTAGIVTPYDILKQELDEFNEVAFEN
gi|224926724|gb|ACN69923.1| ITRKKNQWDVLSNKFISVKNAEKVQTAGIVTPYDILKQELDEFNEVAFEN
gi|224926726|gb|ACN69924.1| ITRKKNQWDVLSNKFKSVKNAEKVQTAGIVTPYDILKQELDEFNEVAFEN
gi|224926728|gb|ACN69925.1| ITRKKNQWDVLSNKFISVKNAEKVQTAGIVTPYDILKQELDEFNEVAFEN
gi|224926730|gb|ACN69926.1| ITRKKNQWDVLSNKFISVKNAEKVQTAGIVTPYDILKQELDEFNEVAFEN
gi|224926732|gb|ACN69927.1| ITRKKNQWDVLSNKFKSVKNAEKVQTAGIVTPYDILKQELDEFNEVAFEN
gi|224926734|gb|ACN69928.1| ITRKKNQWDVLSNKFKSVKNAEKVQTAGIVTPYDILKQELDEFNEVAFEN
gi|224926736|gb|ACN69929.1| ITRKKNQWDVLSNKFISVKNAEKVQTAGIVTPYDILKQELDEFNEVAFEN
gi|224926738|gb|ACN69930.1| ITRKKNQWDVLSNKFKSVKNAEKVQTAGIVTPYDILKQELDEFNEVAFEN
gi|224926740|gb|ACN69931.1| ITRKKNQWDVLSNKFKSVKNAEKVQTAGIVTPYDILKQELDEFNEVAFEN
gi|224926742|gb|ACN69932.1| ITRKKNQWDVLSNKFKSVKNAEKVQTAGIVTPYDILKQELDEFIEVAFEN
gi|224926744|gb|ACN69933.1| ITRKKNQWDVLSNKFISVKNAEKVQTAGIVTPYDILKQELDEFNEVAFEN
gi|224926746|gb|ACN69934.1| ITRKKNQWDVLSNKFISVKNAEKVQTAGIVTPYDILKQELDEFNEVAFEN
gi|224926748|gb|ACN69935.1| ITRKKNQWDVLSNKFKSVKNAEKVQTAGIVTPYDILKQELDEFNEVAFEN
gi|224926750|gb|ACN69936.1| ITRKKNQWDVLSNKFKSVKNAEKVQTAGIVTPYDILKQELDEFNEVAFEN
gi|224926752|gb|ACN69937.1| ITRKKNQWDVLSNKFISVKNAEKVQTAGIVTPYDILKQELDEFNEVAFEN
gi|224926754|gb|ACN69938.1| ITRKKNQWDVLSNKFKSVKNAEKVQTAGIVTPYDILKQELDEFNEVAFEN
gi|224926756|gb|ACN69939.1| ITRKKNQWDVLSNKFISVKNAEKVQTAGIVTPYDILKQELDEFNEVAFEN
gi|224926758|gb|ACN69940.1| ITRKKNQWDVLSNKFKSVKNAEKVQTAGIVTPYDILKQELDEFNEVAFEN
gi|224926760|gb|ACN69941.1| ITRKKNQWDVLSNKFKSVKNAEKVQTAGIVTPYDILKQELDEFNEVAFEN
gi|224926762|gb|ACN69942.1| ITRKKNQWDVLSNKFKSVKNAEKVQTAGIVTPYDILKQELDEFNEVAFEN
gi|224926764|gb|ACN69943.1| ITRKKNQWDVLSNKFKSVKNAEKVQTAGIVTPYDILKQELDEFNEVAFEN
gi|224926766|gb|ACN69944.1| ITRKKNQWDVLSNKFKSVKNAEKVQTAGIVTPYDILKQELDEFNEVAFEN
gi|224926768|gb|ACN69945.1| ITRKKNQWDVLSNKFISVKNAEKVQTAGIVTPYDILKQELDEFNEVAFEN
gi|224926770|gb|ACN69946.1| ITRKKNQWDVLSNKFKSVKNAEKVQTAGIVTPYDILKQELDEFNEVAFEN
gi|224926772|gb|ACN69947.1| ITRKKNQWDVLSNKFISVKNAEKVQTAGIVTPYDILKQELDEFNEVAFEN
gi|224926774|gb|ACN69948.1| ITRKKNQWDVLSNKFISVKNAEKVQTAGIVTPYDILKQELDEFNEVAFEN
gi|224926776|gb|ACN69949.1| ITRKKNQWDVLSNKFISVKNAEKVQTAGIVTPYDILKQELDEFNEMAFEN
gi|224926778|gb|ACN69950.1| ITRKKNQWDVLSNKFISVKNAEKVQTAGIVTPYDILKQELDEFNEVAFEN
gi|224926780|gb|ACN69951.1| ITRKKNQWDVLSNKFKSVKNAEKVQTAGIVTPYDILKQELDEFNEVAFEN
gi|224926782|gb|ACN69952.1| ITRKKNQWDVLSNKFISVKNAEKVQTAGIVTPYDILKQELDEFNEVAFEN
gi|224926784|gb|ACN69953.1| ITRKKNQWDVLSNKFKSVKNAEKVQTAGIVTPYDILKQELDEFNEVAFEN
gi|224926786|gb|ACN69954.1| ITRKKNQWDVLSNKFISVKNAEKVQTAGIVTPYDILKQELDEFNEVAFEN
gi|224926788|gb|ACN69955.1| ITRKKNQWDVLSNKFKSVKNAEKVQTAGIVTPYDILKQELDEFNEVAFEN
gi|224926790|gb|ACN69956.1| ITRKKNQWDVLSNKFISVKNAEKVQTAGIVTPYDILKQELDEFNEVAFEN
gi|224926792|gb|ACN69957.1| ITRKKNQWDVLSNKFISVKNAEKVQTAGIVTPYDILKQELDEFNEVAFEN
gi|224926794|gb|ACN69958.1| ITRKKNQWDVLSNKFISVKNAEKVQTAGIVTPYDILKQELDEFNEVAFEN
gi|224926796|gb|ACN69959.1| ITRKKNQWDVLSNKFKSVKNAEKVQTAGIVTPYDILKQELDEFNEVAFEN
gi|224926798|gb|ACN69960.1| ITRKKNQWDVLSNKFKSVKNAEKVQTAGIVTPYDILKQELDEFNEVAFEN
gi|224926800|gb|ACN69961.1| ITRKKNQWDVLSNKFISVKNAEKVQTAGIVTPYDILKQELDEFNEVAFEN
gi|224926802|gb|ACN69962.1| ITRKKNQWDVLSNKFISVKNAEKVQTAGIVTPYDILKQELDEFNEVAFEN
gi|224926804|gb|ACN69963.1| ITRKKNQWDVLSNKFKSVKNAEKVQTAGIVTPYDILKQELDEFNEVAFEN
gi|224926806|gb|ACN69964.1| ITRKKNQWDVLSNKFKSVKNAEKVQTAGIVTPYDILKQELDEFNEVAFEN
gi|224926808|gb|ACN69965.1| ITRKKNQWDVLSNKFISVKNAEKVQTAGIVTPYDILKQELDEFNEVAFEN

```
gi|224926810|gb|ACN69966.1|        ITRKKNQWDVLSNKFKSVKNAEKVQTAGIVTPYDILKQELDEFNEVAFEN
gi|224926812|gb|ACN69967.1|        ITRKKNQWDVLSNKFISVKNAEKVQTAGIVTPYDILKQELDEFNEVAFEN
gi|224926814|gb|ACN69968.1|        ITRKKNQWDVLSNKFISVKNAEKVQTAGIVTPYDILKQELDEFNEVAFEN
gi|224926816|gb|ACN69969.1|        ITRKKNQWDVLSNKFISVKNAEKVQTAGIVTPYDILKQELDEFNEVAFEN
gi|224926818|gb|ACN69970.1|        ITRKKNQWDVLSNKFISVKNAEKVQTAGIVTPYDILKQELDEFNEVAFEN
gi|224926820|gb|ACN69971.1|        ITRKKNQWDVLSNKFKSVKNAEKVQTAGIVTPYDILKQELDEFNEVAFEN
gi|213868281|gb|ACJ54187.1|        ITRKKNQWDVLSNKFISVKNAEKVQTAGIVTPYDILKQELDEFNEVAFEN
gi|213868283|gb|ACJ54188.1|        ITRKKNQWDVLSNKFISVKNAEKVQTAGIVTPYDILKQELDEFNEVAFEN
gi|213868285|gb|ACJ54189.1|        ITRKKNQWDVLSNKFISVKNAEKVQTAGIVTPYDILKQELDEFNEVAFEN
gi|213868287|gb|ACJ54190.1|        ITRKKNQWDVLSNKFKSVKNAEKVQTAGIVTPYDILKQELDEFNEVAFEN
gi|213868289|gb|ACJ54191.1|        ITRKKNQWDVLSNKFKSVKNAEKVQTAGIVTPYDILKQELDEFNEVAFEN
gi|213868291|gb|ACJ54192.1|        ITRKKNQWDVLSNKFKSVKNAEKVQTAGIVTPYDILKQELDEFNEVAFEN
gi|213868293|gb|ACJ54193.1|        ITRKKNQWDVLSNKFKSVKNAEKVQTAGIVTPYDILKQELDEFNEVAFEN
gi|213868295|gb|ACJ54194.1|        ITRKKNQWDVLSNKFKSVKNAEKVQTAGIVTPYDILKQELDEFNEVAFEN
gi|213868297|gb|ACJ54195.1|        ITRKKNQWDVLSNKFISVKNAEKVQTAGIVTPYDILKQELDEFNEVAFEN
gi|213868299|gb|ACJ54196.1|        ITRKKNQWDVLSNKFKSVKNAEKVQTAGIVTPYDILKQELDEFNEVAFEN
gi|213868301|gb|ACJ54197.1|        ITRKKNQWDVLSNKFKSVKNAEKVQTAGIVTPYDILKQELDEFNEVAFEN
                                   :************** ****************

gi|11120233|gb|AAG30847.1|         EINKRDGAYIELCVCSVEEAKKNTQEVVT
gi|12407747|gb|AAG53634.1|         EINKRDGAYIELCVCSVEEAKKNTQEVVT
gi|18766659|gb|AAL79050.1|AF46     -----------------------------
gi|18766819|gb|AAL79130.1|AF46     -----------------------------
gi|63146462|gb|AAY34059.1|         -----------------------------
gi|63146616|gb|AAY34136.1|         -----------------------------
gi|11138983|gb|AAG31571.1|         EINKRDGAYIELCVCSVEEAKKNTQEVVT
gi|73697858|gb|AAZ81532.1|         EINKRDGAYIELCVCSVEEAKKNTQEVVT
gi|73697846|gb|AAZ81526.1|         EINKRDGAYIELCVCSVEEAKKNTQEVVT
gi|12004297|gb|AAG43989.1|         EINKRDGAYIELCVCSVEEAKKNTQEVVT
gi|12004299|gb|AAG43990.1|         EINKRDGAYIELCVCSVEEAKKNTQEVVT
gi|73697866|gb|AAZ81536.1|         EINKRDGAYIELCVCSVEEAKKNTQEVVT
gi|73697848|gb|AAZ81527.1|         EINKRDGAYIELCVCSVEEAKKNTQEVVT
gi|73697856|gb|AAZ81531.1|         EINKRDGAYIELCVCSVEEAKKNTQEVVT
gi|73697862|gb|AAZ81534.1|         EINKRDGAYIELCVCSVEEAKKNTQEVVT
gi|160276|gb|AAA63423.1|           EINKRDGAYIELCVCSVEEAKKNTQEVVT
gi|73697844|gb|AAZ81525.1|         EINKRDGAYIELCVCSVEEAKKNTQEVVT
gi|339961368|pdb|3RRC|A            EINKRDGAYIELCVCSVEEAKKNTQEVVT
gi|339961369|pdb|3RRC|B            EINKRDGAYIELCVCSVEEAKKNTQEVVT
gi|255914751|dbj|BAH96577.1|       EINKRDGAYIELCVCSVEEAKKNTQEVVT
gi|224926624|gb|ACN69874.1|        EINKRDGAYIELCV---------------
gi|224926626|gb|ACN69875.1|        EINKRDGAYIELCV---------------
gi|224926628|gb|ACN69876.1|        EINKRDGAYIELCV---------------
gi|224926630|gb|ACN69877.1|        EINKRDGAYIELCV---------------
gi|224926632|gb|ACN69878.1|        EINKRDGAYIELCV---------------
gi|224926634|gb|ACN69879.1|        EINKRDGAYIELCV---------------
gi|224926636|gb|ACN69880.1|        EINKRDGAYIELCV---------------
gi|224926638|gb|ACN69881.1|        EINKRDGAYIELCV---------------
gi|224926640|gb|ACN69882.1|        EINKRDGAYIELCV---------------
gi|224926642|gb|ACN69883.1|        EINKRDGAYIELCV---------------
gi|224926644|gb|ACN69884.1|        EINKRDGAYIELCV---------------
gi|224926646|gb|ACN69885.1|        EINKRDGAYIELCV---------------
gi|224926648|gb|ACN69886.1|        EINKRDGAYIELCV---------------
gi|224926650|gb|ACN69887.1|        EINKRDGAYIELCV---------------
gi|224926652|gb|ACN69888.1|        EINKRDGAYIELCV---------------
gi|224926654|gb|ACN69889.1|        EINKRDGAYIELCV---------------
gi|224926656|gb|ACN69890.1|        EINKRDGAYIELCV---------------
gi|224926658|gb|ACN69891.1|        EINKRDGAYIELCV---------------
gi|224926660|gb|ACN69892.1|        EINKRDGAYIELCV---------------
gi|224926662|gb|ACN69893.1|        EINKRDGAYIELCV---------------
```

```
gi|224926664|gb|ACN69894.1|        EINKRDGAYIELCV---------------
gi|224926666|gb|ACN69895.1|        EINKRDGAYIELCV---------------
gi|224926668|gb|ACN69896.1|        EINKRDGAYIELCV---------------
gi|224926670|gb|ACN69897.1|        EINKRDGAYIELCV---------------
gi|224926672|gb|ACN69898.1|        EINKRDGAYIELCV---------------
gi|224926674|gb|ACN69899.1|        EINKRDGAYIELCV---------------
gi|224926676|gb|ACN69900.1|        EINKRDGAYIELCV---------------
gi|224926678|gb|ACN69901.1|        EINKRDGAYIELCV---------------
gi|224926680|gb|ACN69902.1|        EINKRDGAYIELCV---------------
gi|224926682|gb|ACN69903.1|        EINKRDGAYIELCV---------------
gi|224926684|gb|ACN69904.1|        EINKRDGAYIELCV---------------
gi|224926686|gb|ACN69905.1|        EINKRDGAYIELCV---------------
gi|224926688|gb|ACN69906.1|        EINKRDGAYIELCV---------------
gi|224926690|gb|ACN69907.1|        EINKRDGAYIELCV---------------
gi|224926692|gb|ACN69908.1|        EINKRDGAYIELCV---------------
gi|224926694|gb|ACN69909.1|        EINKRDGAYIELCV---------------
gi|224926696|gb|ACN69910.1|        EINKRDGAYIELCV---------------
gi|224926698|gb|ACN69911.1|        EINKRDGAYIELCV---------------
gi|224926700|gb|ACN69912.1|        EINKRDGAYIELCV---------------
gi|224926702|gb|ACN69913.1|        EINKRDGAYIELCV---------------
gi|224926704|gb|ACN69914.1|        EINKRDGAYIELCV---------------
gi|224926706|gb|ACN69915.1|        EINKRDGAYIELCV---------------
gi|224926708|gb|ACN69916.1|        EINKRDGAYIELCV---------------
gi|224926710|gb|ACN69917.1|        EINKRDGAYIELCV---------------
gi|224926714|gb|ACN69918.1|        EINKRDGAYIELCV---------------
gi|224926716|gb|ACN69919.1|        EINKRDGAYIELCV---------------
gi|224926718|gb|ACN69920.1|        EINKRDGAYIELCV---------------
gi|224926720|gb|ACN69921.1|        EINKRDGAYIELCV---------------
gi|224926722|gb|ACN69922.1|        EINKRDGAYIELCV---------------
gi|224926724|gb|ACN69923.1|        EINKRDGAYIELCV---------------
gi|224926726|gb|ACN69924.1|        EINKRDGAYIELCV---------------
gi|224926728|gb|ACN69925.1|        EINKRDGAYIELCV---------------
gi|224926730|gb|ACN69926.1|        EINKRDGAYIELCV---------------
gi|224926732|gb|ACN69927.1|        EINKRDGAYIELCV---------------
gi|224926734|gb|ACN69928.1|        EINKRDGAYIELCV---------------
gi|224926736|gb|ACN69929.1|        EINKRDGAYIELCV---------------
gi|224926738|gb|ACN69930.1|        EINKRDGAYIELCV---------------
gi|224926740|gb|ACN69931.1|        EINKRDGAYIELCV---------------
gi|224926742|gb|ACN69932.1|        EINKRDGAYIELCV---------------
gi|224926744|gb|ACN69933.1|        EINKRDGAYIELCV---------------
gi|224926746|gb|ACN69934.1|        EINKRDGAYIELCV---------------
gi|224926748|gb|ACN69935.1|        EINKRDGAYIELCV---------------
gi|224926750|gb|ACN69936.1|        EINKRDGAYIELCV---------------
gi|224926752|gb|ACN69937.1|        EINKRDGAYIELCV---------------
gi|224926754|gb|ACN69938.1|        EINKRDGAYIELCV---------------
gi|224926756|gb|ACN69939.1|        EINKRDGAYIELCV---------------
gi|224926758|gb|ACN69940.1|        EINKRDGAYIELCV---------------
gi|224926760|gb|ACN69941.1|        EINKRDGAYIELCV---------------
gi|224926762|gb|ACN69942.1|        EINKRDGAYIELCV---------------
gi|224926764|gb|ACN69943.1|        EINKRDGAYIELCV---------------
gi|224926766|gb|ACN69944.1|        EINKRDGAYIELCV---------------
gi|224926768|gb|ACN69945.1|        EINKRDGAYIELCV---------------
gi|224926770|gb|ACN69946.1|        EINKRDGAYIELCV---------------
gi|224926772|gb|ACN69947.1|        EINKRDGAYIELCV---------------
gi|224926774|gb|ACN69948.1|        EINKRDGAYIELCV---------------
gi|224926776|gb|ACN69949.1|        EINKRDGAYIELCV---------------
gi|224926778|gb|ACN69950.1|        EINKRDGAYIELCV---------------
gi|224926780|gb|ACN69951.1|        EINKRDGAYIELCV---------------
gi|224926782|gb|ACN69952.1|        EINKRDGAYIELCV---------------
```

```
gi|224926784|gb|ACN69953.1|         EINKRDGAYIELCV---------------
gi|224926786|gb|ACN69954.1|         EINKRDGAYIELCV---------------
gi|224926788|gb|ACN69955.1|         EINKRDGAYIELCV---------------
gi|224926790|gb|ACN69956.1|         EINKRDGAYIELCV---------------
gi|224926792|gb|ACN69957.1|         EINKRDGAYIELCV---------------
gi|224926794|gb|ACN69958.1|         EINKRDGAYIELCV---------------
gi|224926796|gb|ACN69959.1|         EINKRDGAYIELCV---------------
gi|224926798|gb|ACN69960.1|         EINKRDGAYSELCV---------------
gi|224926800|gb|ACN69961.1|         EINKRDGAYIELCV---------------
gi|224926802|gb|ACN69962.1|         EINKRDGAYIELCV---------------
gi|224926804|gb|ACN69963.1|         EINKRDGAYIELCV---------------
gi|224926806|gb|ACN69964.1|         EINKRDGAYIELCV---------------
gi|224926808|gb|ACN69965.1|         EINKRDGAYIELCV---------------
gi|224926810|gb|ACN69966.1|         EINKRDGAYIELCV---------------
gi|224926812|gb|ACN69967.1|         EINKRDGAYIELCV---------------
gi|224926814|gb|ACN69968.1|         EINKRDGAYIELCV---------------
gi|224926816|gb|ACN69969.1|         EINKRDGAYIELCV---------------
gi|224926818|gb|ACN69970.1|         EINKRDGAYIELCV---------------
gi|224926820|gb|ACN69971.1|         EINKRDGAYIELCV---------------
gi|213868281|gb|ACJ54187.1|         EINKRDGAYIELCVCSVEEAKKNTQEVVT
gi|213868283|gb|ACJ54188.1|         EINKRDGAYIELCVCSVEEAKKNTQEVVT
gi|213868285|gb|ACJ54189.1|         EINKRDGAYIELCVCSVEEAKKNTQEVVT
gi|213868287|gb|ACJ54190.1|         EINKRDGAYIELCVCSVEEAKKNTQEVVT
gi|213868289|gb|ACJ54191.1|         EINKRDGAYIELCVCSVEEAKKNTQEVVT
gi|213868291|gb|ACJ54192.1|         EINKRDGAYIELCVCSVEEAKKNTQEVVT
gi|213868293|gb|ACJ54193.1|         EINKRDGAYIELCVCSVEEAKKNTQEVVT
gi|213868295|gb|ACJ54194.1|         EINKRDGAYIELCVCSVEEAKKNTQEVVT
gi|213868297|gb|ACJ54195.1|         EINKRDGAYIELCVCSVEEAKKNTQEVVT
gi|213868299|gb|ACJ54196.1|         EINKRDGAYIELCVCSVEEAKKNTQEVVT
gi|213868301|gb|ACJ54197.1|         EINKRDGAYIELCVCSVEEAKKNTQEVVT
```
