# Supplementary figures and images for: Glycan Masking of Plasmodium vivax Duffy Binding Protein for Probing Protein Binding Function and Vaccine Development
Source: PLoS Pathog. 2013 Jun 13;9(6):e1003420. doi: 10.1371/journal.ppat.1003420 (PMC3681752; doi:10.1371/journal.ppat.1003420)

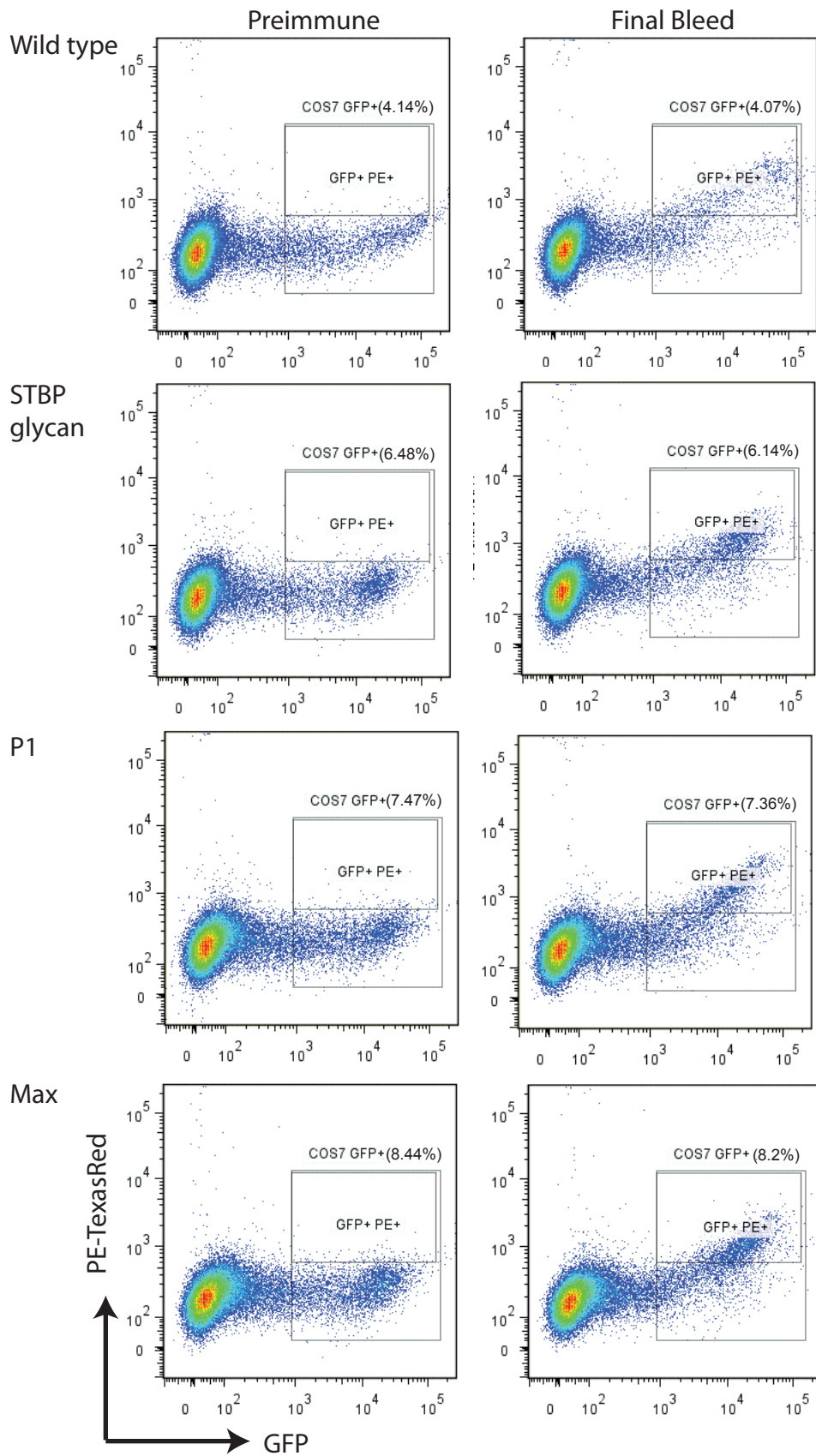

Supplement: Figure S3 — Transfection of COS-7 cells with PvDBPII-GFP fusion proteins. The gating strategy for GFP and anti-PvDBPII doubly positive transfected COS-7 cells is shown. The gate for GFP-positive cells was set by comparison to untransfected cells. The gate for anti-PvDBPII-positive (PE-Texas Red) was set by comparison to cells labeled with secondary antibody alone. (PDF) [file ppat.1003420.s003.pdf]

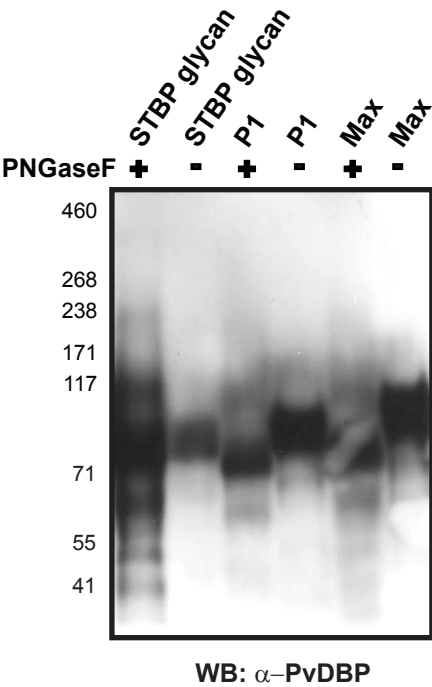

Supplement: Figure S4 — Western blot of DBPII glycosylation variants expressed in COS-7 cells with or without PNGaseF treatment. COS-7 cells transfected with recombinant DBPII glycosylation variants were lysed 48 h post transfection and immunoprecipitated with anti-GFP agarose resin. Half of the sample was subjected to PNGaseF treatment and the other half was untreated to observe glycosylation modifications. (PDF) [file ppat.1003420.s004.pdf]

**A** COS-7-RBC binding inhibition assay

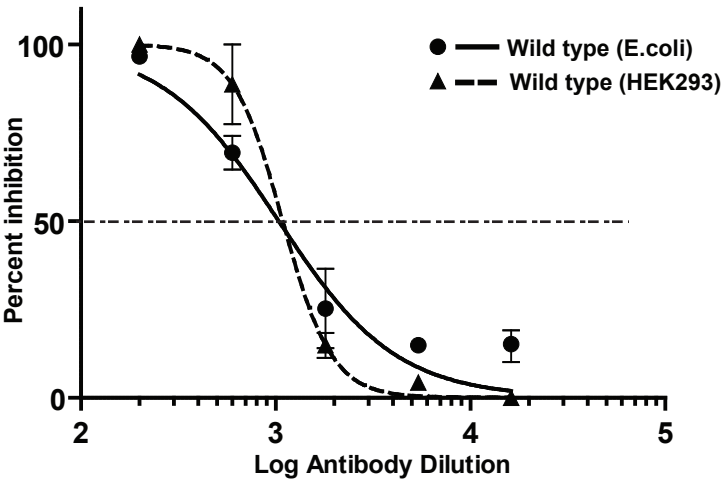

**B** Yeast display binding inhibition assay

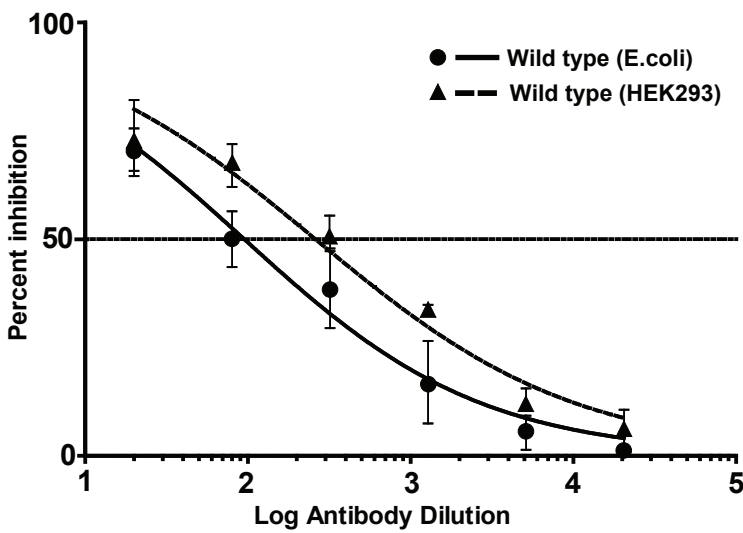

Supplement: Figure S5 — Inhibition of PvDBPII binding to DARC in different assay formats. Mice were immunized with wild-type DBPII protein produced in E. coli or HEK293 cells. Antibody inhibition of PvDBPII-DARC interaction in COS-7-RBC binding inhibition assay (A) and yeast display binding inhibition assay format (B). (PDF) [file ppat.1003420.s005.pdf]

**A**

Uninduced yeast + DARC Fc WT

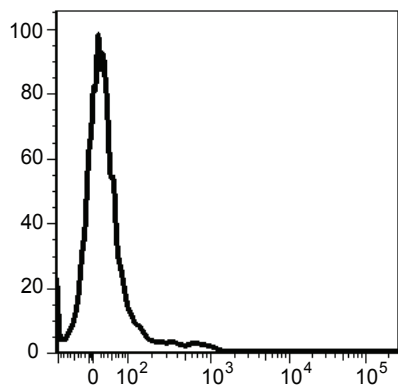**B**

Induced yeast + DARC Fc WT

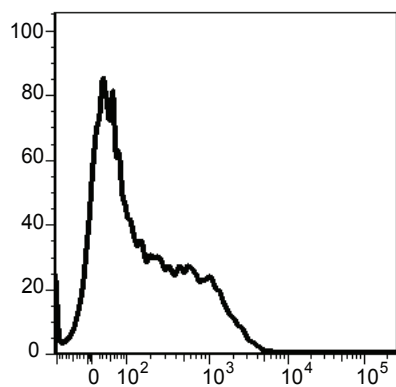**C**

Induced yeast + DARC Fc Y-&gt;F

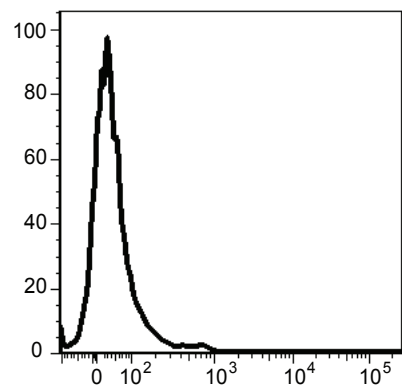

Supplement: Figure S6 — Yeast display antibody binding inhibition assay. Histograms showing DARC-Fc binding to yeast. (A) Uninduced yeast with no PvDBPII surface expression plus wild-type DARC-Fc (negative control). (B) Induced yeast with wild-type DARC-Fc (positive control). (C) Induced yeast with an inactive DARC-Fc mutant (negative control). (PDF) [file ppat.1003420.s006.pdf]
