# Supplementary material for: Glycan Masking of Plasmodium vivax Duffy Binding Protein for Probing Protein Binding Function and Vaccine Development
Source: PLoS Pathog. 2013 Jun 13;9(6):e1003420. doi: 10.1371/journal.ppat.1003420 (PMC3681752; doi:10.1371/journal.ppat.1003420)
Supplement: Table S1 — Adhesion of PvDBP mutants to DARC. (PDF) [file ppat.1003420.s007.pdf]

Table S1. Adhesion of PvDBP mutants to DARC

Mutation	Location[1]	Orientation of amino acid[2]	Reduction on DARC binding[6]	Reference
**Δ**198-216	SD1		none	Bolton et al. Virology J 2011
**Δ**198-247	SD1		major	Bolton et al. Virology J 2011
**Δ**198-216, **Δ**248-522	SD1, SD2, & SD3		major	Bolton et al. Virology J 2011
**Δ**216-247	SD1		major	Bolton et al. Virology J 2011
K220A,R223A,R226A	SD1	surface	major	Bolton et al. Virology J 2011
K222A	SD1	surface (^)[3]	none	Bolton et al. Virology J 2011
T232N	SD1	surface (*)[4]	none	this study
K234S	SD1	surface (^)	none	this study
**Δ**248-522	SD2 & SD3		major	Bolton et al. Virology J 2011
**E249R**	SD1	surface (^)	none	Batchelor et al. NSMB 2011
**E249R,R274E**	SD1 &SD2	surface (^)	moderate	Batchelor et al. NSMB 2011
L250A	SD1	buried	none	VanBuskirk et al. PNAS 2004
T251A	SD1	surface (*)	none	VanBuskirk et al. PNAS 2004
V254A	SD1	No info[5]	minor	VanBuskirk et al. PNAS 2004
N255A	SD1	No info	minor	VanBuskirk et al. PNAS 2004
N256A	SD1	No info	minor	VanBuskirk et al. PNAS 2004
T257A	SD1	No info	minor	VanBuskirk et al. PNAS 2004
D258A	SD1	No info	moderate	VanBuskirk et al. PNAS 2004
T259A	SD1	No info	moderate	VanBuskirk et al. PNAS 2004
N260A	SD1	No info	major	VanBuskirk et al. PNAS 2004
F261A	SD1	No info	major	VanBuskirk et al. PNAS 2004
D264N	SD1	No info	none	this study
T266A	SD2	surface (*)	none	VanBuskirk et al. PNAS 2004
F267A	SD2	surface (*)	none	VanBuskirk et al. PNAS 2004
L270A	SD2	surface (*)	none	VanBuskirk et al. PNAS 2004
Y271A	SD2	surface (^)	none	VanBuskirk et al. PNAS 2004
L272A	SD2	buried	major	VanBuskirk et al. PNAS 2004
**K273A**	SD2	surface (^)	major	VanBuskirk et al. PNAS 2004
**K273A**	SD2	surface (^)	moderate	Batchelor et al. NSMB 2011
**R274A**	SD2	surface (^)	none	Hans et al. Mol Micro 2005
**R274A**	SD2	surface (^)	moderate	Batchelor et al. NSMB 2011
**R274E**	SD2	surface (^)	major	Batchelor et al. NSMB 2011
K275A	SD2	surface (^)	moderate	VanBuskirk et al. PNAS 2004
L276A	SD2	buried	moderate	VanBuskirk et al. PNAS 2004
I277A	SD2	surface (*)	moderate	VanBuskirk et al. PNAS 2004
L290A	SD2	buried	none	VanBuskirk et al. PNAS 2004
**N291A**	SD2	surface (*)	moderate	Hans et al. Mol Micro 2005
**N291A**	SD2	surface (*)	moderate	VanBuskirk et al. PNAS 2004
N292A	SD2	surface (^)	none	VanBuskirk et al. PNAS 2004
**Y293A**	SD2	surface (^)	none	VanBuskirk et al. PNAS 2004
**Y293A**	SD2	surface (^)	minor	Hans et al. Mol Micro 2005
R294A	SD2	surface (^)	none	VanBuskirk et al. PNAS 2004

Y295A	SD2	surface (^)	major	VanBuskirk et al. PNAS 2004
N296A	SD2	surface (*)	moderate	VanBuskirk et al. PNAS 2004
K297A	SD2	surface (^)	major	VanBuskirk et al. PNAS 2004
**F299A**	SD2	buried	major	VanBuskirk et al. PNAS 2004
**F299A**	SD2	buried	major	Hans et al. Mol Micro 2005
D302A	SD2	buried	minor	VanBuskirk et al. PNAS 2004
I303A	SD2	buried	major	VanBuskirk et al. PNAS 2004
R304A	SD2	surface (^)	major	VanBuskirk et al. PNAS 2004
V327A	SD2	surface (^)	none	VanBuskirk et al. PNAS 2004
V328A	SD2	buried	none	VanBuskirk et al. PNAS 2004
E329A	SD2	surface (*)	major	VanBuskirk et al. PNAS 2004
N330A	SD2	surface (^)	major	VanBuskirk et al. PNAS 2004
D339A	SD2	surface (^)	minor	Hans et al. Mol Micro 2005
**E340A**	SD2	surface (^)	none	Hans et al. Mol Micro 2005
**E340A**	SD2	surface (^)	none	VanBuskirk et al. PNAS 2004
**K341A**	SD2	surface (^)	none	VanBuskirk et al. PNAS 2004
**K341N**	SD2	surface (^)	none	this study
**Q343A**	SD2	surface (^)	none	VanBuskirk et al. PNAS 2004
**Q343S**	SD2	surface (^)	none	this study
**Q344A**	SD2	surface (^)	none	VanBuskirk et al. PNAS 2004
**Q344A**	SD2	surface (^)	none	Hans et al. Mol Micro 2005
R345A	SD2	surface (^)	none	VanBuskirk et al. PNAS 2004
K347A	SD2	surface (^)	none	VanBuskirk et al. PNAS 2004
**Q348A**	SD2	surface (^)	none	VanBuskirk et al. PNAS 2004
**Q348A**	SD2	surface (^)	none	Hans et al. Mol Micro 2005
W350A	SD2	buried	major	VanBuskirk et al. PNAS 2004
**N351A**	SD2	surface (^)	minor	VanBuskirk et al. PNAS 2004
**N351A**	SD2	surface (^)	none	Batchelor et al. NSMB 2011
E352A	SD2	surface (^)	minor	VanBuskirk et al. PNAS 2004
K354A	SD2	surface (^)	none	Batchelor et al. NSMB 2011
Q356A	SD2	surface (^)	none	Batchelor et al. NSMB 2011
Y363A	SD2	surface (^)	major	Hans et al. Mol Micro 2005
S364A	SD2	surface (^)	moderate	VanBuskirk et al. PNAS 2004
V365A	SD2	surface (*)	moderate	VanBuskirk et al. PNAS 2004
K366A	SD2	No info	major	VanBuskirk et al. PNAS 2004
**K367A,K370A**	SD2	No info	none	Bolton et al. Virology J 2011
**K367A**	SD2	No info	major	VanBuskirk et al. PNAS 2004
R368A	SD2	No info	moderate	VanBuskirk et al. PNAS 2004
L369A	SD2	No info	major	VanBuskirk et al. PNAS 2004
K370A	SD2	No info	none	VanBuskirk et al. PNAS 2004
F373A	SD2	No info	major	Hans et al. Mol Micro 2005
I374N,I376T	SD2	No info	major	this study
I376A	SD2	No info	major	Hans et al. Mol Micro 2005
C377A	SD2	surface (*)	none	VanBuskirk et al. PNAS 2004
N384A	SD2	surface (^)	none	Batchelor et al. NSMB 2011
Q388A	SD3	buried	moderate	VanBuskirk et al. PNAS 2004
Y390A	SD3	buried	moderate	VanBuskirk et al. PNAS 2004

R391A	SD3	buried	minor	VanBuskirk et al. PNAS 2004
R394A	SD3	surface (^)	moderate	VanBuskirk et al. PNAS 2004
E395A	SD3	buried	moderate	VanBuskirk et al. PNAS 2004
W396A	SD3	buried	moderate	VanBuskirk et al. PNAS 2004
G397A	SD3	buried	minor	VanBuskirk et al. PNAS 2004
R398A	SD3	surface (^)	none	Hans et al. Mol Micro 2005
D399A	SD3	buried	minor	VanBuskirk et al. PNAS 2004
Y400A	SD3	buried	minor	VanBuskirk et al. PNAS 2004
V401A	SD3	surface (^)	minor	VanBuskirk et al. PNAS 2004
S402A	SD3	surface (^)	none	VanBuskirk et al. PNAS 2004
E403A	SD3	surface (^)	none	VanBuskirk et al. PNAS 2004
L404A	SD3	buried	none	VanBuskirk et al. PNAS 2004
P405A	SD3	surface (^)	none	VanBuskirk et al. PNAS 2004
T406A	SD3	surface (^)	none	VanBuskirk et al. PNAS 2004
E407A	SD3	surface (*)	none	VanBuskirk et al. PNAS 2004
V408A	SD3	buried	none	VanBuskirk et al. PNAS 2004
K412N	SD3	surface (^)	none	this study
E413A	SD3	surface (^)	none	VanBuskirk et al. PNAS 2004
**K414A**	SD3	surface (^)	none	VanBuskirk et al. PNAS 2004
**K414S**	SD3	surface (^)	none	this study
I419A	SD3	surface (^)	none	VanBuskirk et al. PNAS 2004
N420A	SD3	surface (*)	none	VanBuskirk et al. PNAS 2004
K425A	SD3	surface (^)	none	VanBuskirk et al. PNAS 2004
V426A	SD3	surface (*)	none	VanBuskirk et al. PNAS 2004
E464S	SD3	surface (^)	none	this study
Q467N	SD3	surface (^)	none	this study
A469T	SD3	surface (^)	none	this study
V488T	SD3	surface (^)	none	this study
R497S	SD3	surface (^)	none	this study
D264N,E464S,V488T	SD2 & SD3	surface	none	this study
T232N,K234S,D264N,K341N, Q343S,K412N,K414S,Q467N, A469T,V488T,R497S	SD1, SD2, & SD3	surface	none	this study

[1], subdomain designations according to Singh et al. Nature 2006

[2],designation of orientation of amino acid is according to 3RRC.pdb

[3], ^ means surface exposed "R" group

[4], * means buried amino acid or the "R" group is not directed outward

[5], no info means no structural information is available due to the absence of density in PvDBPII crystal structure 3RRC.pdb

[6], reduction of DARC binding is as described in Hans et al., Bolton et al., Batchelor et al., and this study where none is 70-100% binding, minor is 50-70% binding, moderate is 30-50% binding, and major is less than 30% binding. Reduction of DARC binding for VanBuskirk et al. is as described in their manuscript where none is 90-100% binding, minor effect is 50-90% binding, moderate is 5-50% binding, and major is 0-5% binding
