# Supplementary material for: Glycan Masking of Plasmodium vivax Duffy Binding Protein for Probing Protein Binding Function and Vaccine Development
Source: PLoS Pathog. 2013 Jun 13;9(6):e1003420. doi: 10.1371/journal.ppat.1003420 (PMC3681752; doi:10.1371/journal.ppat.1003420)
Supplement: Table S2 — Immunization schedule. (PDF) [file ppat.1003420.s008.pdf]

TABLE S2. Immunization schedule

Immunization	Immunogen	Group	Animal	No. of animals	No. of Immunizations		Dose (μg)	
					DNA	Protein	DNA	Protein
Protein[1]	Wt (*E. coli* refolded)	1	Mouse	10		4		10
Protein[1]	Wt (HEK293)	2	Mouse	10		4		5
DNA[2],Protein[1]	Wt (HEK293)	3	Mouse	10	4	2	20	2.5
DNA[2],Protein[1]	STBP glycan	4	Mouse	10	4	2	20	2.5
DNA[2],Protein[1]	P1	5	Mouse	10	4	2	20	2.5
DNA[2],Protein[1]	MAX	6	Mouse	10	4	2	20	2.5

[1], immunization was intramuscular

[2], immunization was by DNA electroporation
