# Supplementary material for: Glycan Masking of Plasmodium vivax Duffy Binding Protein for Probing Protein Binding Function and Vaccine Development
Source: PLoS Pathog. 2013 Jun 13;9(6):e1003420. doi: 10.1371/journal.ppat.1003420 (PMC3681752; doi:10.1371/journal.ppat.1003420)
Supplement: Table S3 — IC50 of DBPII glycosylation variants. (PDF) [file ppat.1003420.s009.pdf]

Table S3. IC50 of DBPII glycosylation variants.

Immunogen	COS-7-RBC binding inhibition assay		Yeast display binding inhibition assay		Effect of DARC phenotype			
					FyA		FyB	
	IC50[1]	SD[2]	IC50	SD	IC50	SD	IC50	SD
Wild type (HEK293)[3]	2589	8.03	32.71	21.45	1203	34.85	442.7	33.6
STBP glycan[3]	2787	13.82	34.12	23.32	435.9	32.67	408	28.92
P1[3]	2837	10.11	199.4	31.01	1237	40.14	459.7	42.91
Max[3]	2110	8.53	668.6	27.5	926.7	35.72	273.4	35.31

1, plasma dilution at 50% inhibition
2, standard deviation
3, DNA immunization,protein boost
